# A Gradient Boosting-Based Machine Learning Model for the Prediction of the Hardness of Thermoplastic Polymers

**DOI:** 10.1021/acsomega.6c07307

**Published:** 2026-10-21

**Authors:** Jose Sena, Jonny J. Blaker, Sam Hay

**Affiliations:** † Department of Chemistry and Manchester Institute of Biotechnology, 5292 The University of Manchester, Manchester M1 7DN, U.K.; ‡ Department of Materials and Henry Royce Institute, The University of Manchester, Manchester M13 9PL, U.K.; § Department of Chemistry, Autonomous University of Santo Domingo, Santo Domingo 10103, Dominican Republic

## Abstract

Modern experimental techniques can measure the hardness of thin films and structural features on the nanometer scale. These techniques can be applied to many types of materials, but as the hardness of the material decreases, more specialized and precise equipment is needed. For polymers, this analysis can be relatively expensive and time-consuming, so here we demonstrate a gradient-boosting-based machine-learning model that can provide a fast and reliable prediction of the hardness of thermoplastic polymers. This model is based on the XGBoost algorithm and shows reasonably good performance with an *R* ^2^ value of 0.46–0.69. The features used in this study include experimental (mechanical and thermal properties) and molecular descriptors obtained using RDKit. The model is robust, with reasonable performance obtained when key experimental features are removed and/or when the selection of polymers is reduced. This suggests that this approach will be useful in “bottom-up” strategies for polymeric materials development.

## Graphical abstract

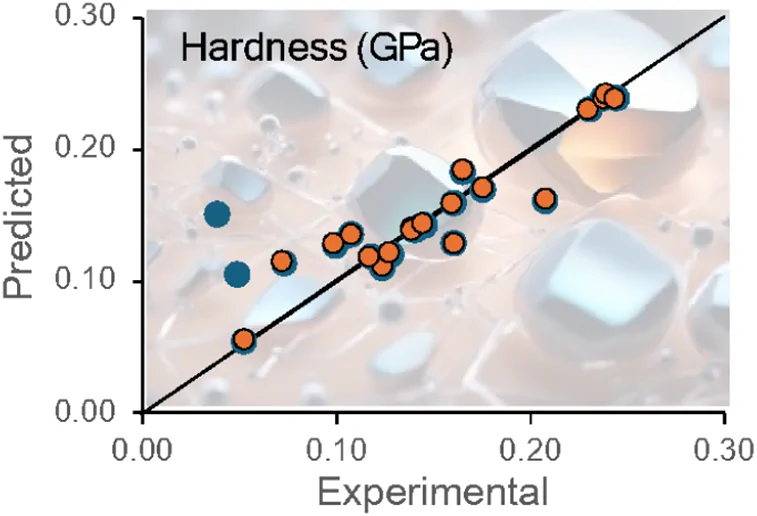

## Introduction

Hardness is one of the most measured material properties.^1^ The basis of hardness testing is to measure the material’s surface resistance to deformation by a rigid indenter with a defined geometry that is harder than the material being tested. This method is used for a wide range of materials, including metals, ceramics, polymers, and composites.^2^ The analysis of indentation load-penetration curves produced by depth-sensing indentation systems is often based on work by Oliver and Pharr,^3^ who showed that the unloading curve changes constantly due to a changing contact area and realized that a derivative of the unloading-curve power-law expression can yield information about the state of the contact point at the maximum load.^2,3^ State-of-the-art measuring equipment allows the measurement of hardness at the nanometer scale on thin films and small structural features,^3^ but as the hardness of the material decreases, more precise equipment is needed, and the average time between tests increases.^4^

The application of machine learning (ML) algorithms to problems pertaining to materials science has been growing in the last two decades.^5–9^ For example, Khakurel et al.^5^ predicted the Young’s modulus of a series of refractory alloys of complex composition using tree gradient boosting. Wang et al.^6^ used an assortment of algorithms (Support Vector Regression, Random Forest Regression, and Backpropagation Neural Network) to assess the hardness of multilayered Al/Si_3_N_4_ composites. Mambuscay et al.^7^ used monochromatic images of indentation imprints on D2 steels (1.50%C, 12%Cr) with different coatings and heat treatments for a quick prediction method based on Random Forest Regression. The prediction of hardness of oxide glasses (Al_2_O_3_, Li_2_CO_3_, Na_2_CO_3_, CaCO_3_, and Mg_2_(OH)_2_CO_3_) was demonstrated by Tian et al.^10^ using Support Vector ML and the least absolute shrinkage and selection operator (LASSO). For polymeric composites, Champa-Bujaico^11^ used various ML algorithms to predict and optimize different mechanical properties of the composite. For the prediction of Young’s modulus and elongation at break, a recurrent neural network (RNN) proved to be the most accurate, whereas Random Forest was the best algorithm for the prediction of tensile strength. Other properties that can be predicted using ML include Joule heating,^12^ glass transition temperature,^13,14^ surface free energy^15^ and cloud point for thermoresponsive polymers.^16^

Gradient boosting with regression trees produces highly robust ML models that are especially appropriate for relatively small data sets.^17^ These algorithms work by estimating a function from the perspective of numerical optimization in function space rather than parameter space; i.e., rather than searching for the exact solution in a given search space, an initial approximation of the function is optimized to provide an acceptable prediction.^18^ Different extensions to this method have been developed, with Friedman^14^ adding randomization of data subsets selected for each iteration of the initial function to increase prediction accuracy and code execution speed. Chen and Guestrin^17^ presented an easily scalable tree-boosting system that can handle a wide range of dataset sizes and still be able to provide acceptable accuracy. In the current study, we developed a gradient-boosting ML model to predict the hardness of thermoplastic polymers using a range of features that include molecular descriptors and experimental data from the literature and in-house experiments.

## Materials and Methods

Polymer films and sheets were obtained from Goodfellow Cambridge Limited, UK. A soft, microfine tissue towel moistened with isopropyl alcohol was used to clean the surface prior to any experiment to prevent contamination from manufacturing, handling, or sampling. White-light interferometry measurements were carried out using a Bruker Contour GTR-K1 interferometer to determine the surface profiles of the materials tested. Differential scanning calorimetry (DSC) measurements were performed with a TA Instruments DSC 250, using a sample weight of 5–10 mg. Nanoindentation measurements were performed using a Hysitron TI 980 system equipped with a 10 μm conical indenter tip. Nineteen different polymers were selected for measurement, and additional experimental data were obtained from the literature. Table 1 gives the full list of the polymers used in this study, their engineering properties, and hardness, obtained from in-house measurements and literature.

**Table 1:** Polymers Used in This Study and Their Reported/Measured Hardness^a^

	Polymer	Category	Hardness (GPa)
1	Polyether ether ketone (PEEK)	High-performance thermoplastic	0.230 ± 0.022^b^
2	Polyphenylene sulfide (PPS)	High-performance thermoplastic	0.160 ± 0.010^b^
3	Polysulfone (PSU)	High-performance thermoplastic	0.038 ± 0.002^b^
4	Polycarbonate (PC)	Engineering thermoplastic	0.208 ± 0.006^b^
5	Polyethylene (PE)	Standard thermoplastic	0.073 ± 0.017^b^
6	Polypropylene (PP)	Standard thermoplastic	0.099 ± 0.012^b^
7	Polyethylene terephthalate (PET)	Engineering thermoplastic	0.160 ± 0.014^b^
8	Poly(methyl methacrylate) (PMMA)	Engineering thermoplastic	0.238 ± 0.025^b^
9	Acrylonitrile butadiene styrene (ABS)	Copolymer	0.175 ± 0.027^b^
10	Fluorinated ethylene propylene (FEP)	Copolymer	0.052 ± 0.004^b^
11	Polycaprolactam aramid 6 (Nylon 6)	Engineering thermoplastic	0.107 ± 0.003^b^
12	Poly(1,3-butadiene) (PBD)	Standard thermoplastic	0.140 ± 0.008^c^
13	Polychlorotrifluoroethylene (PCTFE)	Halogen derivate of PE	0.124 ± 0.003^b^
14	Polyisobutylene (PIB)	Standard thermoplastic	0.139 ± 0.019^c^
15	Polystyrene (PS)	Standard thermoplastic	0.239 ± 0.009^b^
16	Polytetrafluoroethylene (PTFE)	Fluorinated derivate of PE	0.048 ± 0.004^b^
17	Polyvinyl alcohol (PVOH)	Derivate of PVA	0.139 ± 0.019^c^
18	Polyvinyl chloride (PVC)	Standard thermoplastic	0.244 ± 0.006^b^
19	Polyvinyl fluoride (PVF)	Fluorinated derivate of PVC	0.145 ± 0.019^c^
20	Polyvinylidene fluoride (PVDF)	High-performance thermoplastic	0.117 ± 0.034^b^
21	Polyether sulfone (PES)	High-performance thermoplastic	0.165 ± 0.010^b^
22	Polyphenylene oxide (PPO)	High-performance thermoplastic	0.144 ± 0.019^b^
23	Polybutylene terephthalate (PBT)	Engineering thermoplastic	0.128 ± 0.011^b^

a Chemical structures are given in Figure S1 in the Supporting Information.

b Data obtained from in-house measurements.

c Experimental data obtained from the literature, PBD from Špírková et al.,^19^ PIB from Kulkarni et al.,^20^ PVOH from Jha et al., and PVF from Wang et al.^21^

Results from Špírková et al.^19^ and Jha and Shimpi^22^ were obtained by nanoindentation, thus making them directly comparable with our in-house measurements. Hardness results reported by Kulkarni et al.^20^ and Wang et al.^21^ were obtained by AFM, and these can be compared with nanoindentation results, as they often agree within experimental uncertainty.^23^

Hardness values reported for polymers can vary depending on several experimental and material factors. Differences in nanoindentation parameters, such as indenter geometry, maximum load, and loading rate, may influence the absolute values, but the cited studies and our in-house measurements employed standard Oliver–Pharr analysis under comparable load ranges, ensuring methodological consistency. Sample preparation and surface conditions, including polishing and roughness, are other sources of variability; however, both the literature and in-house data were obtained from specimens prepared to minimize surface defects.

The ML data set is composed of 23 polymers (Table 1) and 11 features that were selected based on our previous work^24^ and available molecular descriptors (Table 2). The hardness of each polymer was determined using nanoindentation measurements, which also yielded stiffness values for each polymer, and these were used as an input feature in some models. Differential scanning calorimetry (DSC) was also performed on each polymer. As the polymers contain both amorphous (40% of samples) and semicrystalline (60% of samples) materials, only the glass transition temperature (*T*_g_) obtained from DSC was used as an input feature; the melting temperature (*T*_m_) was not used, as this cannot be determined for the amorphous polymers. The crystallization percentage (%Crystallization) was calculated according to eq 1 ^25^ and used as an input feature:

1 $$\%Crystallization=\left(\Delta H_{c}/\Delta H_{c}^{0}\right)\times 100\%$$

where Δ*H*_c_ is the crystallization enthalpy and Δ*H*_c_^0^ represents the enthalpy of a 100% crystalline polymer, whose values are widely available in the literature.^26–31^ An additional experimental input feature, surface roughness, was measured using white light interferometry (Table S1 in the Supporting Information) and complemented with values from the literature.^32,33^ The surface energy (γ) was also selected as an ML feature because it is a property of the material surface, with values obtained from literature.^34–38^

**Table 2:** List of Features Used in This Work

Feature index^a^	Feature name
F2	Stiffness (μN/nm)^b^
F3	Surface energy (mJ/m^2^)^b^
F4	Glass transition temperature (°C)^b^
F5	Crystallinity percentage^b^
F6	Surface roughness (nm)^b^
F7	sp^3^ hybridization^c^
F8	sp^2^ hybridization^c^
F9	Non-H atoms^c^
F10	Trimer molecular weight^c^
F11	Number of hydrogen atoms^c^
F12	Number of valence electrons^c^

a F1 is an identification value that is not used as a training feature.

b Experimental data.

c Molecular descriptors obtained using RDKit.

Aside from these five experimental features, six additional features were added (Tables 2 and S2). These are molecular descriptors obtained using RDKit^39^ from the SMILES string corresponding to a trimer of each polymer (Figure S1). The number of hybridized sp^2^ and sp^3^ orbitals was chosen to account for the aromatic rings and the saturation level of the hydrocarbon’s chains. The numbers of hydrogen and non-hydrogen atoms were also computed, along with the number of valence electrons and the molecular weight of the trimer.

As the output (hardness) is a continuous variable, models based on supervised learning were explored to choose the model that best predicted this output by using selections of the input features described above. These models included decision trees, random forests, and gradient boosting, specifically XGBoost.^17^

The different ML algorithms were evaluated according to our previous work,^24^ using (i) Root Mean Square Error (RMSE), (ii) Mean Absolute Error (MAE), (iii) and Coefficient of Determination (*R* ^2^) using the following equations:

$$\mathrm{RMSE}\left(y,ŷ\right)={\left(\frac{1}{n_{\mathrm{samples}}}\overset{n_{\mathrm{samples}}-1}{\underset{i=0}{\sum }}{\left(y_{i}-{ŷ}_{i}\right)}^{2}\right)}^{1/2}$$ 2

In eqs 2, 3, and 4, *ŷ* refers to the experimental values, and *y* refers to the predicted values.

3 $$\mathrm{MAE}\left(y,ŷ\right)=\frac{1}{n_{\mathrm{samples}}}\overset{n_{\mathrm{samples}}-1}{\underset{i=0}{\sum }}\left|y_{i}-{ŷ}_{i}\right|$$

The mean absolute error is calculated as the sum of the differences between each predicted value and its corresponding experimental value. The quotient between this sum and the number of samples is the result.

4 $$R^{2}\left(y,ŷ\right)=1-\frac{\overset{n}{\underset{i=1}{\sum }}{\left(y_{i}-{ŷ}_{i}\right)}^{2}}{\overset{n}{\underset{i=1}{\sum }}{\left(y_{i}-\mathrm{y̅}\right)}^{2}}$$

The coefficient of determination, usually denoted as *R* ^2^, provides a measure of how good the predictions of unseen values would be. The maximum value of this metric is 1, and it can also take negative values when the model’s predictions are arbitrarily poor.

## Results and Discussion

The *leave-one-out cross-validation* (LOOCV) method^40^ was used for model training and testing, with the process repeated 23 times to sequentially predict the hardness of each polymer in the study. The full data set is given in Tables S1 and S2. The initial choice of ML models to tackle the prediction of hardness was based on a literature survey in which most ML applications to materials were focused on tree-based models.^41–44^ Thus, ML models using Decision Tree, Random Forest, and XGBoost algorithms were tested (Tables 3 and S3). The XGBoost-based method was then chosen for further optimization, as it gave the best initial performance. This can be attributed to the small size of the data set and its tabular nature, whereas for relatively large data sets composed of text and images, neural networks tend to perform better.^45^

**Table 3:** Metrics for Different ML Algorithms Explored in This Work

	RMSE	MAE	*R* ^2^
Decision tree	0.054	0.037	0.25
Random forest	0.050	0.033	0.0.34
XGBoost	0.046^a^	0.035^a^	0.39^a^
	0.047^b^	0.030^b^	0.54^b^
	0.039^b^,^c^	0.025^b^,^c^	0.69^b^,^c^

a XGBoost models with default hyperparameters given in Table S4.

b XGBoost models with optimized hyperparameters given in Table S4.

c XGBoost models when PSU and PTFE are omitted from the analysis.

Optimization of three XGBoost hyperparameters was explored: (i) the number of estimators (n_estimators) parameter controls the number of trees in the model. Usually, a high number of trees is desirable, but this can lead to overfitting. The default value is 100; (ii) the maximum tree depth (max_depth) controls the maximum depth of the trees; as the model gets deeper, it also grows in complexity, which in turn can lead to overfitting. However, a small number of trees can produce an underfitted model. The default value is 6; (iii) the learning rate (eta) controls the rate at which the regressor updates the feature weights. A small value produces a slowly converging model that can sometimes output a better prediction, while a high value makes a faster model but, as with previous parameters, can lead to overfitting. The default value is 0.3. These three hyperparameters were partially optimized using search ranges of 10–100 for n_estimators, 1–10 for max_depth, and 0.1–0.7 for eta. The final values are given in Table S4. This optimization was carried out by holding the other hyperparameters at their default values and by minimizing the difference between the predicted and experimental hardness within the search range of the hyperparameter of interest. Nested cross-validation was also performed on the model using the optimized hyperparameters. While showing variable performance, likely due to the relatively small size of the data set,^46^ this additional validation yields comparable results to the LOOCV across some folds (Tables S6 and S7). The model output, experimental hardness vs. predicted hardness, is shown in Figure 1. The performance of the model was evaluated according to eqs 2–4, showing good performance with an *R* ^2^ value of 0.54.

**Figure 1:**
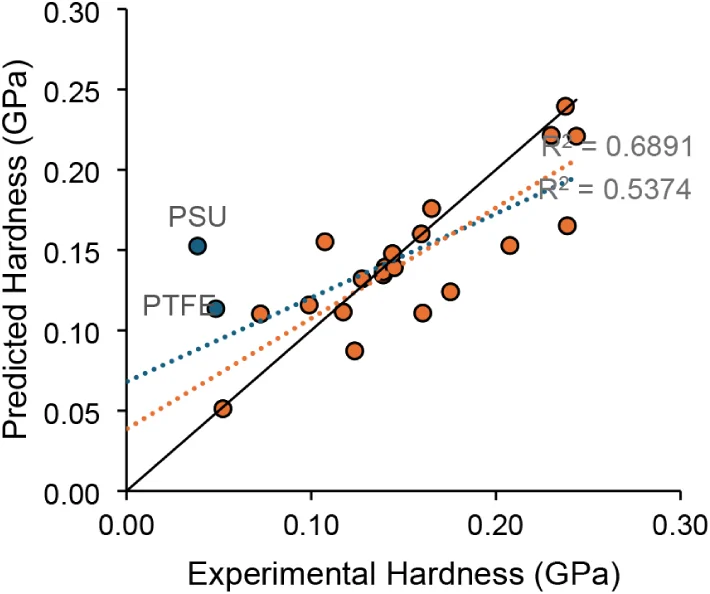
Output of the optimized XGBoost ML model trained using all features in Table 2, showing predicted vs. experimental hardness values. The dashed blue line represents the prediction for the 23 polymers listed in Table 1 obtained using the LOOCV method. The red dashed line is the prediction excluding two polymers, PSU and PTFE. The diagonal black line corresponds to *x* = *y.*

Next, the relative importance of each feature was investigated using the total gain function in XGBoost, which was averaged across the 23 LOOCV model predictions. Figure 2 shows the gain of each feature, and an inspection of these data indicates that the most important features are stiffness and glass transition temperature. Stiffness is a measure of an object’s resistance to deformation when an external force is applied. Stiffness is also obtained from nanoindentation experiments, and it is partially positively correlated with hardness values in our data set (*R* ^2^ = 0.57; Figure S3). The number of hybridized orbitals is ranked in the third and fourth places, suggesting that the extent of double bonding in the polymer structure has an impact on overall hardness. The crystallinity percentage is ranked sixth and has similar gain values to the surface properties, surface roughness and surface energy.

**Figure 2:**
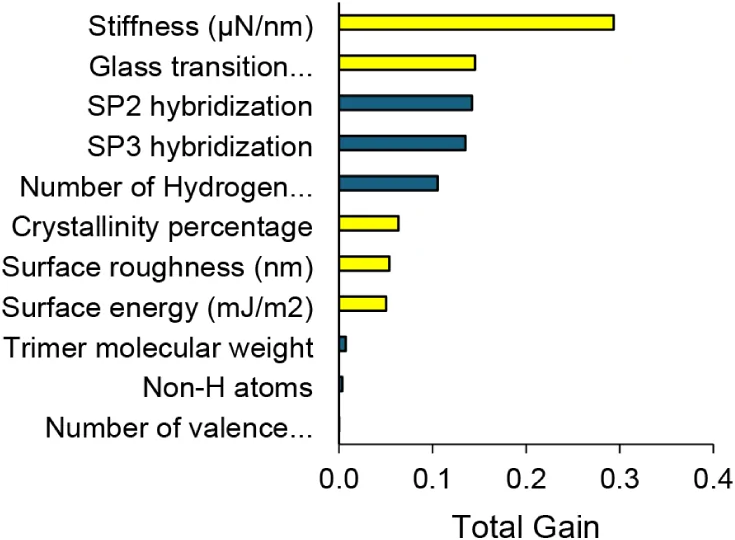
Relative feature importance for the optimized XGBoost ML model. Experimental features are presented in yellow, while the molecular descriptors obtained with RDKit are in blue. Total gain values are given in Table S9.

Additional graphical analyses were performed to assess the model’s reliability and performance. The cumulative frequency of residual errors was calculated, and the results are shown in Figure 3. For this analysis, a steeper line corresponds to higher predictive power, while a flatter line indicates a model with low predictive power. Our optimized model shows a moderately steep line, thus showing an acceptable predictive power.^47^

**Figure 3:**
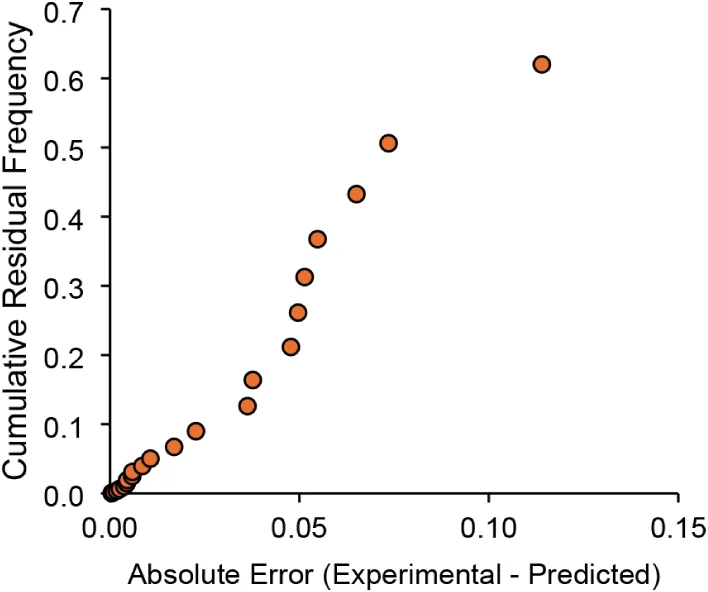
Cumulative frequency plot of residual error for predicting polymer hardness from experimental and computational data. This plot was constructed with the experimental and predicted values from the optimized XGBoost model using all features. These values are presented in Table S8.

The cumulative residual frequency is calculated by first finding the cumulative frequency of each input and then subtracting it from the total number of observations. It shows how many values are left after accounting for all of the frequencies up to a certain point. This measure decreases as you move through the distribution, since fewer observations remain, and it provides a way to understand the tail of the data set by showing how the frequencies diminish across successive inputs.^47^

To evaluate the applicability domain of our model, a William’s plot is also presented. This analysis is widely used to assess model robustness^48^ and detect data points that could adversely impact the model’s predictive power. The William’s plot for our optimized XGBoost model trained with all features is presented in Figure 4. For our trained model, all compounds have leverage values below the warning leverage line, and most compounds fall within the standardized residual limits of +3 and −3 standard deviations. PSU has a standardized residual below −3, indicating a response outlier in accordance with the results presented in Figure 1. Interestingly, PTFE is not identified as an outlier. For our model, the warning leverage value is unusually high, as our data set contains 23 inputs and 11 features, which makes leverage-based detection less rigid; typical values for the warning leverage are between 0.2 and 0.5.^47^

**Figure 4:**
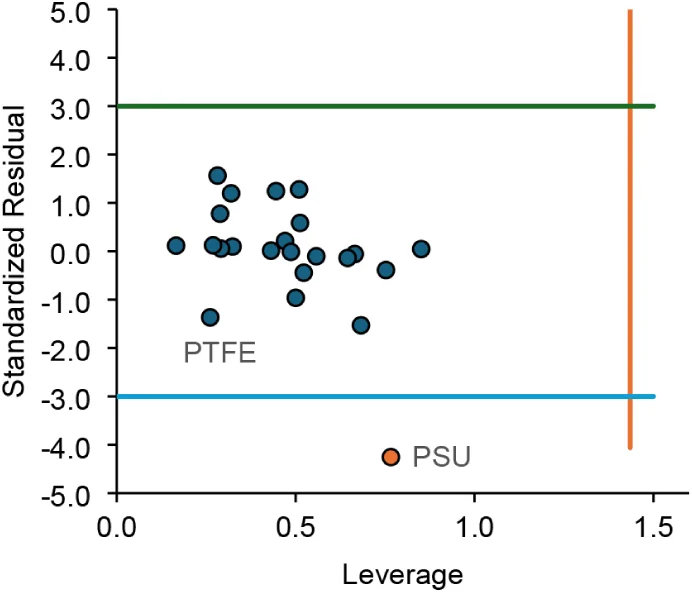
William’s plot for the optimized XGBoost model. In this analysis, data points above and below three standard deviations (blue lines) are considered to differ markedly from the bulk of the data and are classified as outliers, while data points beyond the warning leverage line (orange) are outliers undergoing substantial extrapolation. Values for this graph are taken from Table S9.

To assess the reliability of the XGBoost model without the stiffness feature, the model was retrained without this feature, again using the LOOCV method and hyperparameter optimization following the same procedure as the previous model (Table S5). The model output and feature importance are presented in Figure 5, with metrics given in Table 4. As expected, the model’s performance is reduced (*R* ^2^ = 0.39). However, this performance may be acceptable in situations in which stiffness data are unavailable. Here, the feature importance is shifted in favor of surface roughness as the most important feature, with SP3 orbital hybridization in second position. The crystallinity percentage and the glass transition temperature are in fourth and fifth place, respectively.

**Figure 5:**
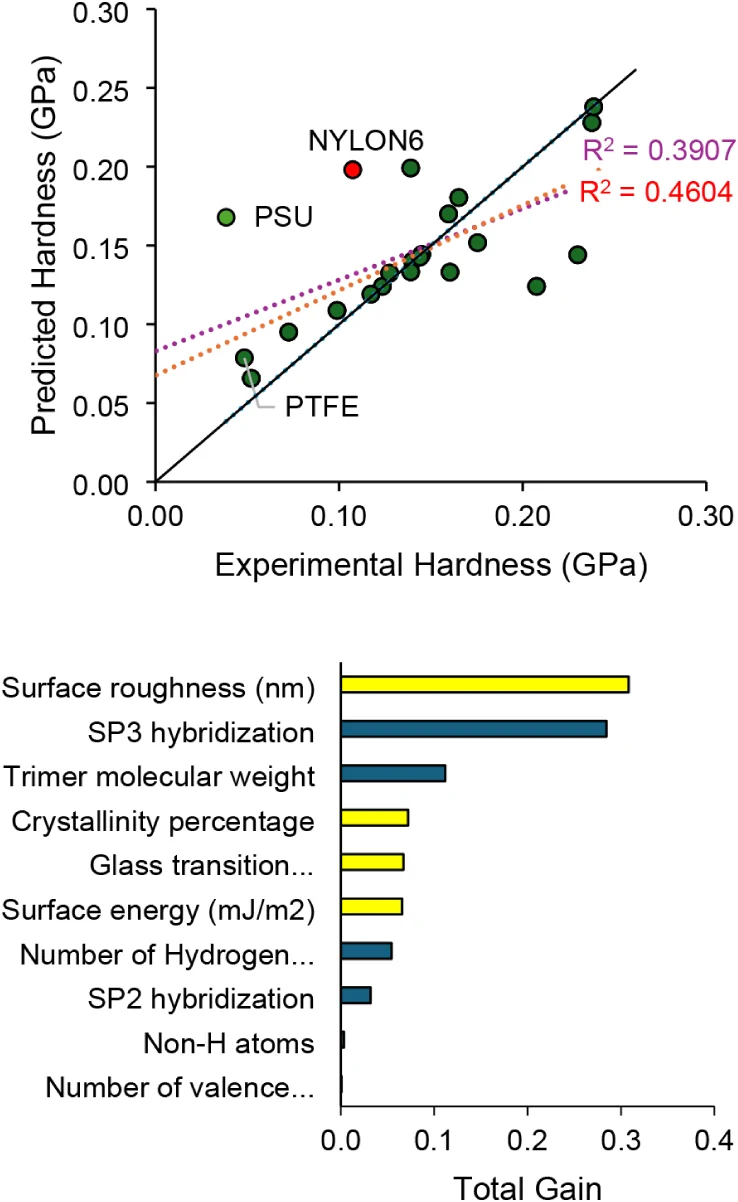
Output of the XGBoost ML model trained without the stiffness feature. *Top,* Predicted vs. experimental hardness. The dashed purple line represents the predictions for all 23 polymers; the dashed red line is the prediction excluding two polymers (PSU and PTFE). *Bottom,* Relative feature importance for the model trained with all 23 polymers, with experimental features presented in yellow and molecular descriptors obtained with RDKit in blue. Total gain values are given in Table S11.

**Table 4:** Percentage Error (%*e*) for the Two Polymers with Lowest Hardness^a^

	Hardness (GPa)		
Polymer	Experimental	Predicted^b^	%*e* ^b^
PSU	0.038	0.153, 0.168	296, 336
PTFE	0.045	0.114, 0.079	134, 63

a The complete list is given in Tables S8 and S10 in the Supporting Information.

b The second values were determined by using the model trained without the stiffness feature.

The XGBoost models consistently show possible outliers in the predicted vs. experimental hardness behavior. In Figures 1 and 5, there are two data points that could be treated as outliers. These are polysulfone (PSU) and polytetrafluoroethylene (PTFE). These two polymers are the softest polymers (lowest hardness) in the data set and, according to our previous analysis, can be considered outliers. To better visualize their behavior, a new metric was used: percentage error (%*e*), which was calculated by

5 %e=(|exp.value−pred.value|/exp.value)×100

These percentage error values are given in Tables 4, S8 and S10.

The large %*e* values for PSU and PTFE may arise from their low hardness values. By treating PSU and PTFE as outliers and excluding them from the model evaluation, the prediction metrics are significantly improved, with *R* ^2^ values of 0.69 and 0.46 for the models trained with and without stiffness, respectively (Figures 1 and 5, Table 3). While it is not clear why our models consistently overestimate the hardness of PSU and PTFE, it appears that the models fail to handle very low (<50 MPa) hardness values.

To explore further model optimization, we next examined the importance of surface energy as a feature. This is the only feature for which the values were obtained entirely from literature sources, so we reasoned that these may contain more uncertainty than those of other experimental features. Figure S7 shows a model trained without stiffness or surface energy as input features. The model performance is similar to that in Figure 5, where the model was trained without the stiffness feature, suggesting that surface energy is not an essential feature. As in previous models, PSU and PTFE are outliers, and by removing them, the model’s predictive power increases from *R* ^2^ = 0.55–0.65.

Finally, the model was retrained with a subset of polymers selected based on polymer’s morphology. The default model hyperparameters were used in this case. Using only the semicrystalline entries in the data set (%Crystallinity > 0; *n* = 13), the performance was poor (*R* ^2^ = 0.034; Table 5). However, when the model was trained using only the amorphous polymers (%Crystallinity = 0; *n* = 10), reasonable performance was obtained (*R* ^2^ = 0.58; Table 5). Interestingly, inspection of the feature importances shows that this model is dominated by surface roughness, stiffness, and glass transition temperature (Figure 6, Table S12). Finally, the model was also trained and evaluated using the full data set of polymers but only the molecular descriptor features obtained from RDKit. This produced poor prediction (*R* ^2^ = 0.20; Table 5), highlighting the importance of the experimental features used in this study.

**Table 5:** Model Exploration of Different Compositions of the Data Set and Feature Selection

	Morphology		
Model	Semicrystalline	Amorphous	Molecular descriptors only
RMSE	0.055	0.043	0.055
MAE	0.039	0.027	0.040
*R* ^2^	0.034	0.58	0.20

**Figure 6:**
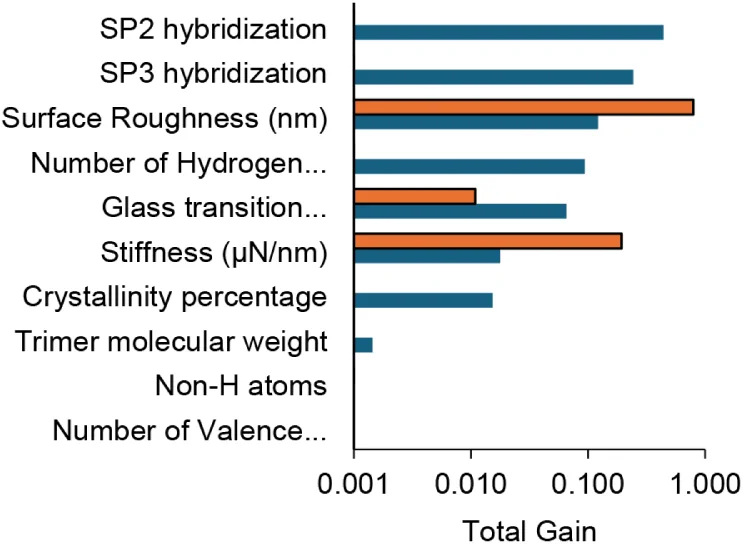
Relative feature importance for the XGBoost ML model trained on polymers of different morphology. Note the log scale. The orange bars represent the prediction using only amorphous polymers and the blue bars the prediction using only semicrystalline polymers. Values are given in Table S12.

## Conclusions

A regression ML model based on the XGBoost algorithm was developed to predict the hardness of thermoplastic polymers. When using a combination of experimental and computational features, the ML model showed useful performance despite the relatively small data set used (*n* = 23), particularly when the model hyperparameters were optimized. The model achieved reasonably strong predictive performance, with *R* ^2^ values ranging from 0.46 to 0.69 depending on the features used for training. Limitations were observed for polymers with very low hardness (below 50 MPa) and when the data set consisted only of semicrystalline polymers. Nonetheless, the overall findings highlight that gradient boosting methods can provide rapid, reliable estimates of polymer hardness. Feature importance analysis revealed that stiffness and surface energy were the most influential predictors of hardness, with glass transition temperature and crystallinity percentage also contributing meaningfully. Since stiffness is somewhat correlated with hardness and could be obtained from the same experiments, a model was trained without this feature and retained moderate predictive capabilities (*R* ^2^ = 0.46). While we were unable to obtain useful predictions from models trained only on molecular descriptors, we do show that it is possible to predict hardness with good accuracy using relatively easily obtainable experimental data: surface roughness, obtained from white light interferometry, and glass transition temperature, obtained from differential scanning calorimetry. This suggests that this approach could be useful in “bottom-up” strategies in materials design. By integrating experimental data with molecular descriptors, this approach offers a cost-effective pathway to accelerate the development of thermoplastic polymers with tailored mechanical properties. Future work should focus on addressing challenges associated with low-hardness polymers (such as PSU and PTFE) to further enhance predictive capabilities.

## Supplementary Material





## Data Availability

All data supporting this study are provided in the Supporting Information accompanying this paper.

## Notes

Additional experimental details and computational analysis, including the Python code used (PDF)
Hardness data (excel file) and python code (ZIP)

## Abbreviations

GPa: gigapascal
SMILES: simplified molecular input line entry system
*T*_g_: glass transition temperature
*T*_m_: melting temperature
MPa: megapascal
