# Supplementary material for: A Gradient Boosting-Based Machine Learning Model for the Prediction of the Hardness of Thermoplastic Polymers
Source: ACS Omega. 2026 Oct 21;11(43):67034–40. doi: 10.1021/acsomega.6c07307 (PMC13737836; doi:10.1021/acsomega.6c07307)
Supplement: Supplementary file 1 [file ao6c07307_si_001.pdf]

Supporting Information:

A gradient boosting-based machine learning model for the prediction of hardness of thermoplastic polymers

Jose Sena,[a,b,c] Jonny J. Blaker,[b*] Sam Hay[a*]

[a] *Department of Chemistry and Manchester Institute of Biotechnology, The University of Manchester, Manchester M1 7DN, United Kingdom.*

[b] *Department of Materials and Henry Royce Institute, The University of Manchester, Manchester M13 9PL, United Kingdom.*

[c] *Department of Chemistry, Autonomous University of Santo Domingo, Santo Domingo 10103, Dominican Republic.*

* jonny.blaker@manchester.ac.uk
* sam.hay@manchester.ac.uk

Hardness data (excel file) and python code are also provided in an accompanying zip file (Sena_Supplementary_Files).

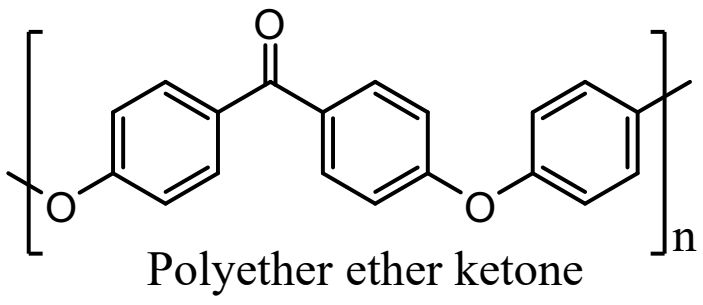

Polyether ether ketone
(PEEK)

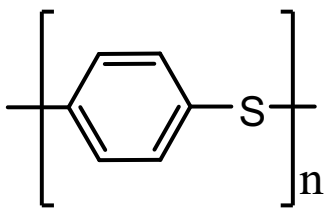

Polyphenylene sulfide
(PPS)

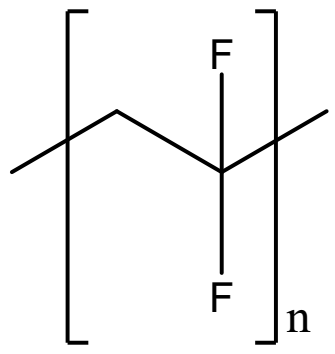

Polyvinylidene fluoride
(PVDF)

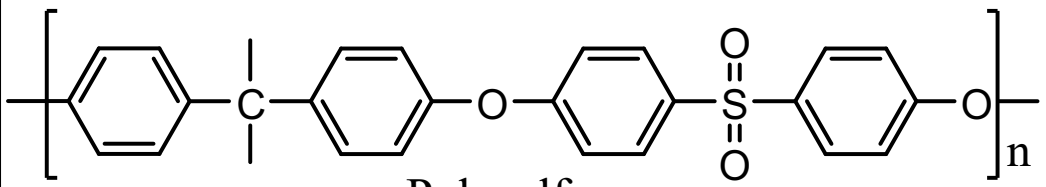

Polysulfone
(PSU)

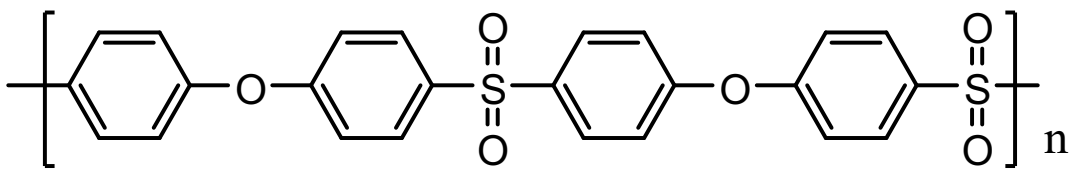

Polyethersulfone
(PES)

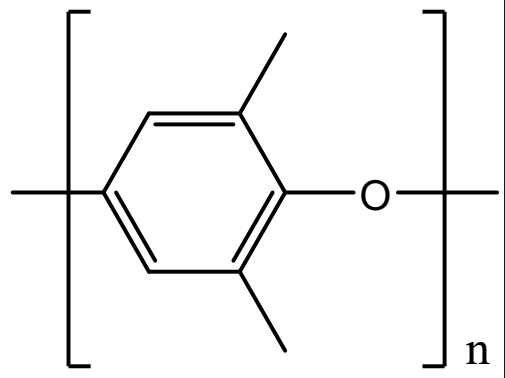

Poly(p-phenylene oxide)
(PPO)

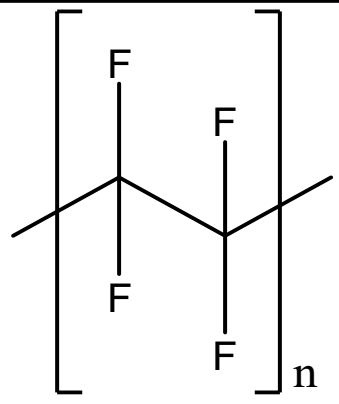

Polytetrafluoroethylene
(PTFE)

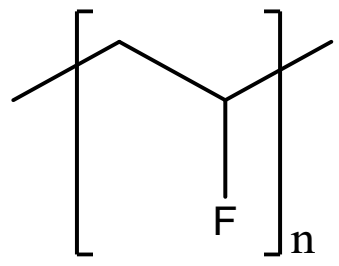

Polyvinyl fluoride
(PVF)

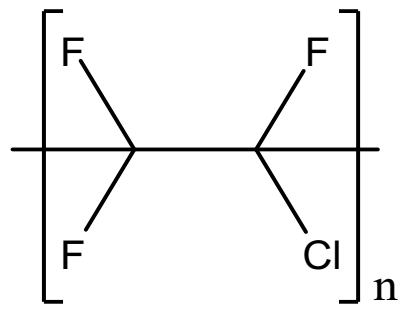

Polychlorotrifluoroethylene
(PCTFE)

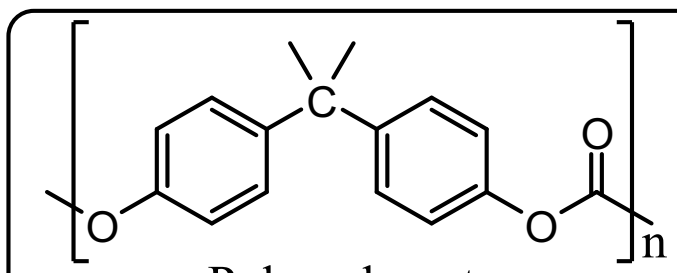

Polycarbonate
(PC)

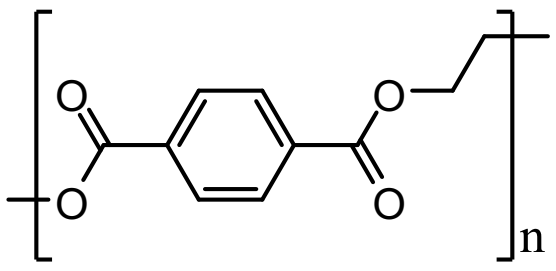

Polyethylene terephthalate
(PET)

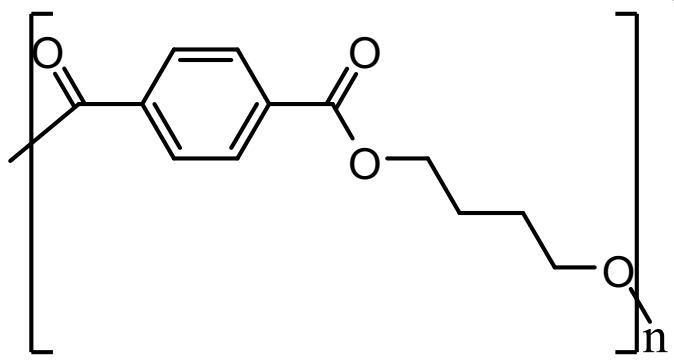

Polybutylene terephthalate
(PBT)

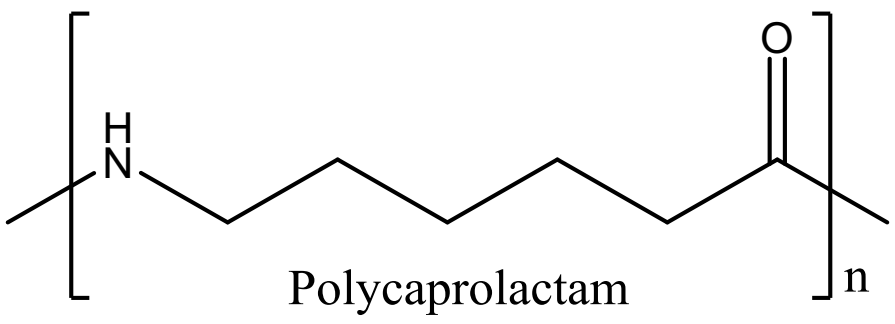

Polycaprolactam
(Nylon 6)

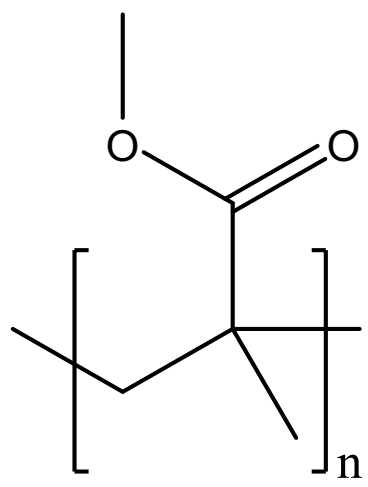

Poly(methyl methacrylate)
(PMMA)

Continued next page.

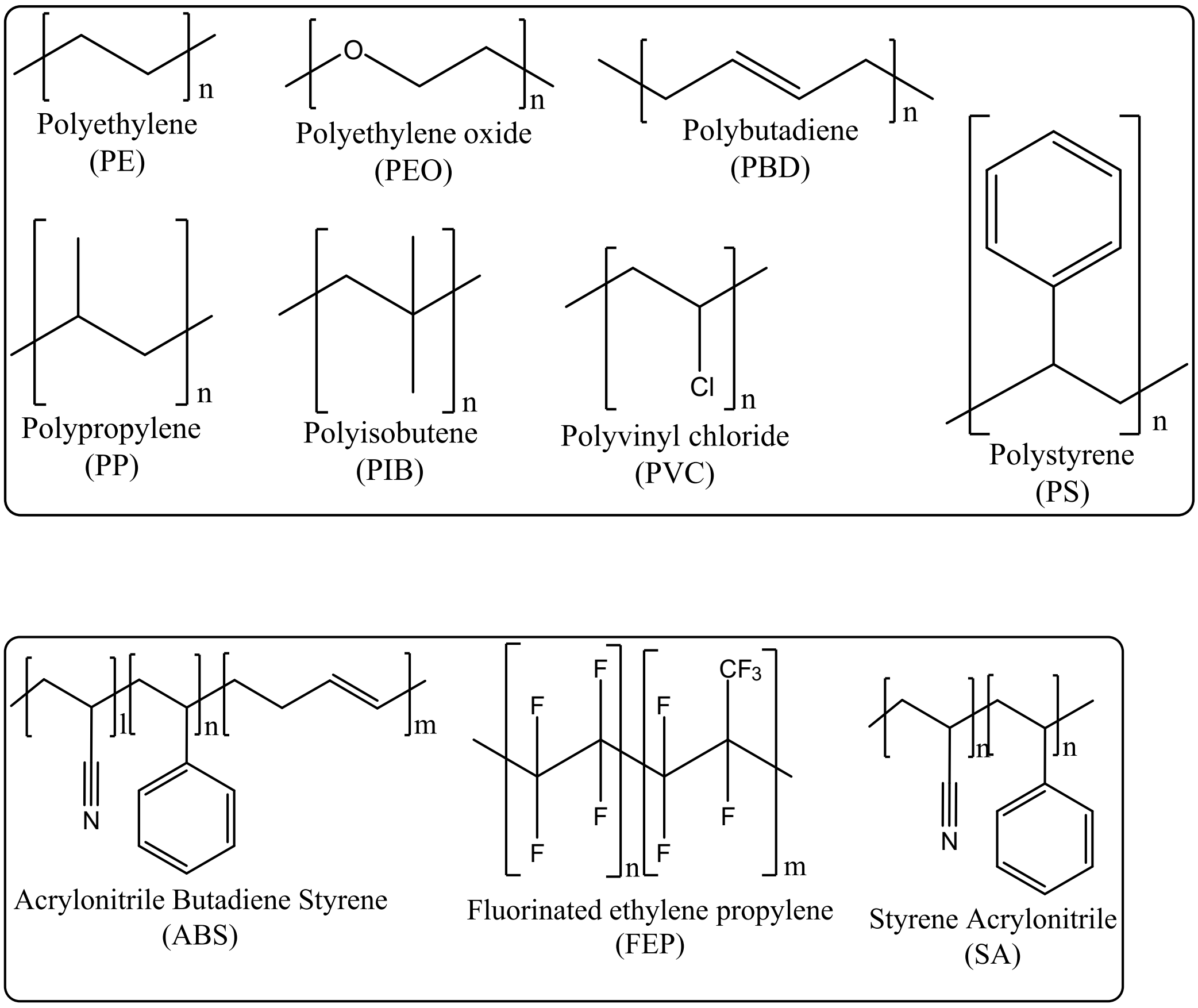

Figure S1. The molecular structure of polymers used in this study.

Table S1. Experimental data obtained from in-house measurements.

Polymer	Stiffness (μN/nm)	Young's Modulus (GPa)	T_g (°C)	T_m (°C)	Crystallinity percentage	Surface roughness (nm)	Hardness (GPa)
PEEK	34.25±0.44	4.60±0.22	155.9	341.7	25.83	52	0.230±0.022
PPS	22.05±3.28	2.48±0.39	86.8	282.9	56.89	318	0.160±0.010
PSU	11.47±0.43	0.63±0.04	188.1	--	0.00	485	0.038±0.002
PC	24.68±0.36	3.15±0.04	148.9	--	0.00	66	0.208±0.006
PE	31.31±3.61	2.36±0.48	-105.0	135.1	51.79	243	0.073±0.017
PP	25.40±0.70	2.23±0.15	-8.5	164.5	38.27	89	0.099±0.012
PET	30.22±0.64	3.38±0.16	82.6	247.9	22.31	35	0.160±0.014
PMMA	36.52±1.11	4.97±0.24	115.8	--	0.00	33	0.238±0.025
ABS	27.52±1.73	3.22±0.37	109.7	--	0.00	96	0.175±0.027
FEP	13.53±0.40	0.87±0.05	141.5	258.1	11.25	140	0.052±0.004
NYLON6	21.05±0.28	1.93±0.04	52.6	219.9	32.22	60	0.107±0.003
PBD	25.27	2.71	-106.0	--	0.00	150	0.140
PCTFE	20.41±0.08	2.01±0.03	54.0	212.9	26.42	74	0.124±0.003
PIB	24.90	2.66	-65.0	--	0.00	259	0.139
PS	33.26±0.36	4.55±0.09	103.7	--	0.00	33	0.239±0.009
PTFE	22.18±1.33	1.37±0.11	120.5	330.2	23.35	416	0.048±0.004
PVOH	25.25	2.70	85.0	240.0	24.39	11	0.139
PVC	33.85±0.12	4.68±0.06	75.2	--	0.00	43	0.244±0.006
PVF	25.70	2.80	-20.0	195.0	15.84	40	0.145
PVDF	29.77±1.60	2.81±0.36	-33.5	171.7	39.59	62	0.117±0.034
PES	27.30±0.61	3.11±0.10	225.9	--	0.00	71	0.165±0.010
PPO	25.96±2.13	2.81±0.54	143.7	--	0.00	713	0.144±0.019
PBT	24.00±0.45	2.40±0.12	53.1	219.5	10.99	818.00	0.128±0.011

PBD, PIB, PVOH, and PVF data were collected from the literature[19-21].

Table S2. Molecular descriptors obtained from the trimers SMILES strings using RDKit.

Polymer	SP3 hybridization	SP2 hybridization	Non-H atoms	Trimer molecular weight	Number of Hydrogen atoms	Number of Valence electrons
PEEK	6	33	72	955	54	360
PPS	3	18	21	331	18	108
PSU	12	78	90	1256	67	456
PC	9	48	57	769	48	294
PE	6	0	6	86	14	38
PP	10	0	10	142	22	62
PET	6	36	42	583	30	222
PMMA	12	9	21	306	30	126
ABS	1	12	45	586	39	222
FEP	17	0	51	864	0	312
NYLON6	6	2	24	346	39	144
PBD	2	2	12	168	24	72

PCTFE	6	0	18	355	6	114
PIB	4	0	12	174	30	78
PS	0	8	24	312	24	120
PTFE	6	0	18	306	6	114
PVOH	3	0	9	138	18	60
PVC	3	0	9	194	15	60
PVF	3	0	9	144	15	60
PVDF	4	0	12	198	12	78
PES	2	30	96	1400	55	486
PPO	0	7	21	282	18	108
PBT	5	11	48	667	42	258

Table S3. Initial hardness prediction for unoptimized models.[a]

Polymer	Index	Experimental	Decision Tree	Random Forest	XGBoost
PEEK	0	0.230	0.221	0.239	0.197
PPS	1	0.160	0.060	0.124	0.121
PSU	2	0.038	0.183	0.208	0.169
PC	3	0.208	0.103	0.124	0.126
PE	4	0.073	0.119	0.117	0.129
PP	5	0.099	0.128	0.107	0.128
PET	6	0.160	0.157	0.160	0.150
PMMA	7	0.238	0.244	0.230	0.215
ABS	9	0.175	0.096	0.145	0.143
FEP	10	0.052	0.062	0.124	0.105
NYLON6	11	0.107	0.155	0.124	0.181
PBD	12	0.140	0.139	0.139	0.139
PCTFE	13	0.124	0.087	0.073	0.093
PIB	14	0.139	0.135	0.117	0.127
PS	15	0.239	0.165	0.238	0.219
PTFE	16	0.048	0.113	0.124	0.120
PVOH	18	0.139	0.144	0.145	0.149
PVC	19	0.244	0.216	0.239	0.201
PVF	20	0.145	0.133	0.139	0.133
PVDF	21	0.117	0.111	0.145	0.128
PES	22	0.165	0.176	0.175	0.175
PPO	23	0.144	0.148	0.128	0.135
PBT	25	0.128	0.150	0.175	0.128

[a] These data are also plotted in **Figure S2**.

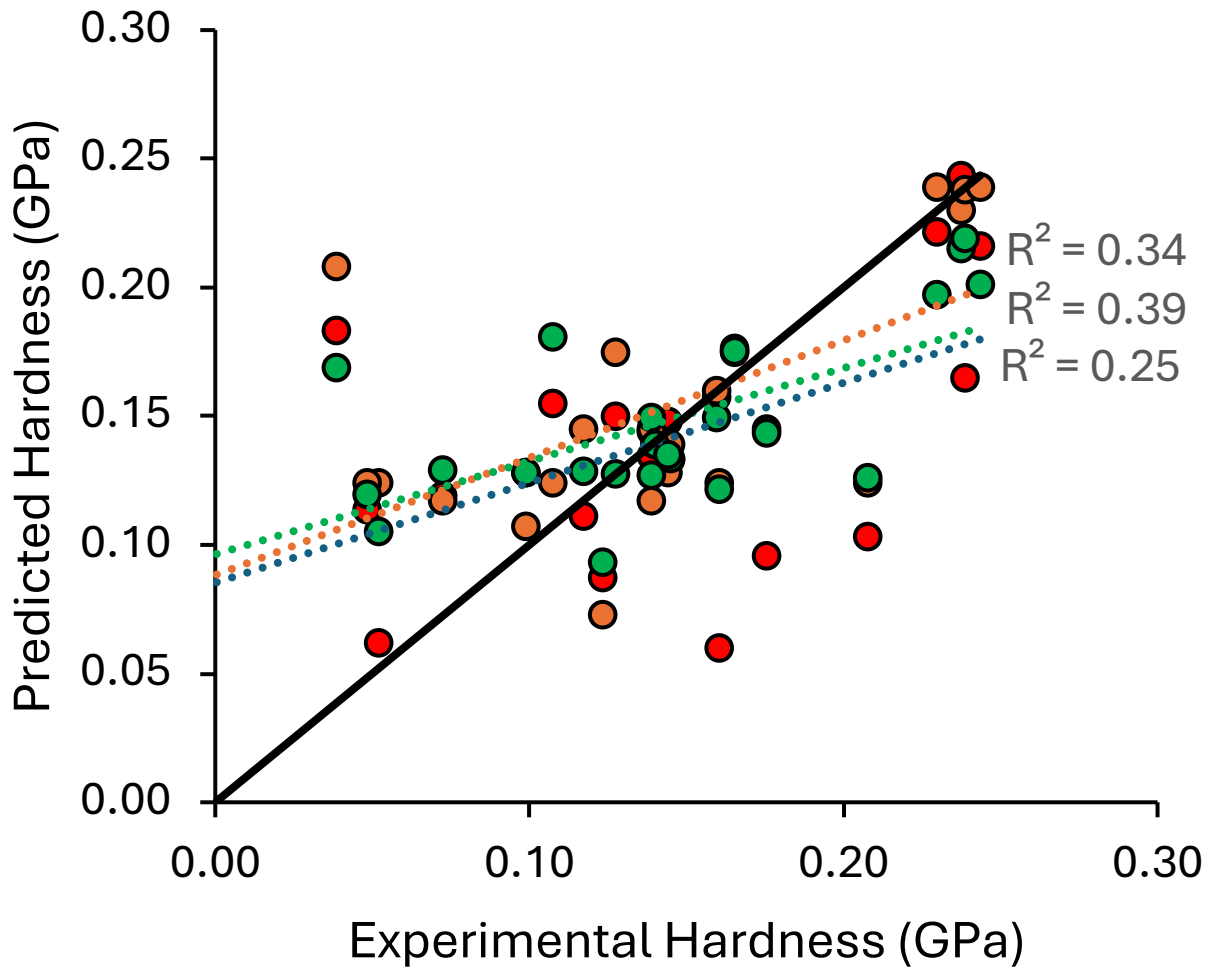

Figure S2. Predicted *vs.* experimental hardness from **Table S2.** The dashed orange line represents the model using random forest algorithm, the dashed green line is the prediction using a XGBoost, and the dashed blue line is the prediction using a decision tree algorithm.

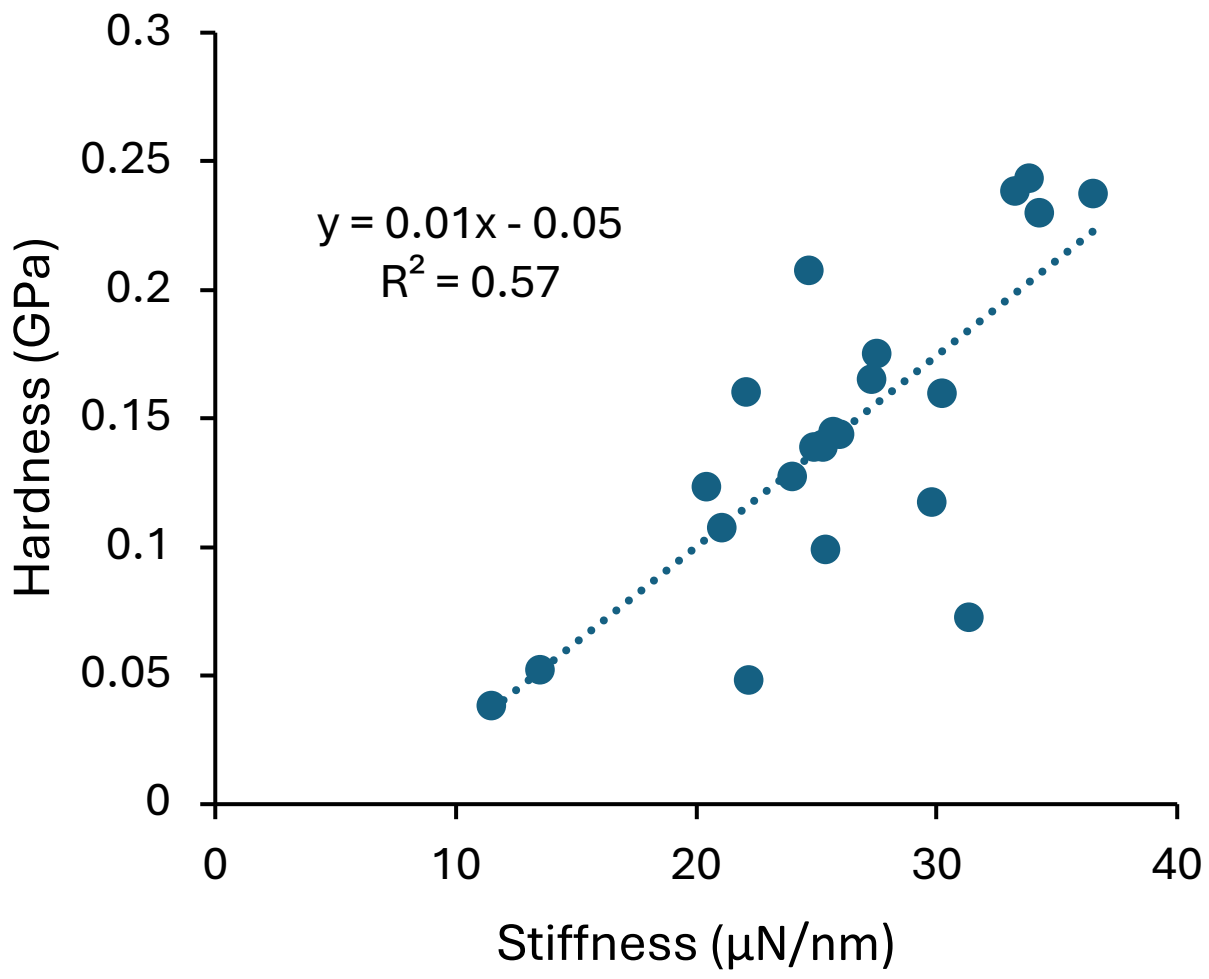

Figure S3. Correlation between hardness and stiffness.

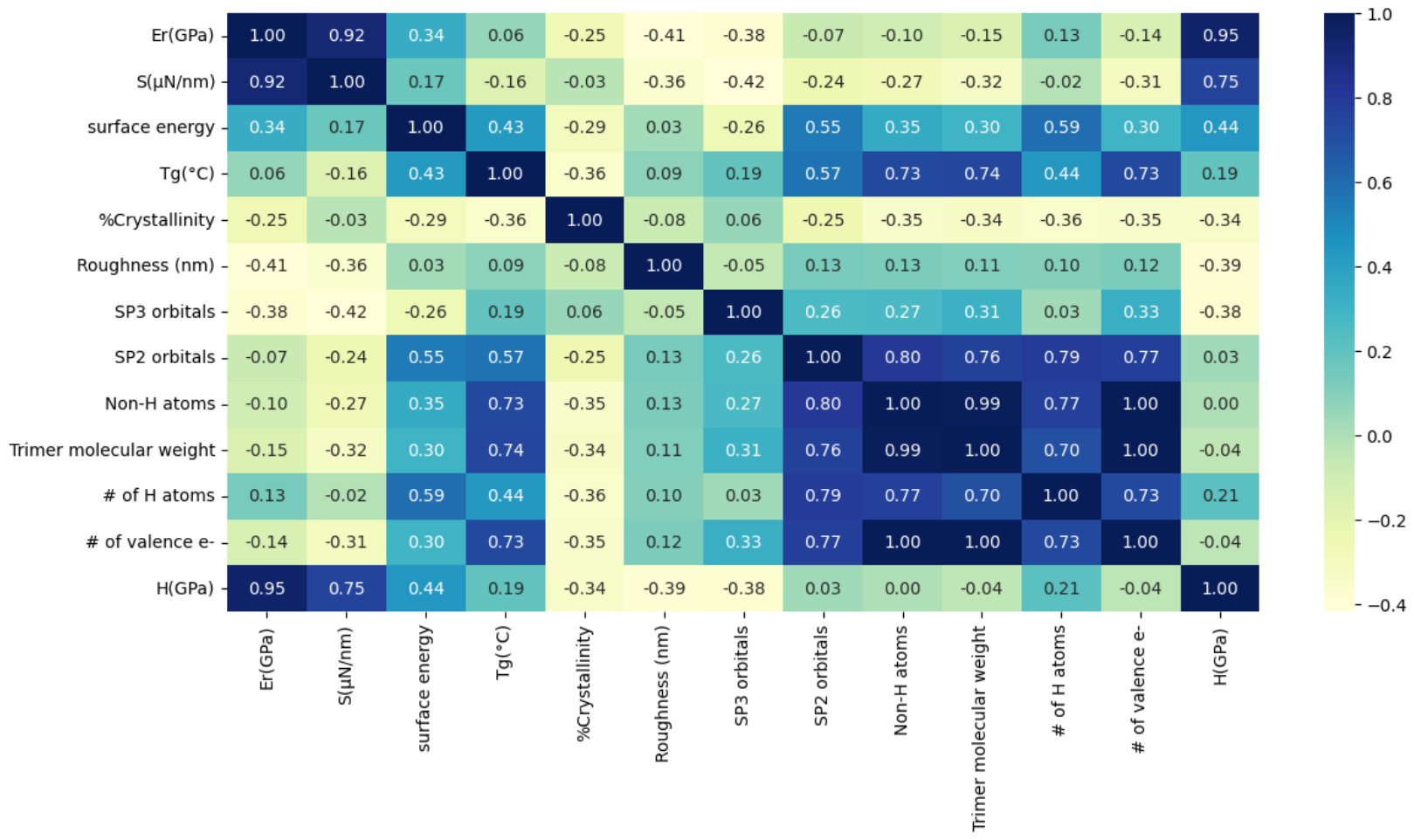

Figure S4. Heatmap showing correlation between the different features and the output used in this study. The heatmap shows Pearson correlation. Two features (Young's modulus and Stiffness) show linear correlation with the output (hardness). Since Young's modulus is heavily correlated, it was excluded from the study from the beginning.

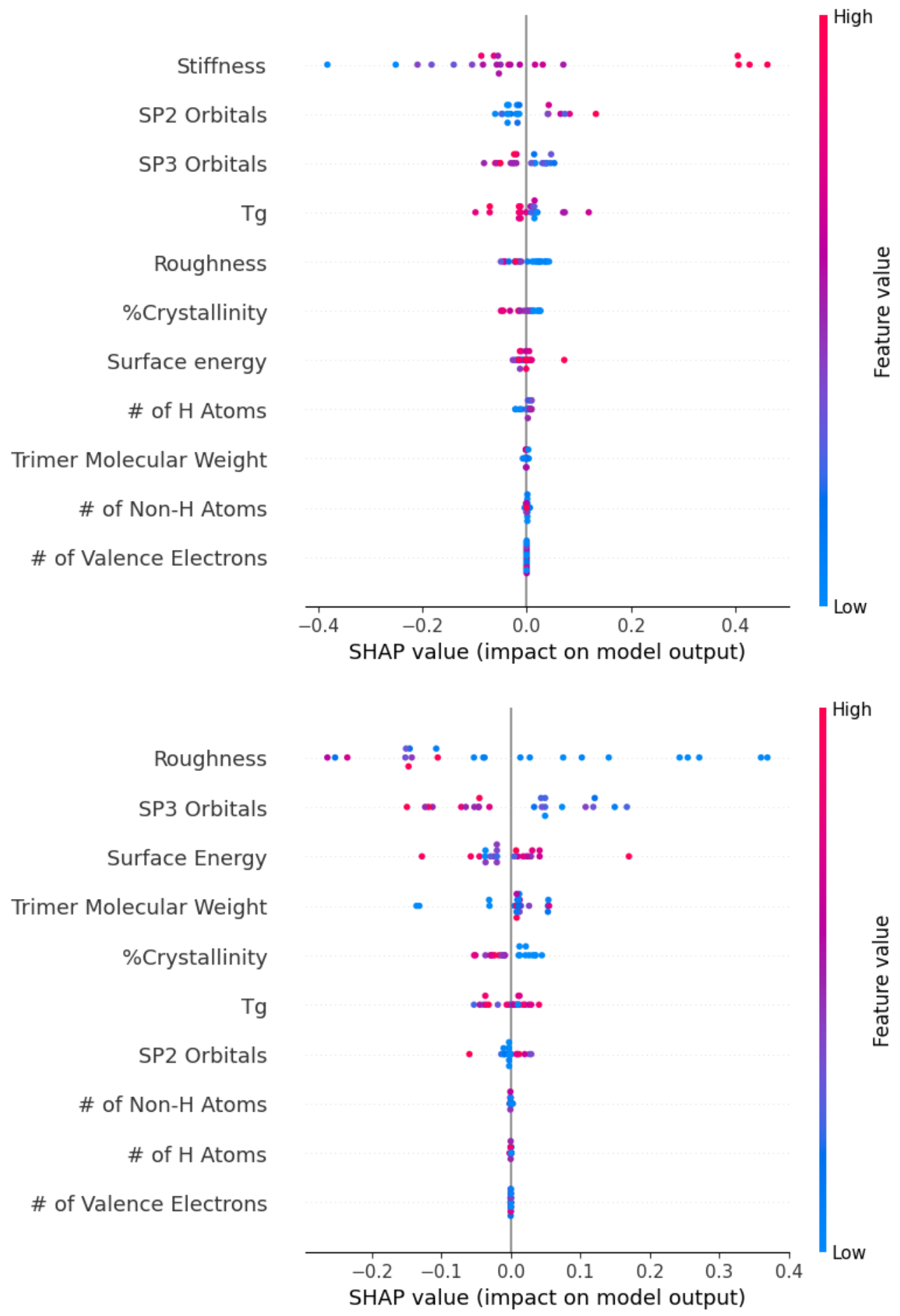

Figure S5. Relative features importance using Shapley Additive Explanations (SHAP) values. *Top*, for the optimized XGBoost model using all features in Table 2. *Bottom*, for the optimized XGBoost model trained without the stiffness feature.

Table S4. Hyperparameter optimization for the XGBoost algorithm.

Polymer	Index	n_estimators	max_depth	Learning rate
PEEK	0	100	6	0.4
PPS	1	10	3	0.1
PSU	2	10	1	0.1
PC	3	100	1	0.7
PE	4	100	5	0.2
PP	5	100	2	0.3
PET	6	100	2	0.3
PMMA	7	100	4	0.3
ABS	9	100	1	0.3
FEP	10	100	6	0.3
NYLON6	11	100	4	0.5
PBD	12	100	4	0.5
PCTFE	13	100	6	0.5
PIB	14	100	5	0.5
PS	15	100	4	0.5
PTFE	16	100	6	0.5
PVOH	18	100	6	0.3
PVC	19	100	4	0.4
PVF	20	100	6	0.5
PVDF	21	100	6	0.5
PES	22	100	6	0.4
PPO	23	100	6	0.3
PBT	25	100	3	0.3
	**Average**	92	4	0.4

The average of the hyperparamentes shows that the model does not suffer from overfitting as the step size between each features weight update (learning rate) is like the default value and the model is not shallow or overcomplicated either. The subsample size for optimization was 0.9, this means that 10% of the dataset was holdout for model verification purpose. This value was selected due to the small size of the dataset.

Table S5. Hyperparameter optimization for the ML model without the stiffness feature.

Polymer	Index	n_estimators	max_depth	Learning rate
PEEK	0	100	4	0.3
PPS	1	100	6	0.3
PSU	2	100	6	0.3
PC	3	10	2	0.3
PE	4	100	2	0.3
PP	5	100	2	0.2
PET	6	10	1	0.1
PMMA	7	100	6	0.3
ABS	9	100	5	0.1
FEP	10	100	2	0.7
NYLON6	11	100	2	0.2
PBD	12	100	3	0.2
PCTFE	13	100	3	0.2
PIB	14	100	4	0.4
PS	15	100	6	0.3
PTFE	16	100	6	0.3
PVOH	18	100	1	0.6
PVC	19	100	1	0.2
PVF	20	100	1	0.2
PVDF	21	100	1	0.2
PES	22	10	5	0.2
PPO	23	10	5	0.2
PBT	25	10	1	0.1
	**Average**	80	3	0.3

The average of the hyperparameters shows that the model does not suffer from overfitting as the step size between each features weight update (learning rate) is like the default value and the model is not shallow or overcomplicated either. The subsample size for optimization was 0.9, this means that 10% of the dataset was holdout for model verification purpose. This value was selected due to the small size of the dataset.

Table S6. Nested Cross-validation optimization for the ML model using all features. The outer CV k value was set to 5, while the inner CV value was set to 4. Code can be found in Supplementary Material 3.

K-fold	Learning rate	Max depth	n_estimators	RMSE	R^2	Subsample
1	0.3	2	50	0.04	0.74	1.0
2	0.7	1	100	0.08	-2.44	0.6
3	0.3	2	100	0.07	-1.38	0.8
4	0.6	5	50	0.02	0.80	0.6
5	0.5	2	10	0.04	0.32	0.6

Each iteration of the outer CV reports the estimated performance of the best performing model using a 4-fold CV and the hyperparameters found to perform the best. The RMSE and R^2 values are the prediction of the model using the holdout dataset.

Table S7. Nested Cross-validation optimization for the ML model using all features but Stiffness. The outer CV k value was set to 5, while the inner CV value was set to 4. Code can be found in Supplementary Material 3.

K-fold	Learning rate	Max depth	n_estimators	RMSE	R^2	Subsample
1	0.1	4	10	0.08	0.14	1.0
2	0.1	1	50	0.06	-1.28	0.8
3	0.3	5	50	0.08	-1.99	1.0
4	0.2	5	100	0.03	0.46	0.6
5	0.4	2	10	0.07	-0.61	0.6

Each iteration of the outer CV reports the estimated performance of the best performing model using a 4-fold CV and the hyperparameters found to perform the best. The RMSE and R^2 values are the prediction of the model using the holdout dataset.

Table S8. Output of ML model. The predicted values were computed using experimental and computational features, these values are also presented in **Figure 1**.

Polymer	Index	Experimental values	Predicted values	Difference (exp. – pred.)	%e
PEEK	0	0.230	0.221	0.0085	3.72
PPS	1	0.160	0.111	0.0496	30.96
PSU	2	0.038	0.153	-0.1140	296.21
PC	3	0.208	0.153	0.0548	26.40
PE	4	0.073	0.110	-0.0376	51.97
PP	5	0.099	0.116	-0.0169	17.14
PET	6	0.160	0.160	-0.0004	0.29
PMMA	7	0.238	0.240	-0.0018	0.76
ABS	9	0.175	0.124	0.0513	29.30
FEP	10	0.052	0.051	0.0009	1.90
NYLON6	11	0.107	0.155	-0.0477	44.46
PBD	12	0.140	0.139	0.0006	0.43
PCTFE	13	0.124	0.087	0.0363	29.39
PIB	14	0.139	0.135	0.0044	3.20
PS	15	0.239	0.165	0.0735	30.81
PTFE	16	0.048	0.114	-0.0650	134.43
PVOH	18	0.139	0.136	0.0025	1.86
PVC	19	0.244	0.221	0.0227	9.32
PVF	20	0.145	0.139	0.0059	4.10
PVDF	21	0.117	0.111	0.0058	5.02
PES	22	0.165	0.176	-0.0106	6.47
PPO	23	0.144	0.148	-0.0037	2.62
PBT	25	0.128	0.132	-0.0045	3.56

Table S9. Values for the William's plot shown in **Figure 4**. This graph employs three main parameters: leverage threshold, standardized residuals and response outlier. The leverage threshold are the lines above and below 3 standard deviations. The standardized residuals are calculated using the absolute error between the experimental and predicted values. The response outlier are those values that fall outside the 3 standard deviations lines.

Polymer	Leverage	Residual	Standardized Residual	High Leverage	Response Outlier
PEEK	0.4703	0.0086	0.2119	No	No
PPS	0.5096	0.0496	1.2786	No	No
PSU	0.7664	-0.1140	-4.2533	No	Yes
PC	0.3195	0.0548	1.1980	No	No
PE	0.5002	-0.0377	-0.9616	No	No
PP	0.5226	-0.0169	-0.4422	No	No
PET	0.4867	-0.0005	-0.0118	No	No
PMMA	0.6652	-0.0018	-0.0564	No	No
ABS	0.4451	0.0514	1.2444	No	No
FEP	0.8510	0.0010	0.0463	No	No
NYLON6	0.6826	-0.0478	-1.5289	No	No
PBD	0.4313	0.0006	0.0144	No	No
PCTFE	0.2887	0.0363	0.7765	No	No
PIB	0.3237	0.0044	0.0975	No	No
PS	0.2816	0.0735	1.5649	No	No
PTFE	0.2610	-0.0651	-1.3652	No	No
PVOH	0.2913	0.0026	0.0555	No	No
PVC	0.5123	0.0227	0.5862	No	No
PVF	0.1659	0.0059	0.1175	No	No
PVDF	0.2692	0.0059	0.1242	No	No
PES	0.7525	-0.0107	-0.3875	No	No
PPO	0.5577	-0.0038	-0.1025	No	No
PBT	0.6453	-0.0045	-0.1375	No	No

Table S10. Feature total gain computed using the XGBoost built-in utility.

Feature index	Feature name	Total Gain
F2	Stiffness	0.293
F3	Surface Energy	0.050
F4	Glass transition Temp.	0.145
F5	%Crystallinity	0.064
F6	Surface Roughness	0.054
F7	SP3 orbitals	0.135
F8	SP2 orbitals	0.142
F9	Non-H atoms	0.004
F10	Trimer molecular weight	0.007
F11	# of Hydrogen atoms	0.106
F12	# of valence electrons	0.000

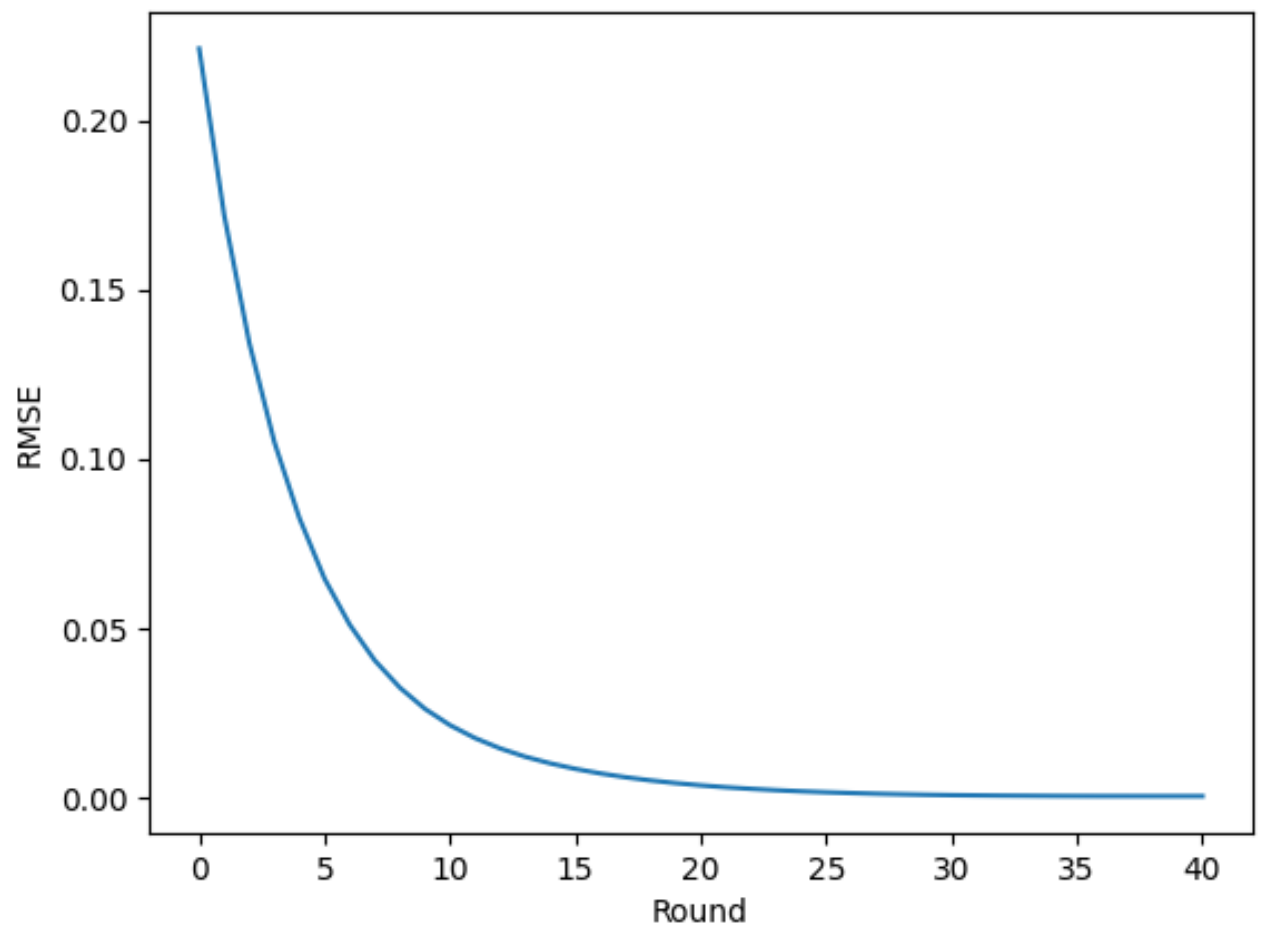

Figure S6. Cross validation using XGBoost. The number of iterations (rounds) was 41, with 10-fold cross-validations. The learning rate was 0.3, like the average for the optimized model presented in **Figure 1**, and the optimized metric for this figure is RMSE.

Table S11. Output of ML model without the stiffness feature, these values are presented in **Figure 3**.

Polymer	Index	Experimental values	Predicted values	Difference (exp. – pred.)	%e
PEEK	0	0.230	0.144	0.086	37.33
PPS	1	0.160	0.133	0.027	17.00
PSU	2	0.038	0.168	-0.129	336.20
PC	3	0.208	0.124	0.083	40.21
PE	4	0.073	0.095	-0.023	31.05
PP	5	0.099	0.109	-0.010	10.05
PET	6	0.160	0.170	-0.010	6.53
PMMA	7	0.238	0.228	0.010	4.07
ABS	9	0.175	0.152	0.024	13.45
FEP	10	0.052	0.066	-0.014	25.94
NYLON6	11	0.107	0.198	-0.091	84.50
PBD	12	0.140	0.140	0.000	0.14
PCTFE	13	0.124	0.124	-0.001	0.46
PIB	14	0.139	0.133	0.006	4.09
PS	15	0.239	0.238	0.001	0.28
PTFE	16	0.048	0.079	-0.030	62.57
PVOH	18	0.139	0.199	-0.060	43.31
PVC	19	0.244	0.186	0.057	23.47
PVF	20	0.145	0.144	0.001	0.48
PVDF	21	0.117	0.119	-0.002	1.52
PES	22	0.165	0.180	-0.015	9.22
PPO	23	0.144	0.143	0.001	0.90
PBT	25	0.128	0.133	-0.005	3.91

Table S12. Feature total gain computed using the XGBoost built-in utility for the model trained without the stiffness feature.

Feature index	Feature name	Total Gain
F3	Surface Energy	0.066
F4	Glass transition Temp.	0.067
F5	%Crystallinity	0.072
F6	Surface Roughness	0.308
F7	SP3 orbitals	0.284
F8	SP2 orbitals	0.032
F9	Non-H atoms	0.003
F10	Trimer molecular weight	0.112
F11	# of Hydrogen atoms	0.054
F12	# of valence electrons	0.001

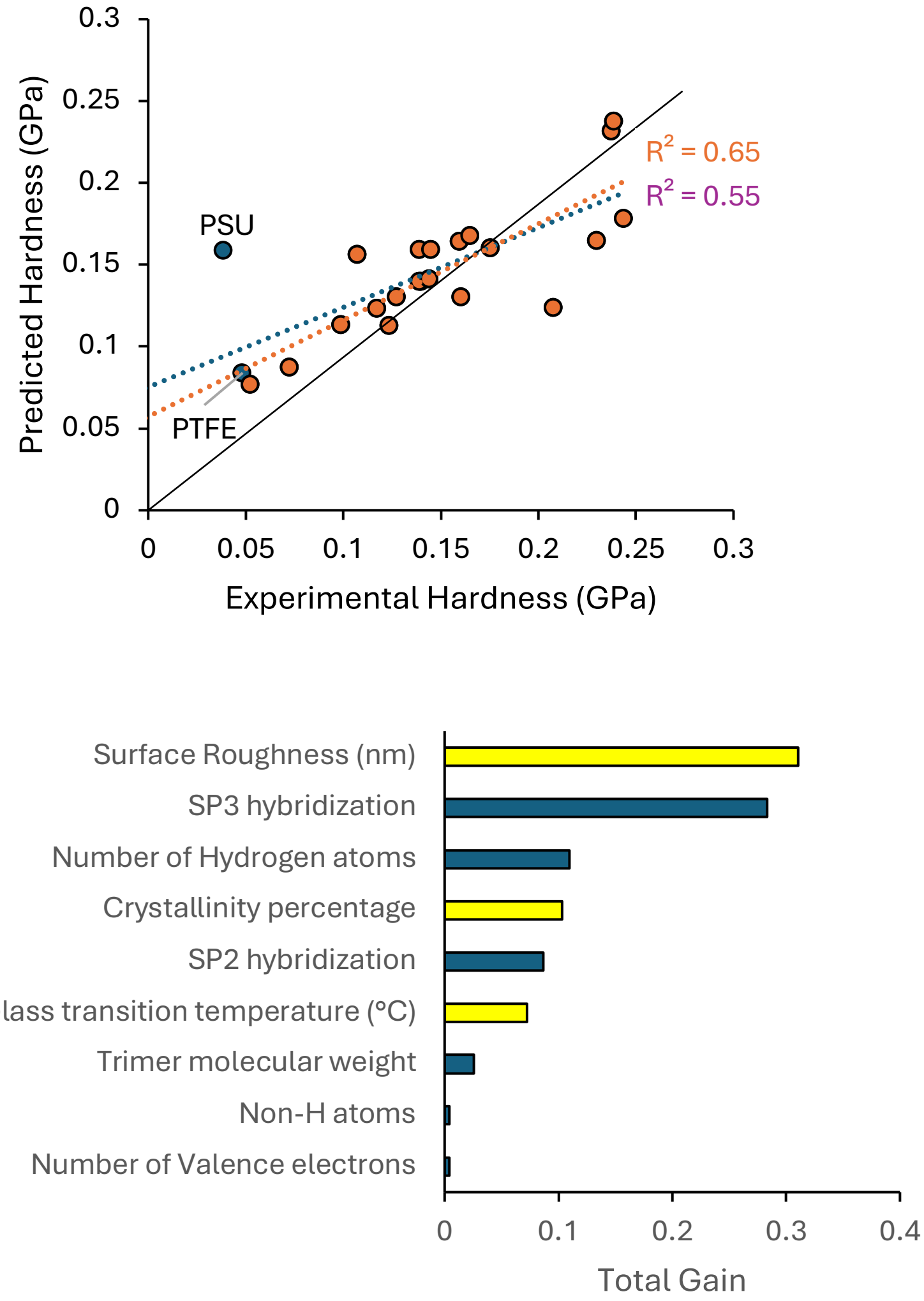

Figure S7. Output of the XGBoost ML model trained without the stiffness and surface energy features. *Top,* the predicted *vs*. experimental hardness. The dashed purple line represents the prediction of all 23 polymers; the dashed orange line is the prediction excluding two polymers (PSU and PTFE). *Bottom,* Relative feature importance, with experimental features presented in yellow and molecular descriptors obtained with RDKit are in blue. Total gain values are given in **Table S14**.

Table S13. Output of ML model without the stiffness and surface energy features, these values are presented in **Figure S7**.

Polymer	Index	Experimental values	Predicted values	Difference (exp. – pred.)	%e
PEEK	0	0.230	0.1647	0.0652	28.37
PPS	1	0.160	0.1303	0.0301	18.77
PSU	2	0.038	0.1588	-0.1203	312.53
PC	3	0.208	0.1238	0.0838	40.37
PE	4	0.073	0.0877	-0.0152	20.90
PP	5	0.099	0.1133	-0.0144	14.58
PET	6	0.160	0.1646	-0.0050	3.12
PMMA	7	0.238	0.2317	0.0060	2.52
ABS	9	0.175	0.1602	0.0152	8.68
FEP	10	0.052	0.0770	-0.0248	47.61
NYLON6	11	0.107	0.1566	-0.0492	45.79
PBD	12	0.140	0.1401	-0.0001	0.08
PCTFE	13	0.124	0.1129	0.0106	8.60
PIB	14	0.139	0.1400	-0.0010	0.74
PS	15	0.239	0.2380	0.0007	0.27
PTFE	16	0.048	0.0839	-0.0355	73.40
PVOH	18	0.139	0.1593	-0.0203	14.58
PVC	19	0.244	0.1783	0.0653	26.80
PVF	20	0.145	0.1594	-0.0144	9.91
PVDF	21	0.117	0.1232	-0.0059	5.04
PES	22	0.165	0.1681	-0.0029	1.78
PPO	23	0.144	0.1413	0.0027	1.89
PBT	25	0.128	0.1306	-0.0031	2.40

Table S14. Feature total gain computed using the XGBoost built-in utility for the model trained without the stiffness and surface energy features.

Feature index	Feature name	Total Gain
F3	Glass transition Temp.	0.072
F4	%Crystallinity	0.103
F5	Surface Roughness	0.311
F6	SP3 orbitals	0.283
F7	SP2 orbitals	0.087
F8	Non-H atoms	0.004
F9	Trimer molecular weight	0.026
F10	# of Hydrogen atoms	0.109
F11	# of valence electrons	0.004

Table S15. Features importance for models separated by morphology.

Feature name	Semi-crystalline	Amorphous
# of valence electrons	0.000	0.000
# of Hydrogen atoms	0.094	0.000
Trimer molecular weight	0.001	0.000
Non-H atoms	0.000	0.001
SP2 orbitals	0.441	0.000
SP3 orbitals	0.244	0.000
Surface Roughness	0.122	0.794
%Crystallinity	0.015	0.000
Glass transition temp.	0.065	0.011
Stiffness	0.018	0.194

Table S16. Relative features importance using other ML algorithms. These are the features importance values without stiffness.

	Feature rank				
Model, method	1st	2nd	3rd	4th	5th
XGBoost, total gain	Surface roughness	SP3 orbitals	# of H atoms	%Crystallinity	SP2 orbitals
XGBoost, SHAP	Surface roughness	SP3 orbitals	Surface energy	Trimer Molecular weight	%Crystallinity
XGBoost (unoptimized), total gain	SP3 orbitals	Trimer Molecular weight	Surface roughness	Surface energy	Glass Transition temperature
Decision Tree (unoptimized), Gini importance	Surface roughness	SP3 orbitals	SP2 orbitals	Trimer Molecular weight	Surface energy
Random forest (unoptimized), Gini importance	Surface roughness	SP3 orbitals	Surface energy	Glass Transition temperature	%Crystallinity

Total gain is a metric that quantifies how much a feature contributes to the overall performance of the model. It represents the sum of the gain across all splits in the tree ensemble where the feature was used. Meanwhile, Gini importance quantifies the importance of a feature by assessing how much the Gini impurity of a node decreases when that feature is used to split the node, Gini impurity is used to determine the best way to split nodes.

Table S17. Summary of R^2 metrics for the different ML models presented in this study. The models presented in this table where trained using XGBoost

Model	R^2	PSU and PTFE removed	PSU and NYLON6 removed
Unoptimized model, trained using all features	0.39	--	--
Optimized model, trained using all features	0.54	0.69	--
Optimized model, trained without stiffness	0.39	0.46	0.64
Optimized model, trained without surface energy but with stiffness	0.73	0.88	--
Optimized model, trained without stiffness and surface energy	0.55	0.65	--

Figure S8. Surface roughness measurements:

Polyether ether ketone (PEEK), Sa = 52nm

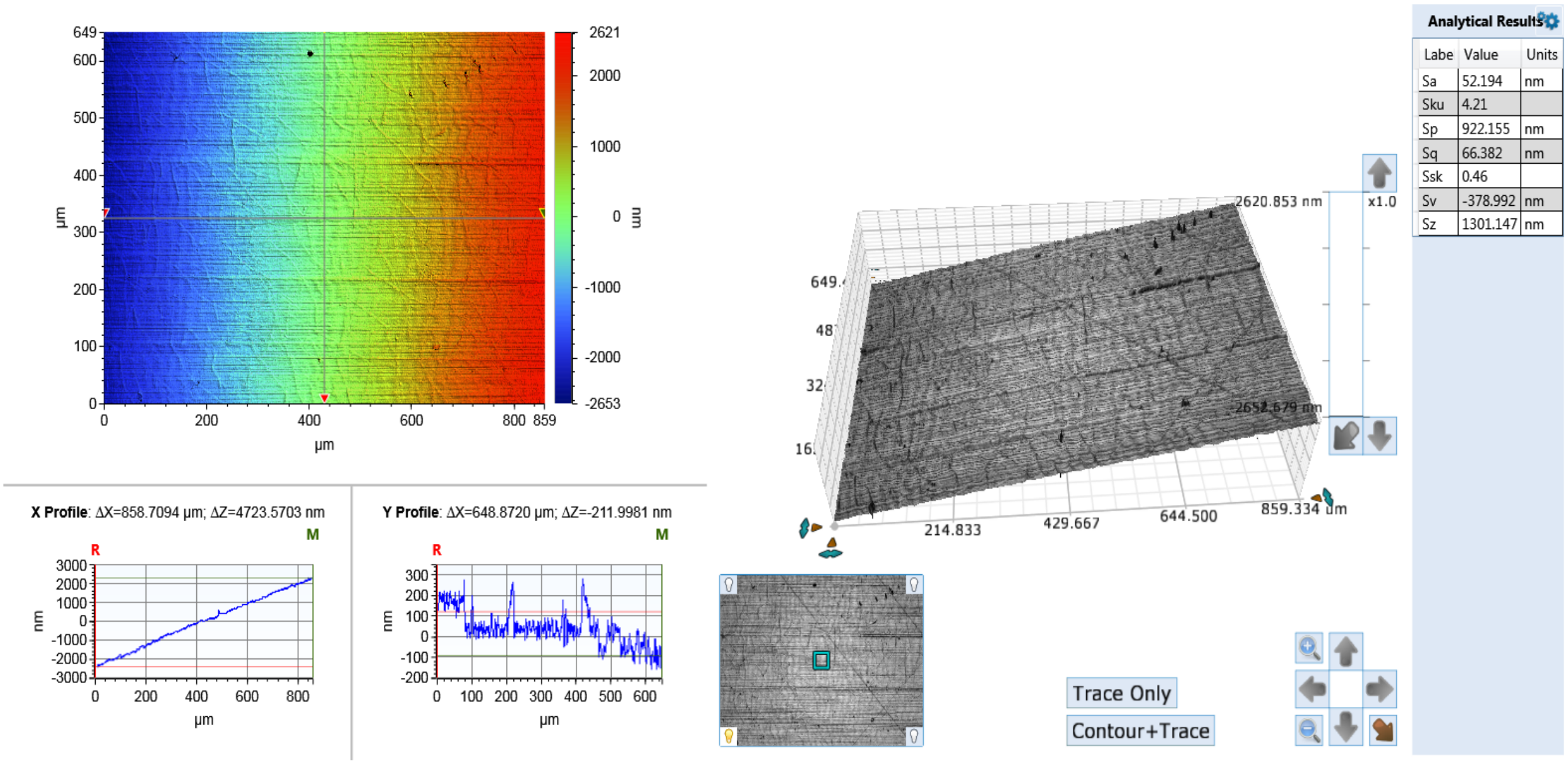

Polyphenylene sulfide (PPS), Sa = 318nm

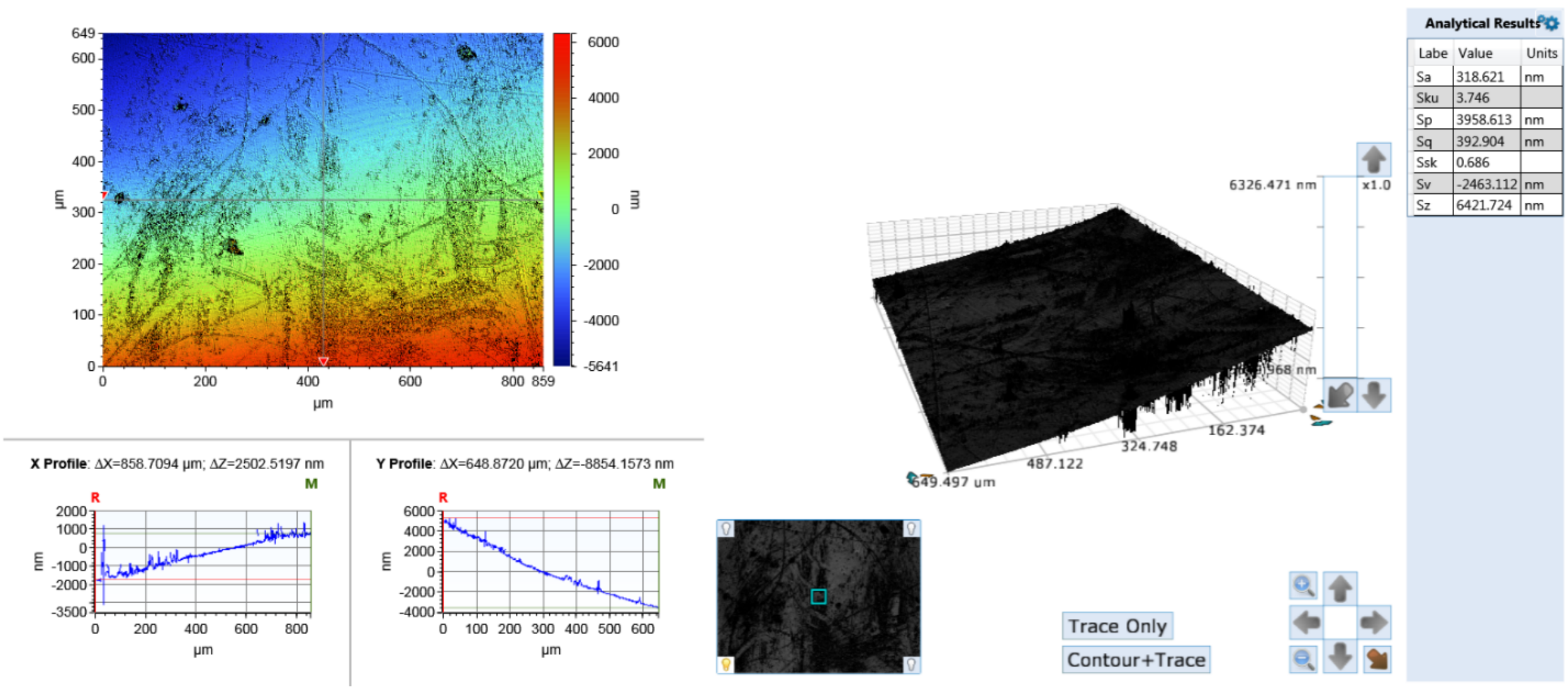

Polysulfone (PSU), Sa = 450nm

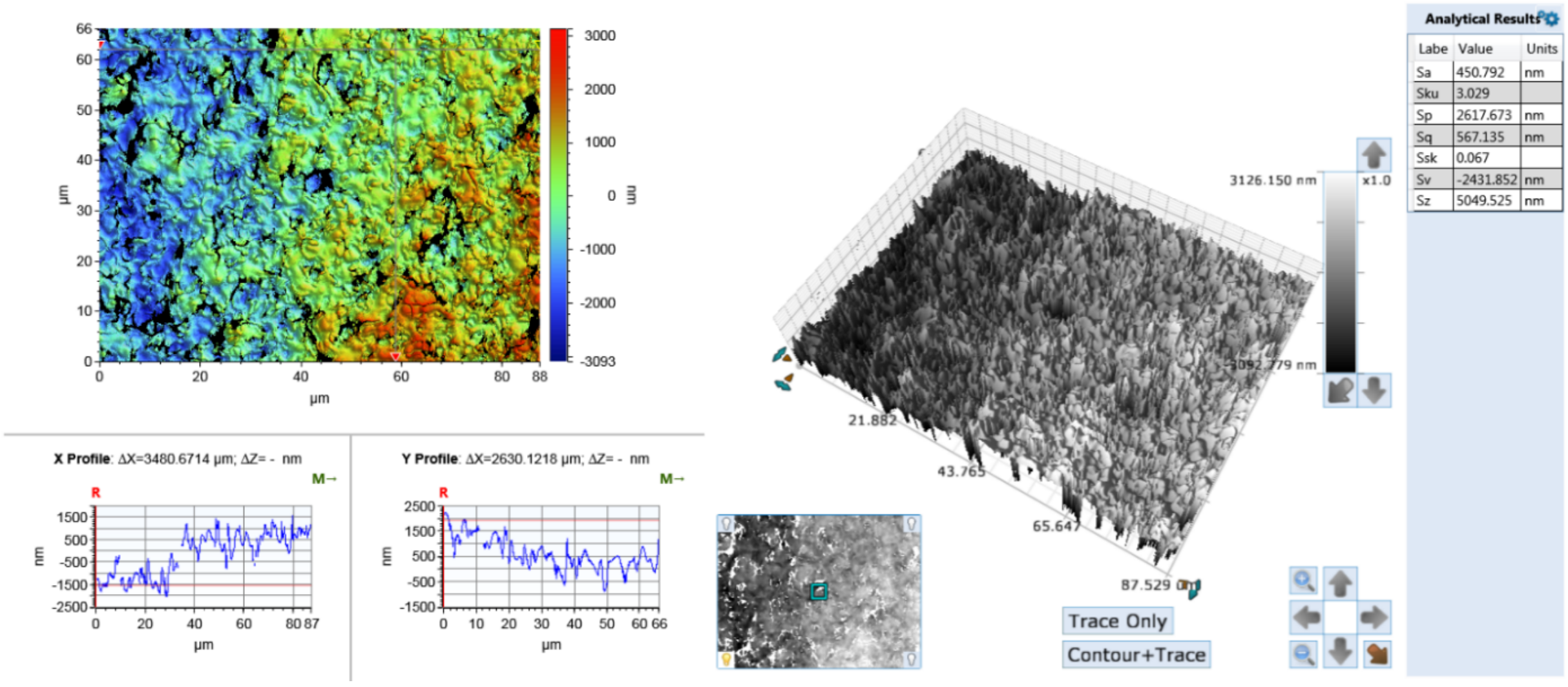

Polycarbonate (PC), Sa = 66nm

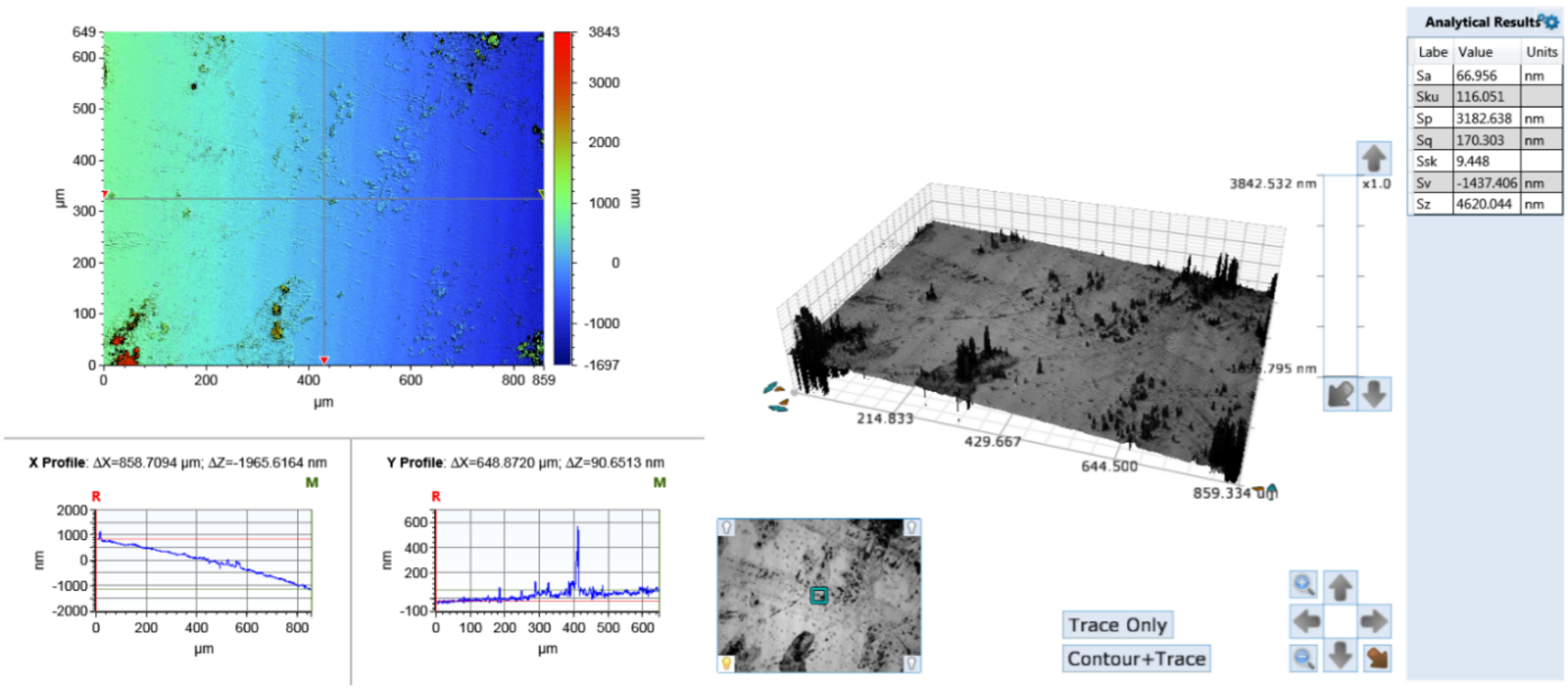

Polyethylene (PE), Sa = 243nm

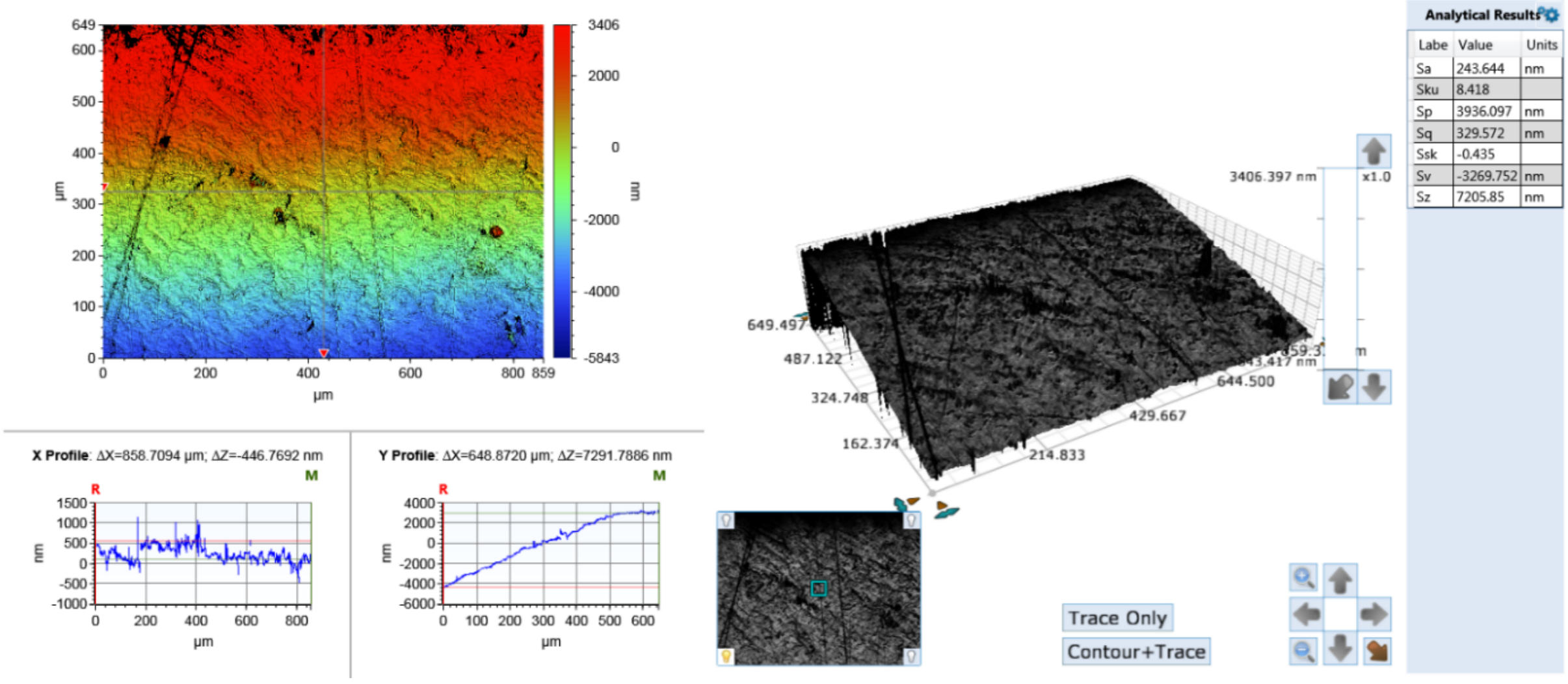

Polypropylene (PP), Sa = 89nm

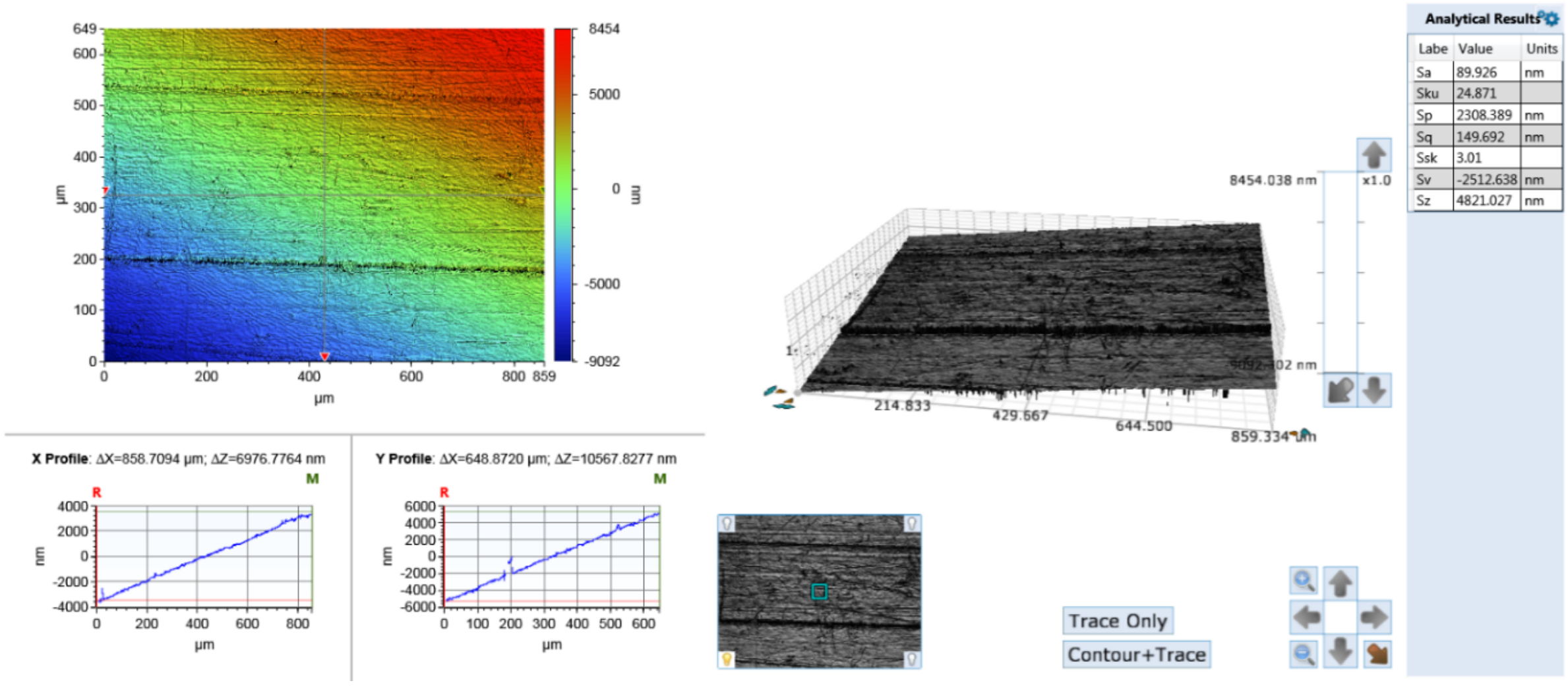

Polyethylene terephthalate (PET), Sa = 35nm

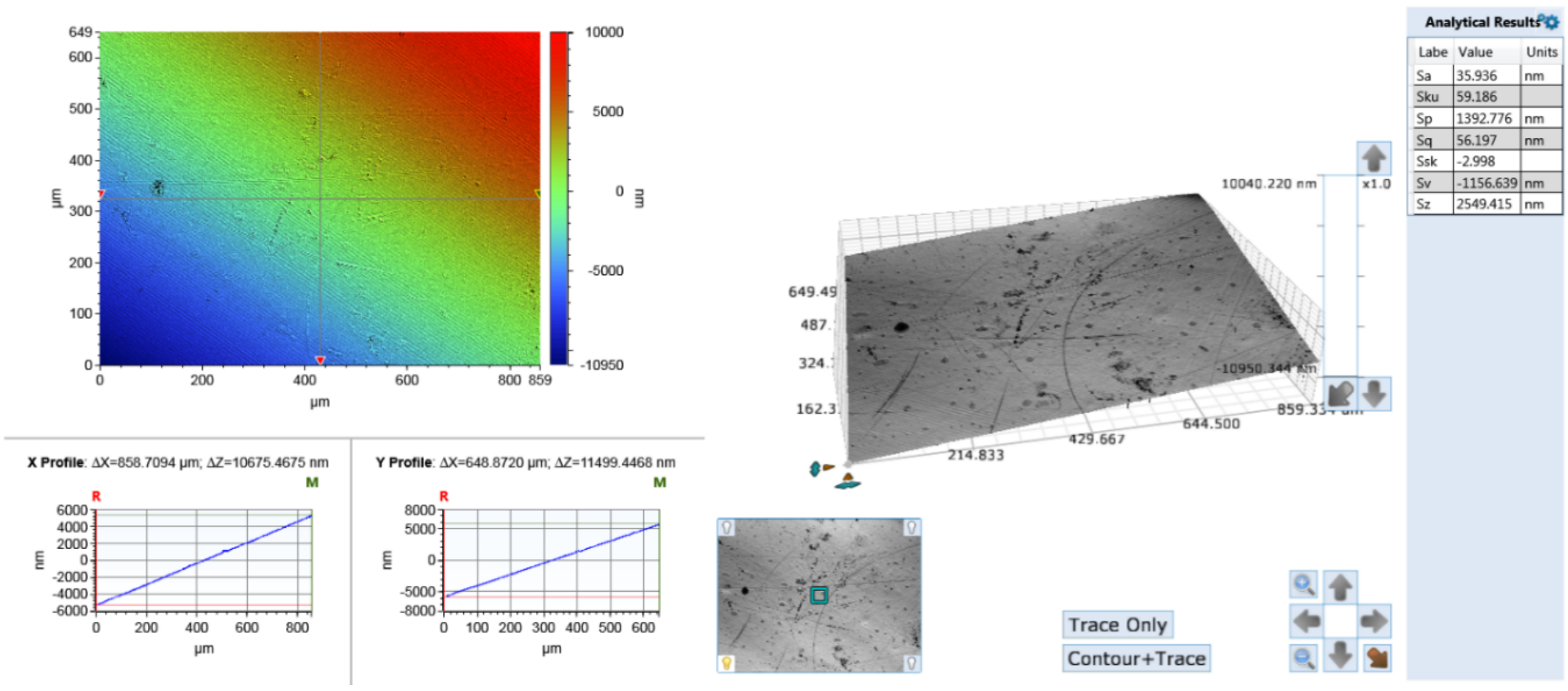

Polymethyl methacrylate (PMMA), Sa = 33nm

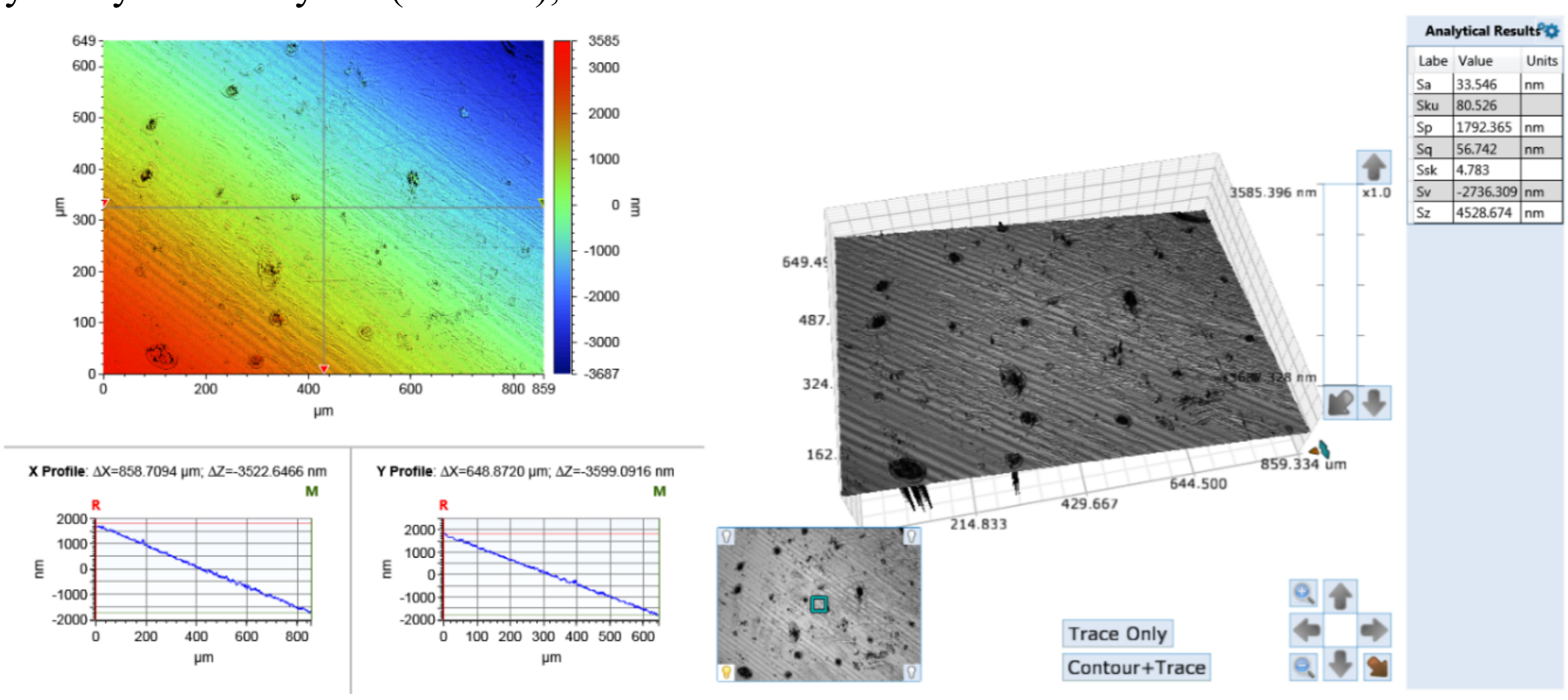

Acrylonitrile Butadiene Styrene (ABS), Sa = 96nm

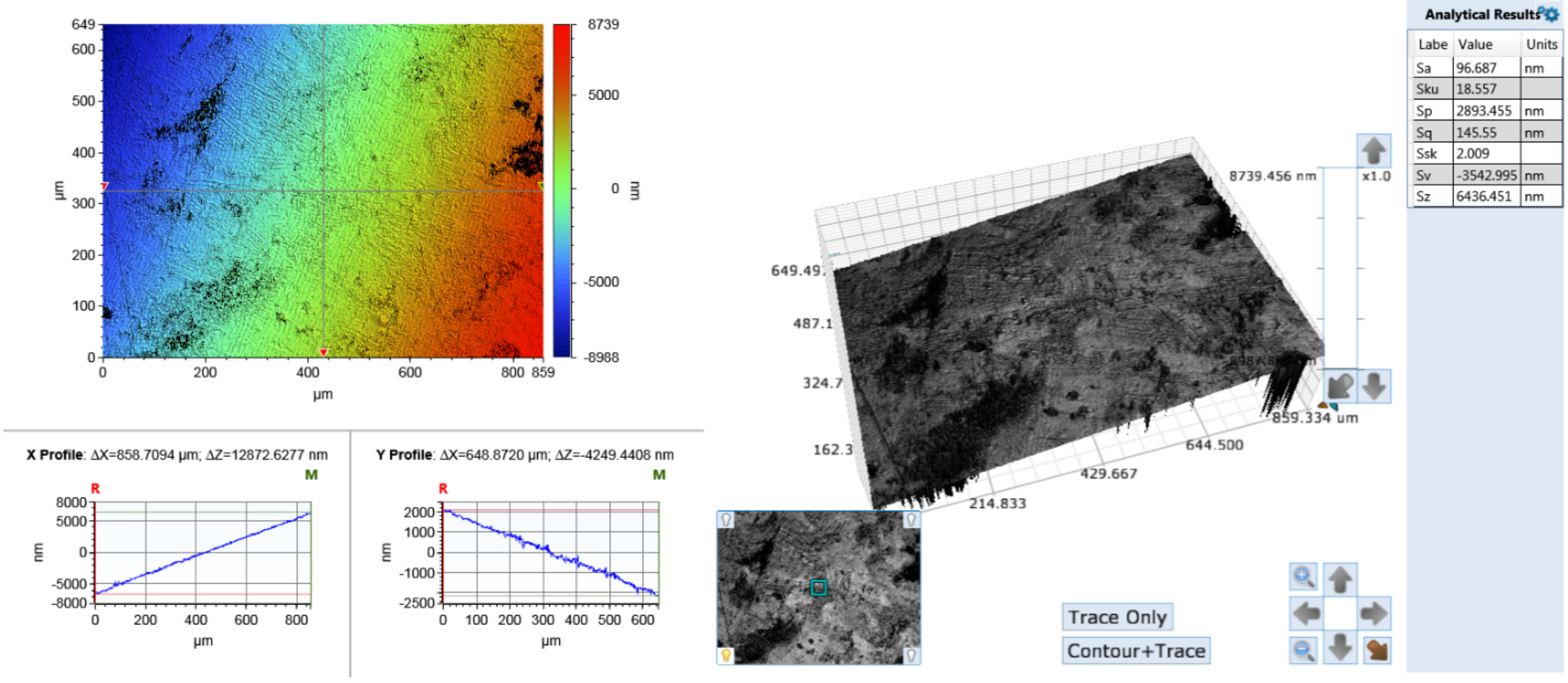

Fluorinated Ethylene Propylene (FEP), Sa = 140nm

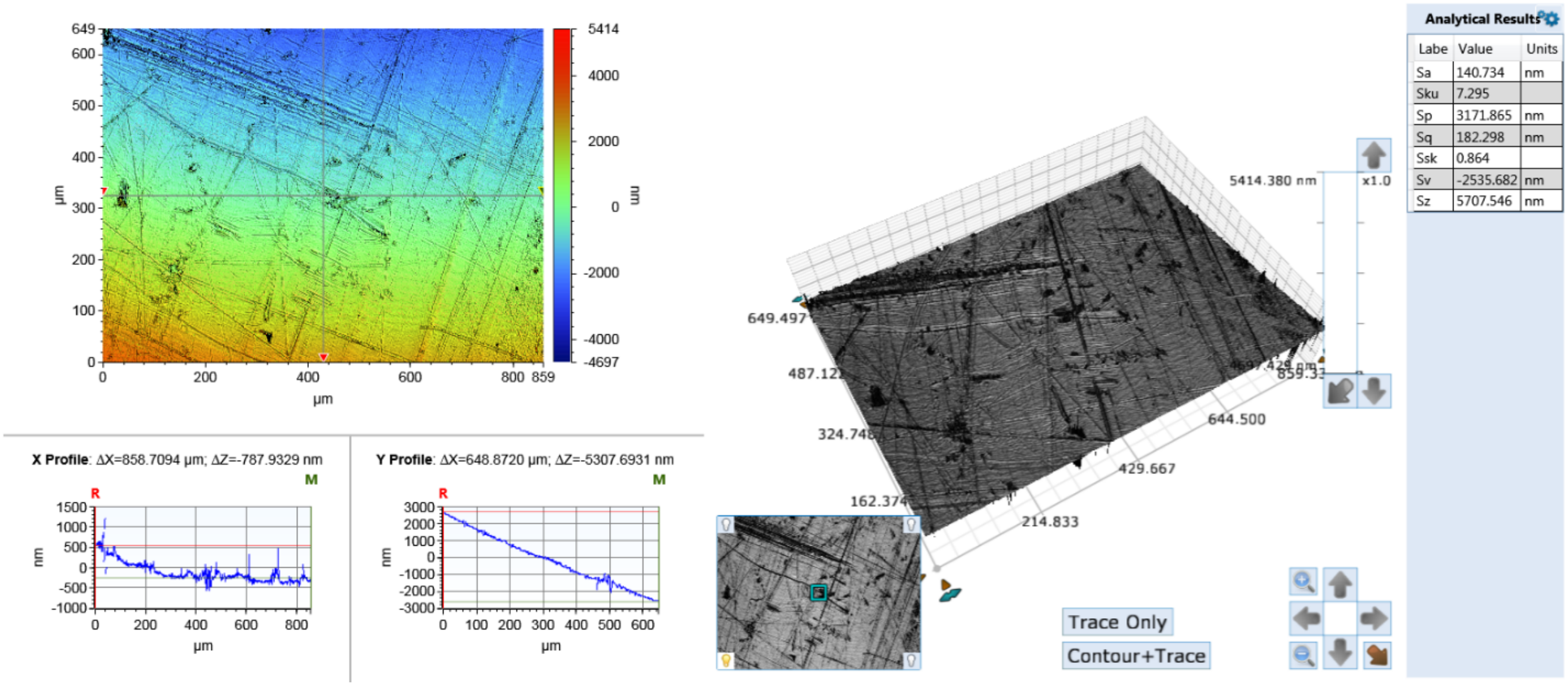

Polycaprolactum aramid 6 (Nylon6), Sa = 60nm

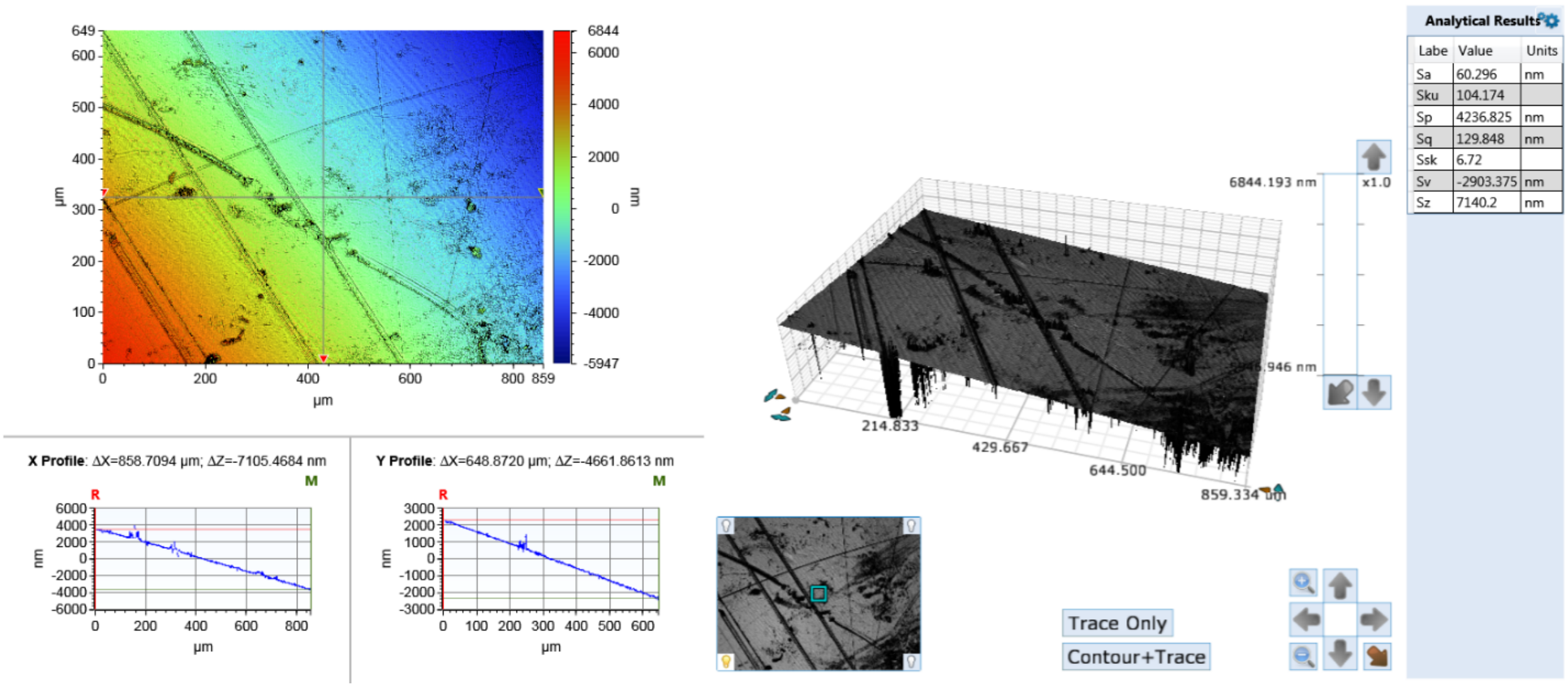

Polychlorotrifluoroethylene (PCTFE), Sa = 74nm

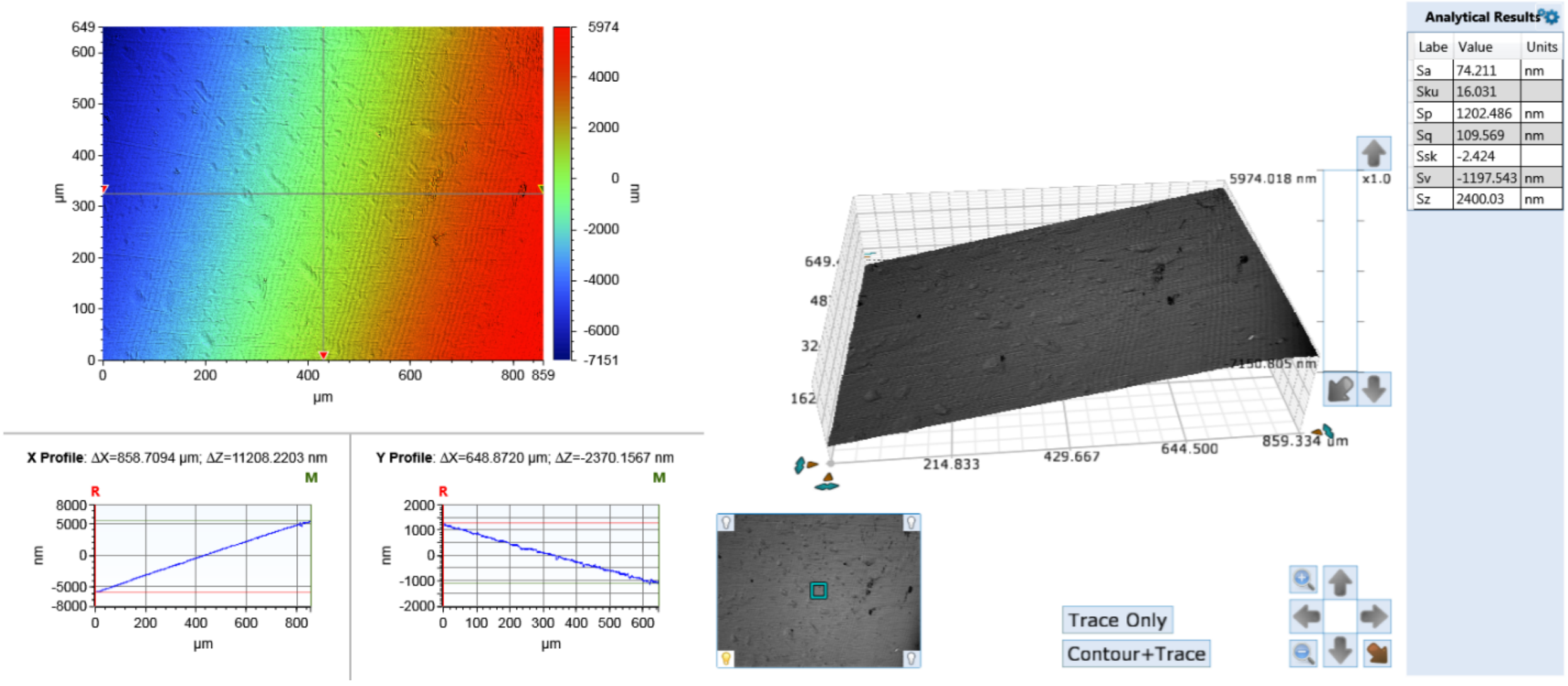

Polystyrene (PS), Sa = 33nm

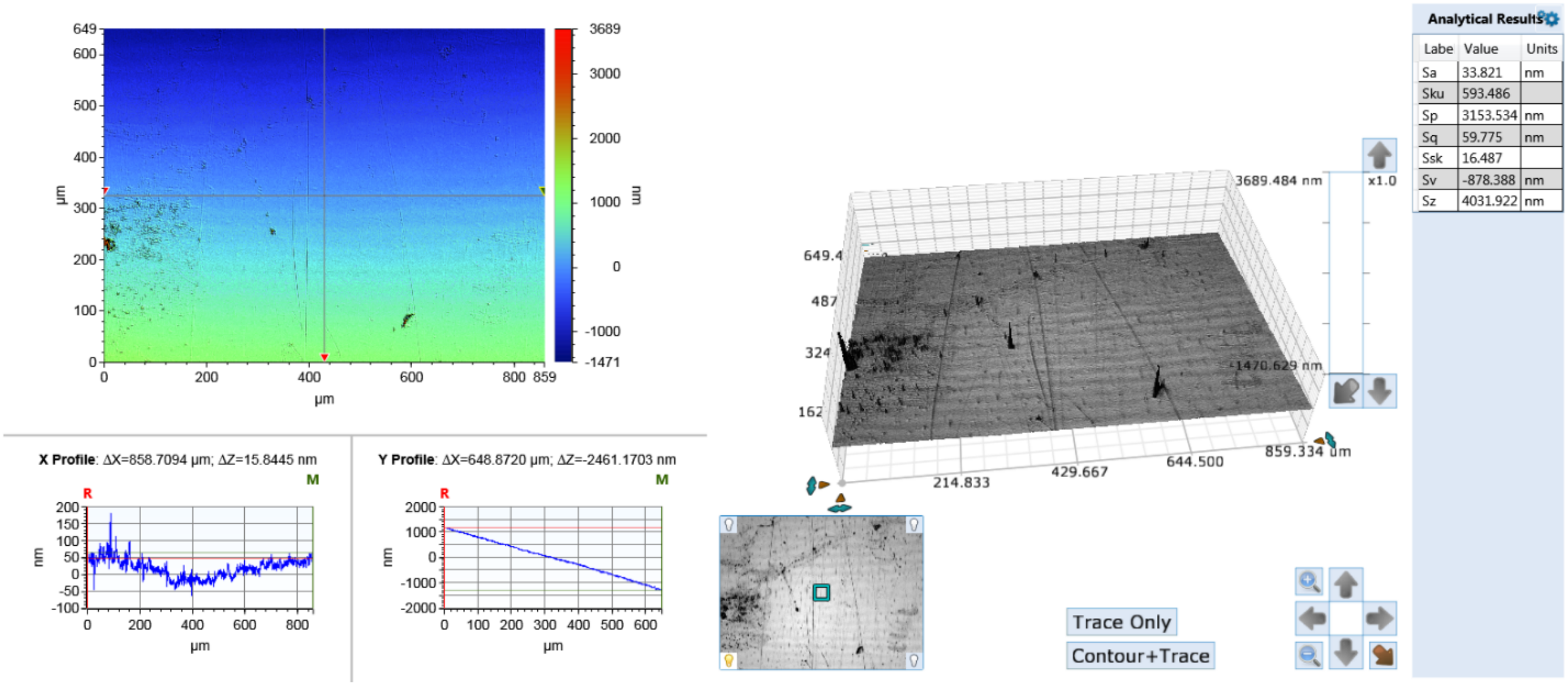

Polytetrafluoroethylene (PTFE), Sa = 462nm

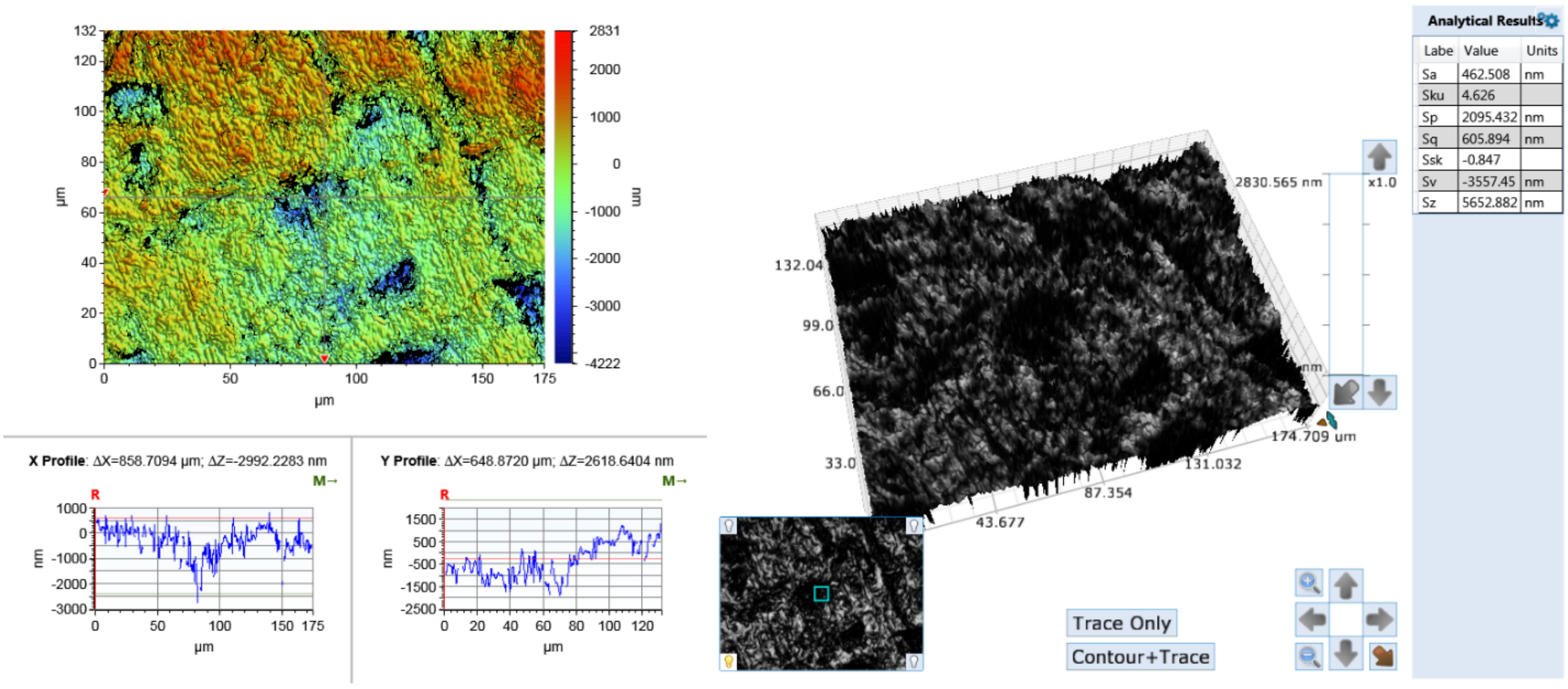

Polyvinylchloride (PVC), Sa = 43nm

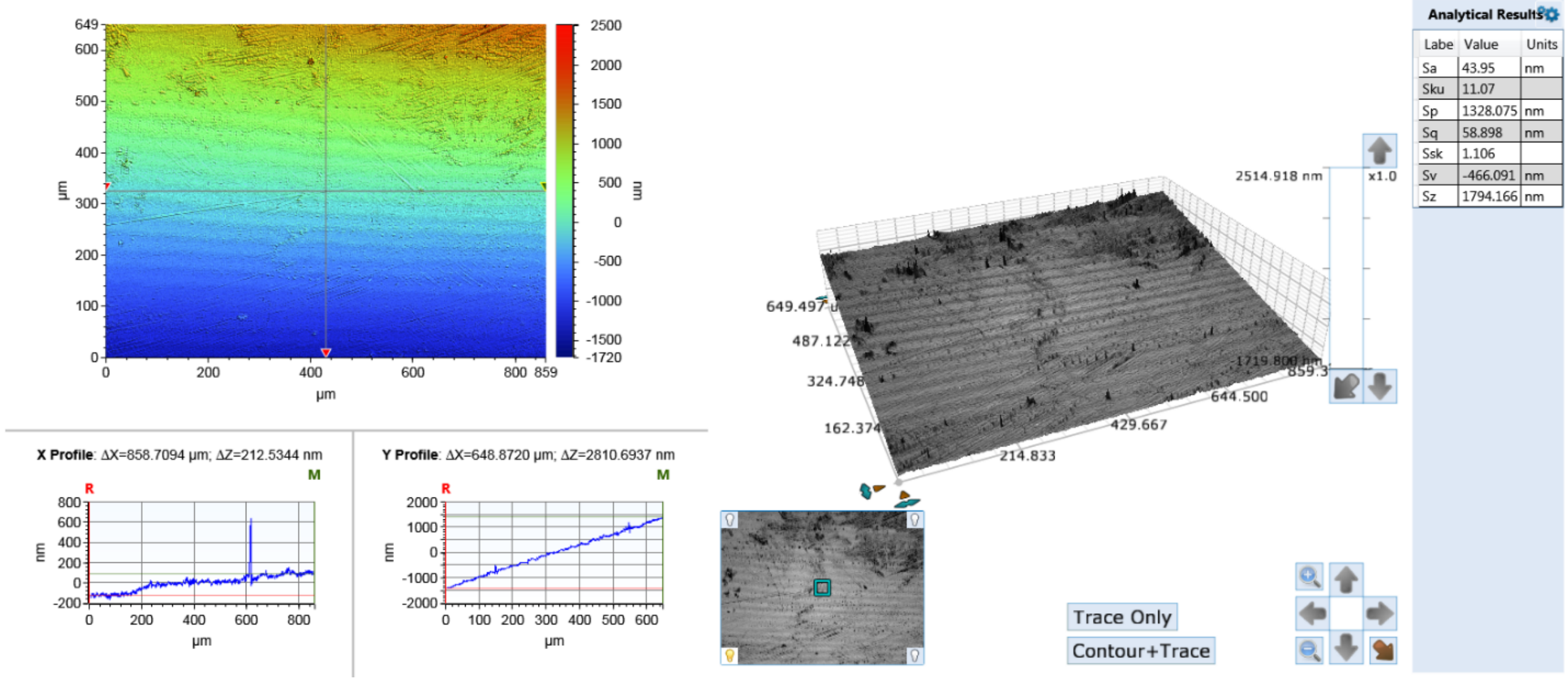

Polyvinylidene fluoride (PVDF), Sa = 62nm

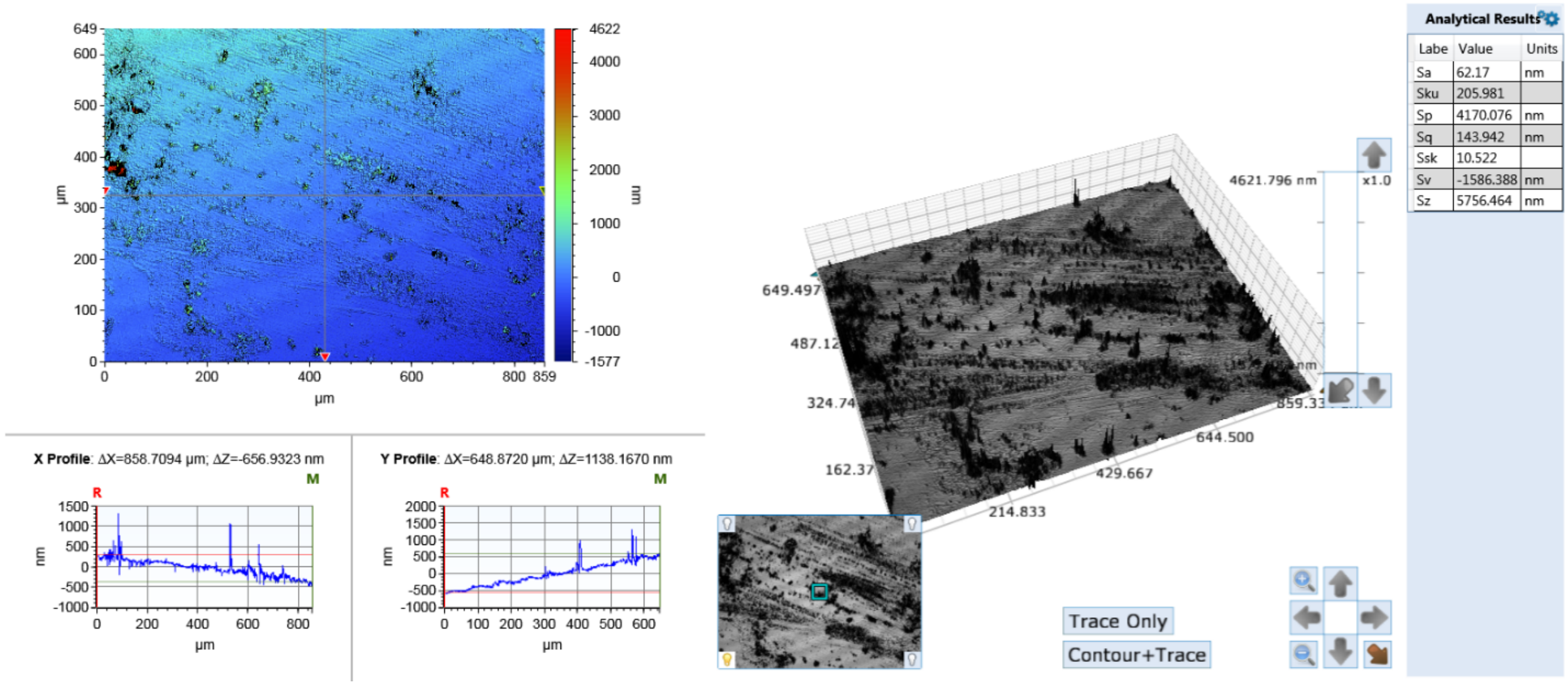

Polyether sulfone (PES), Sa = 71nm

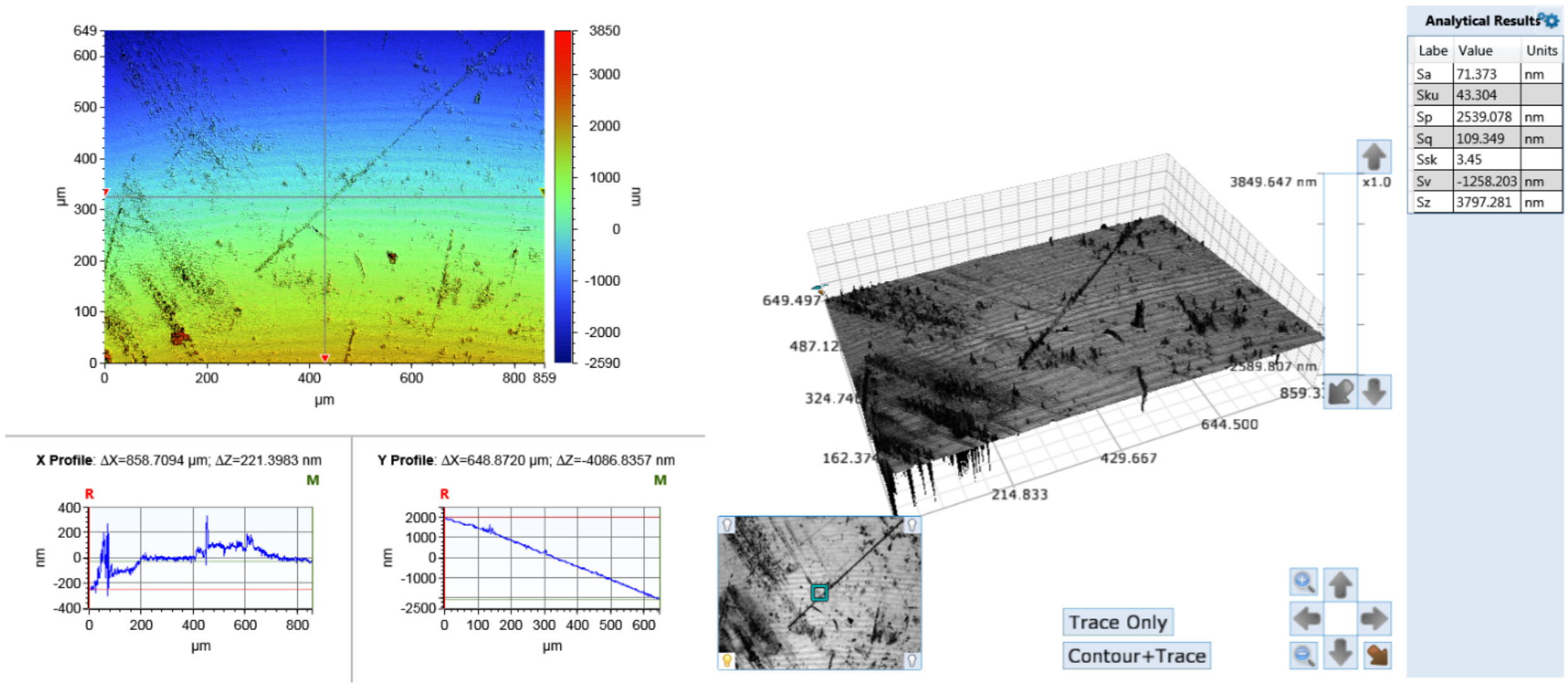

Polyphenylene oxide (PPO), Sa = 713nm

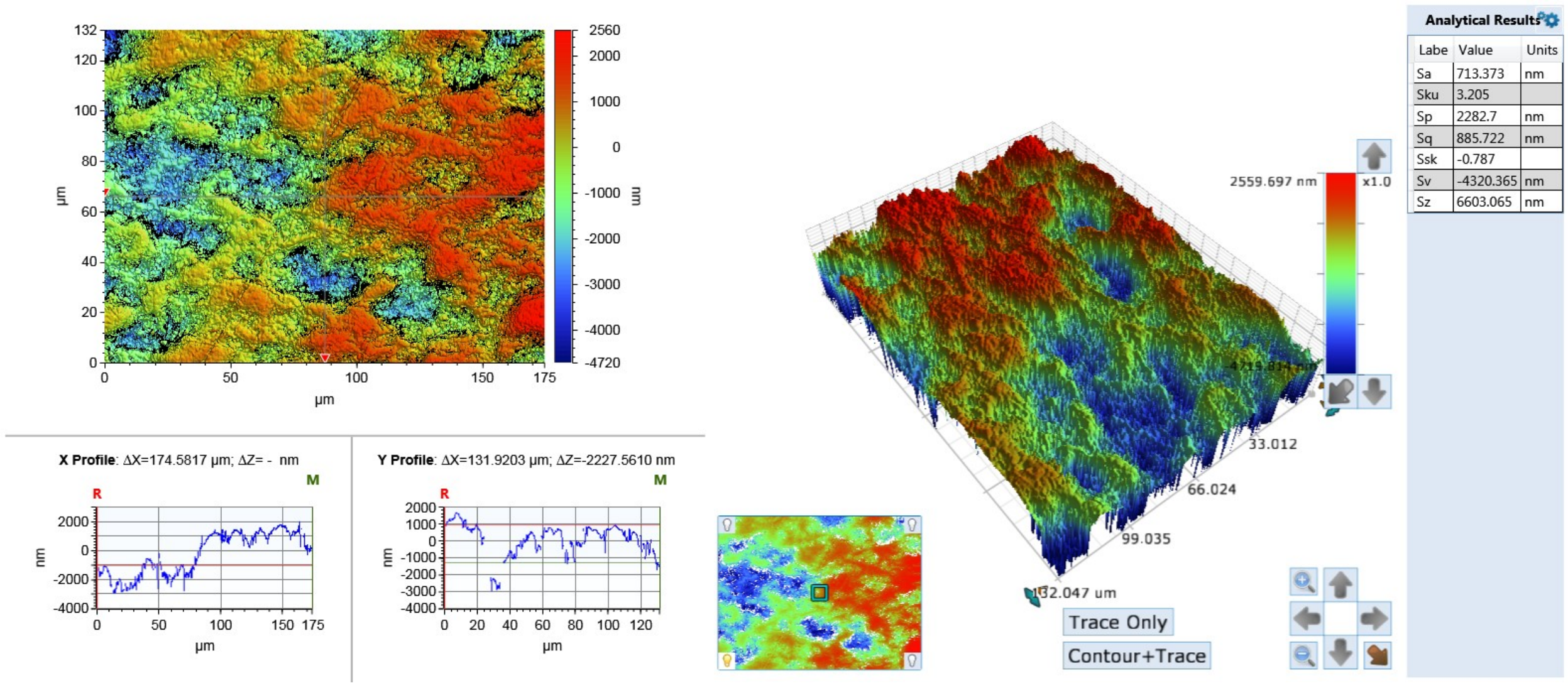

Polybutylene terephthalate (PBT), Sa = 818nm

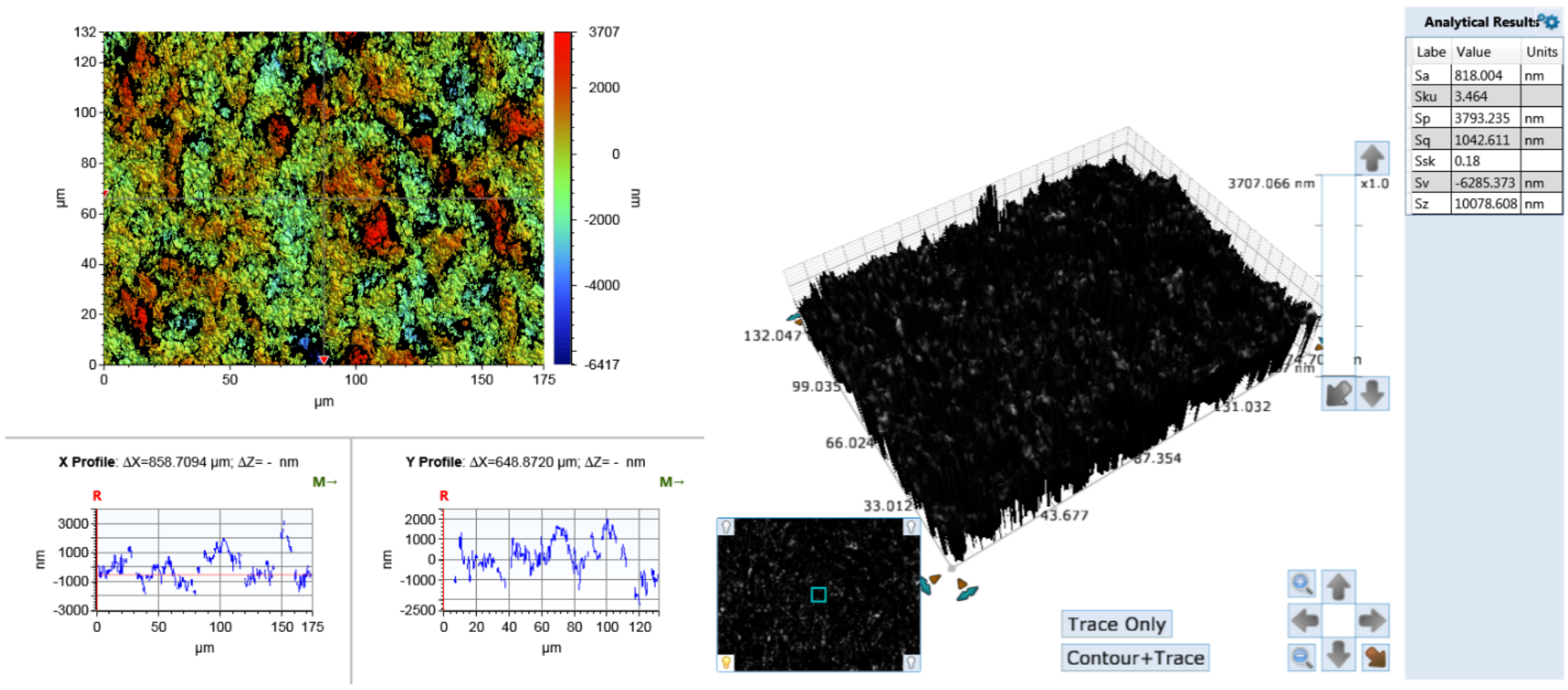

Differential scanning calorimetry measurements:

Polyether ether ketone (PEEK):

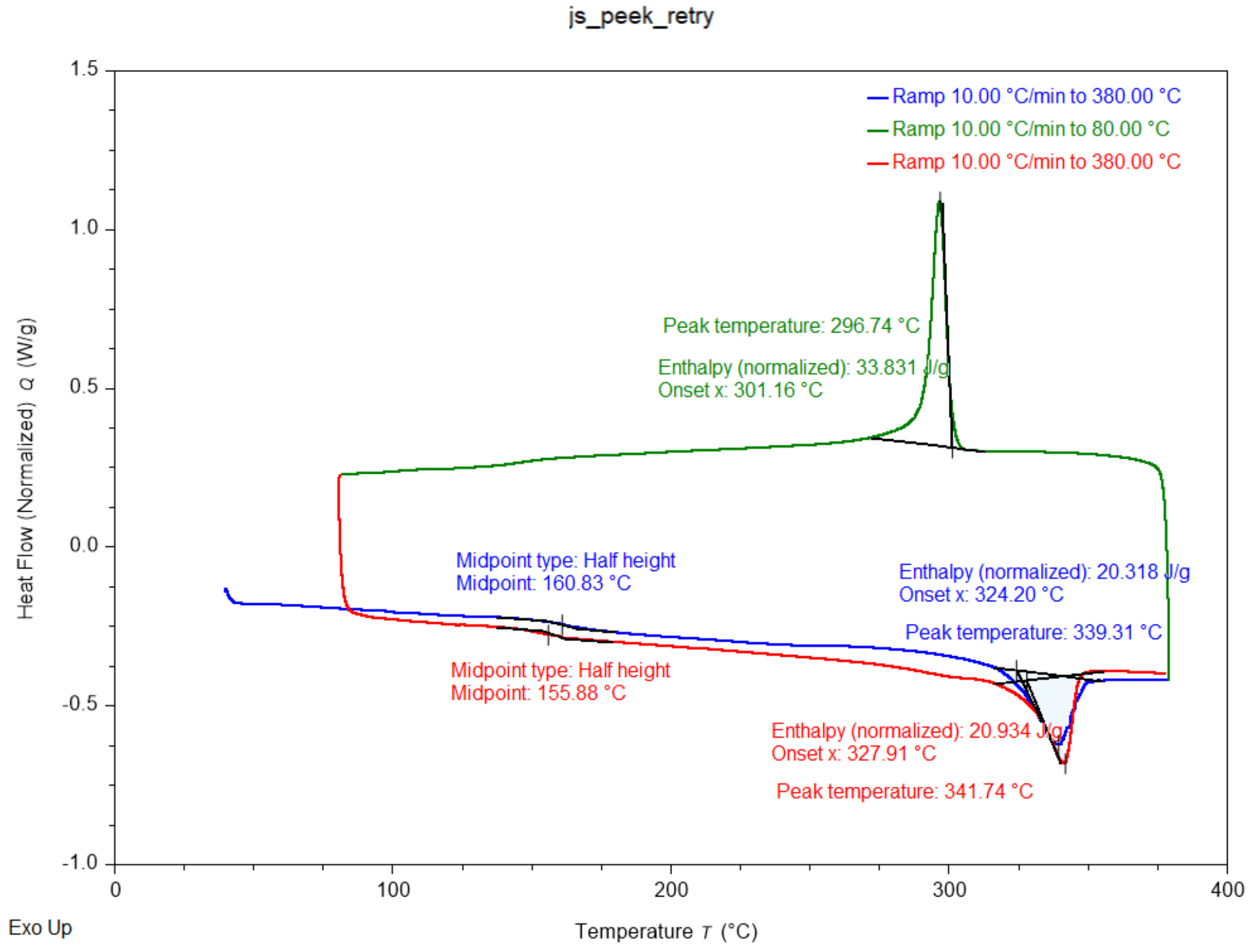

Polyphenylene sulfide (PPS):

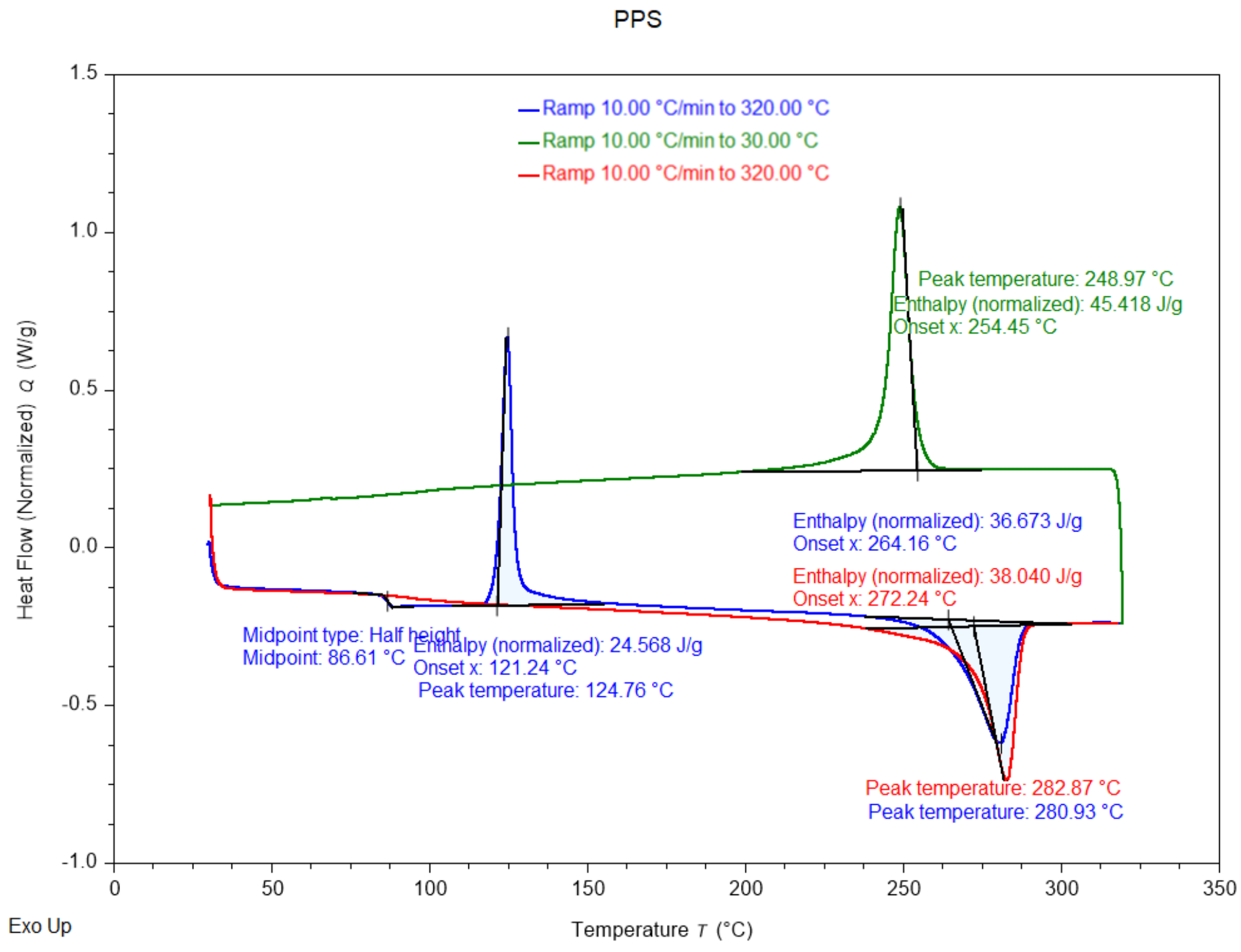

Polysulfone (PSU):

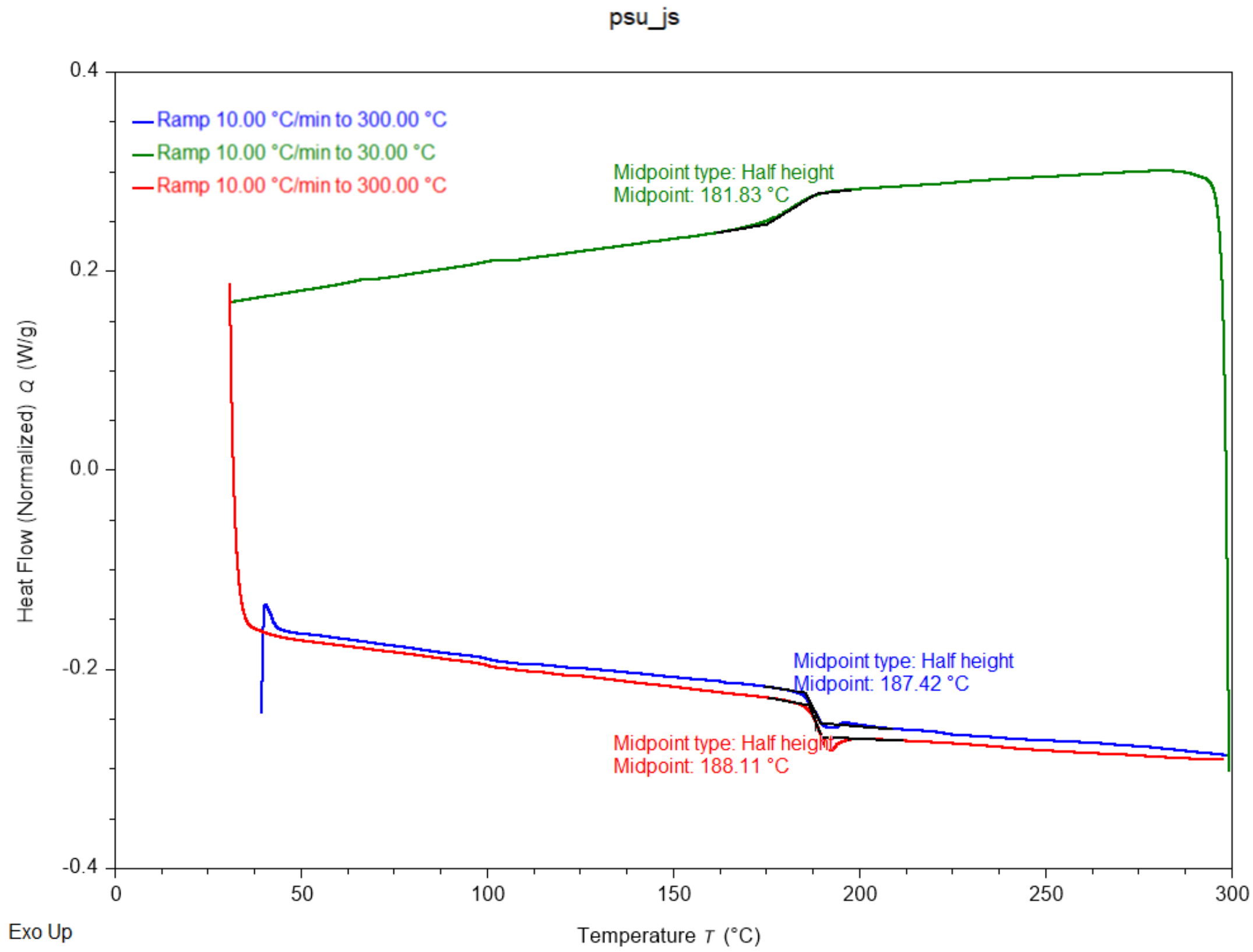

Polycarbonate (PC):

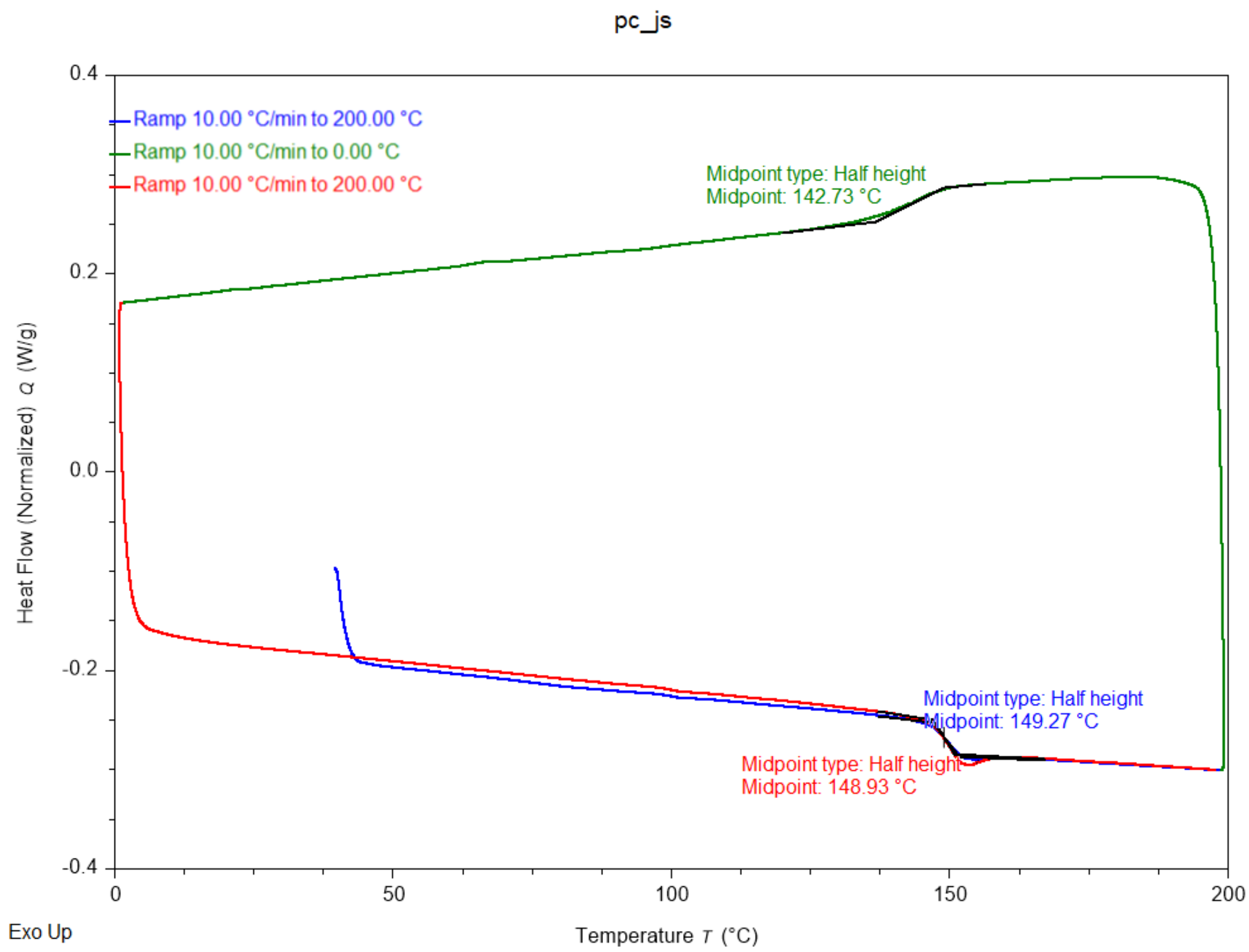

Polyethylene (PE):

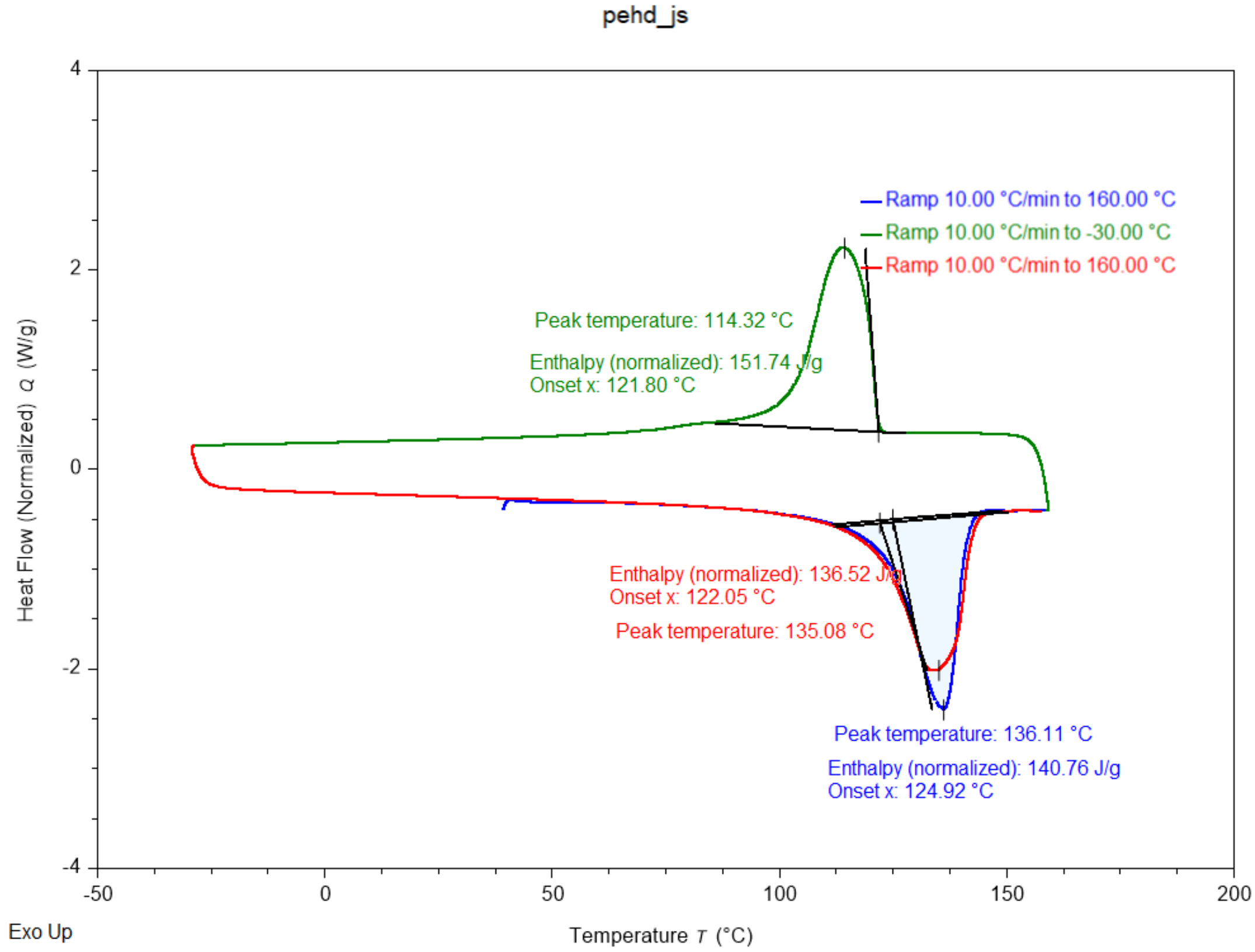

Polypropylene (PP):

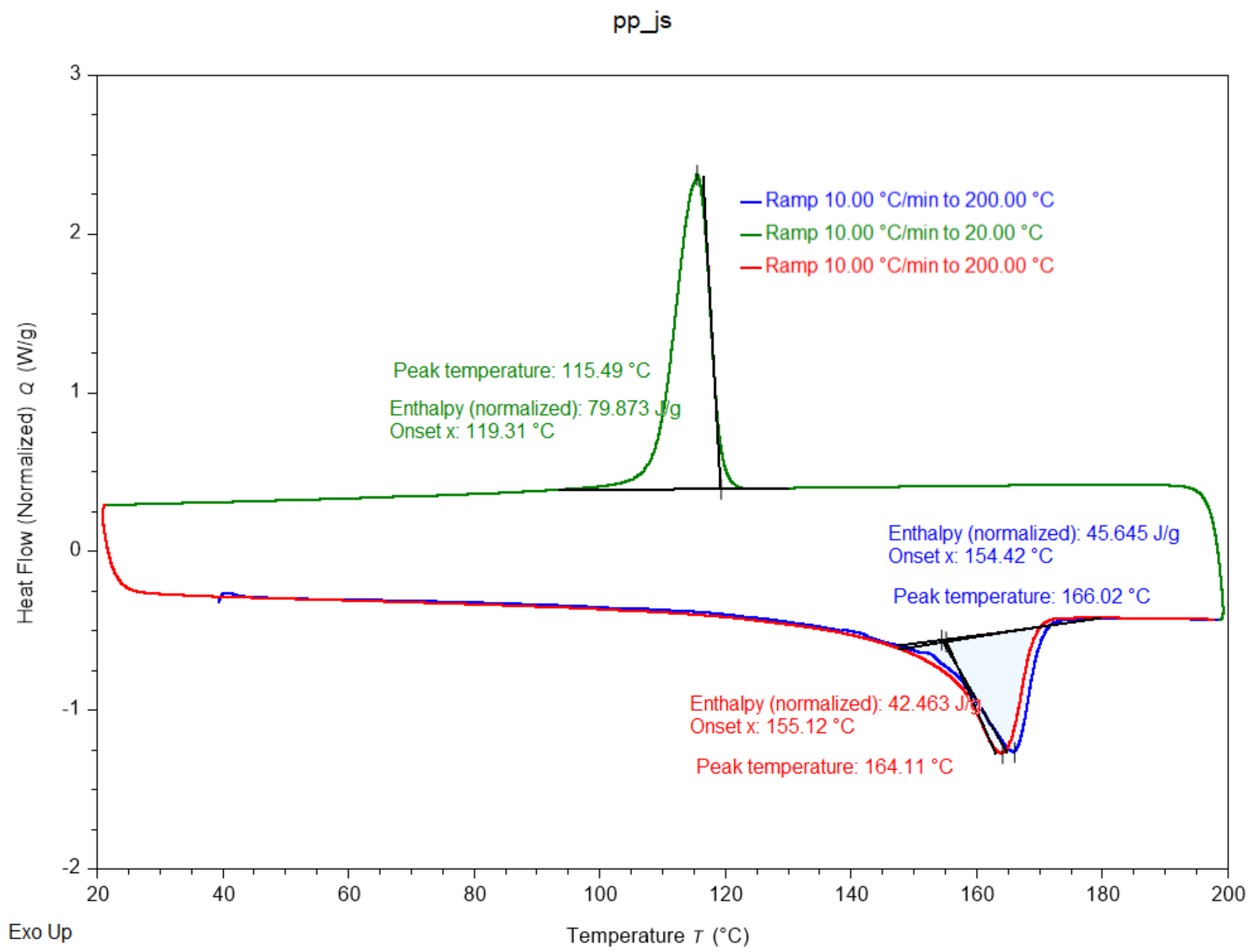

Polyethylene terephthalate (PET):

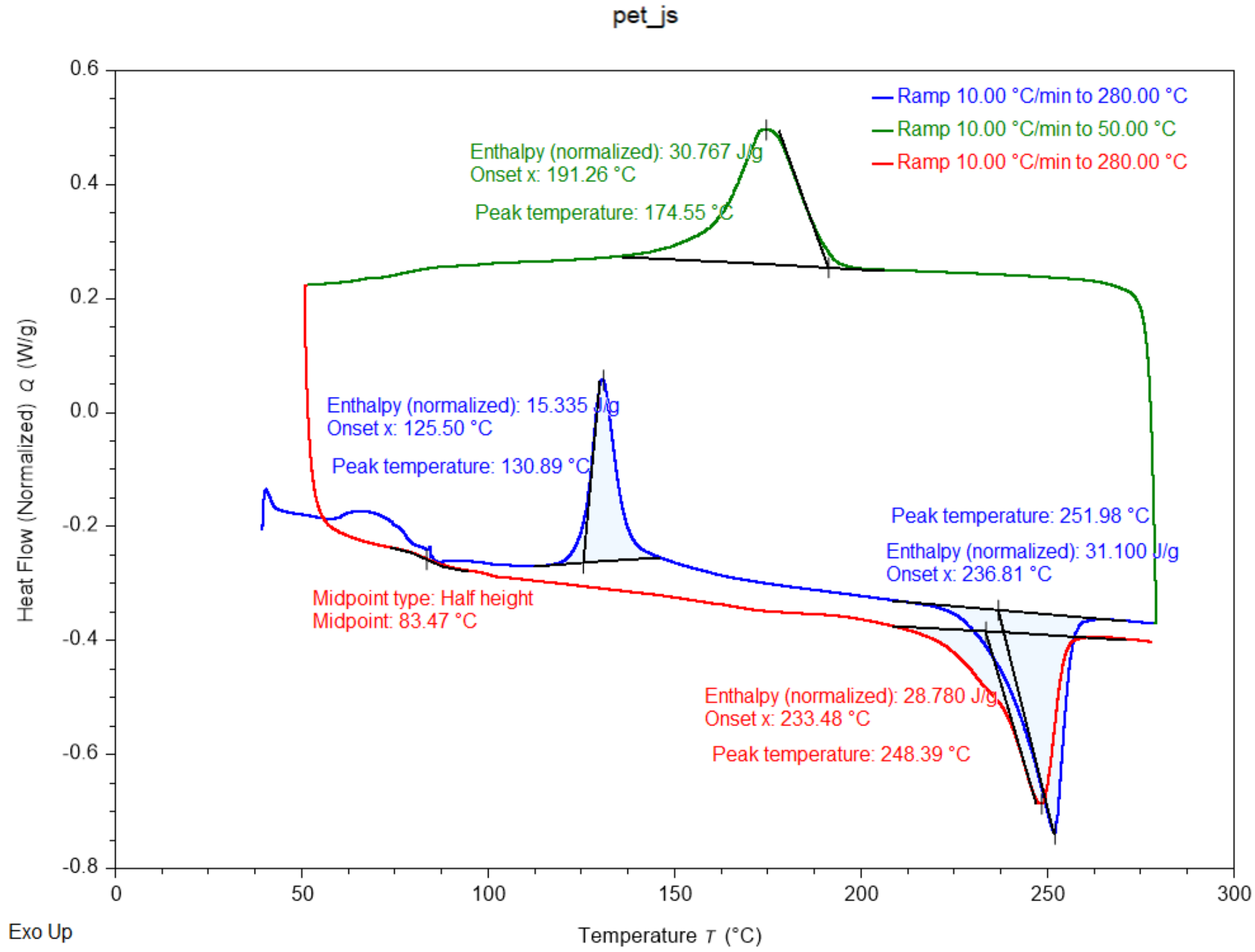

Polymethyl methacrylate (PMMA):

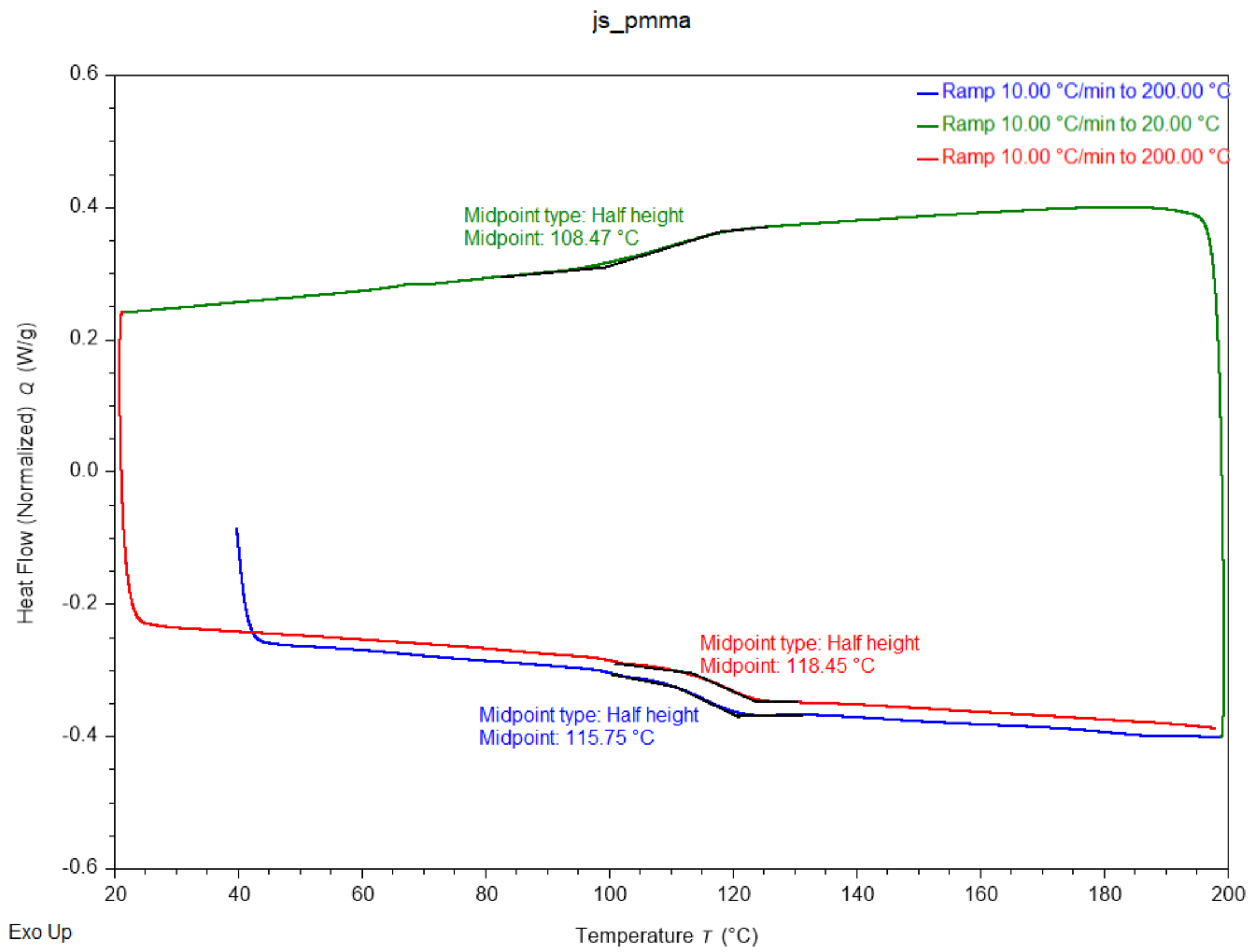

Acrylonitrile butadiene styrene (ABS):

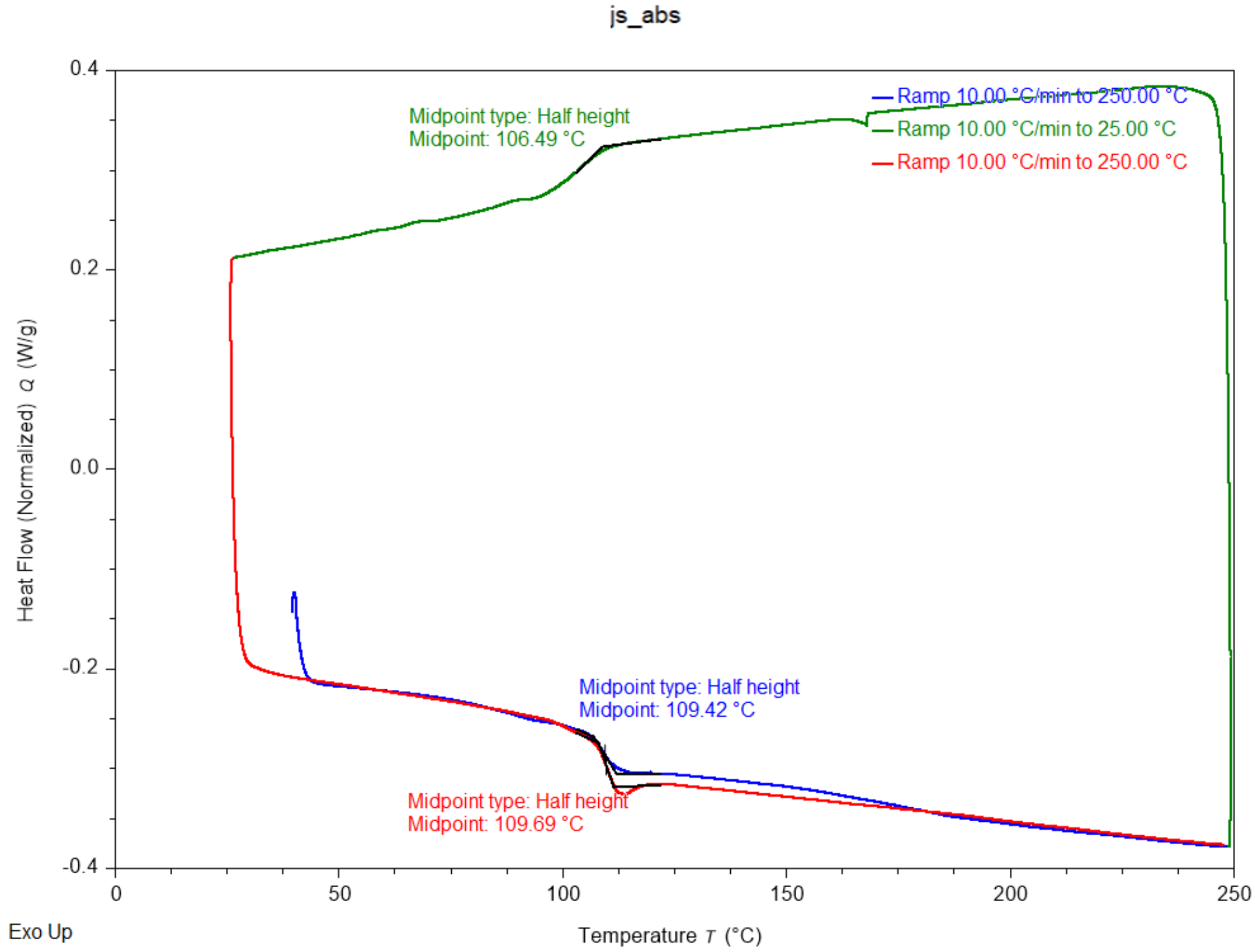

Fluorinated ethylene propylene (FEP):

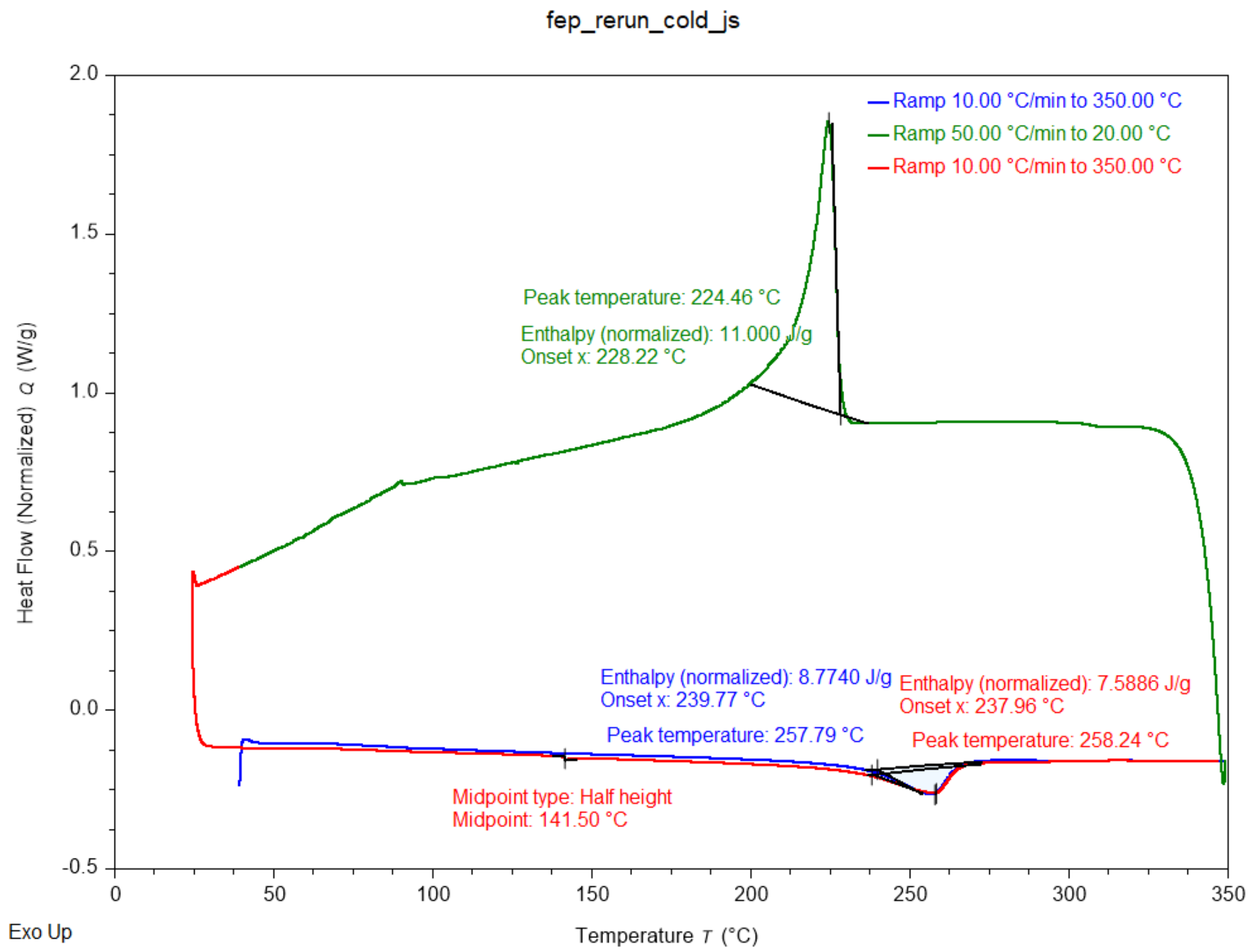

Polycrapolactum aramid 6 (NYLON6):

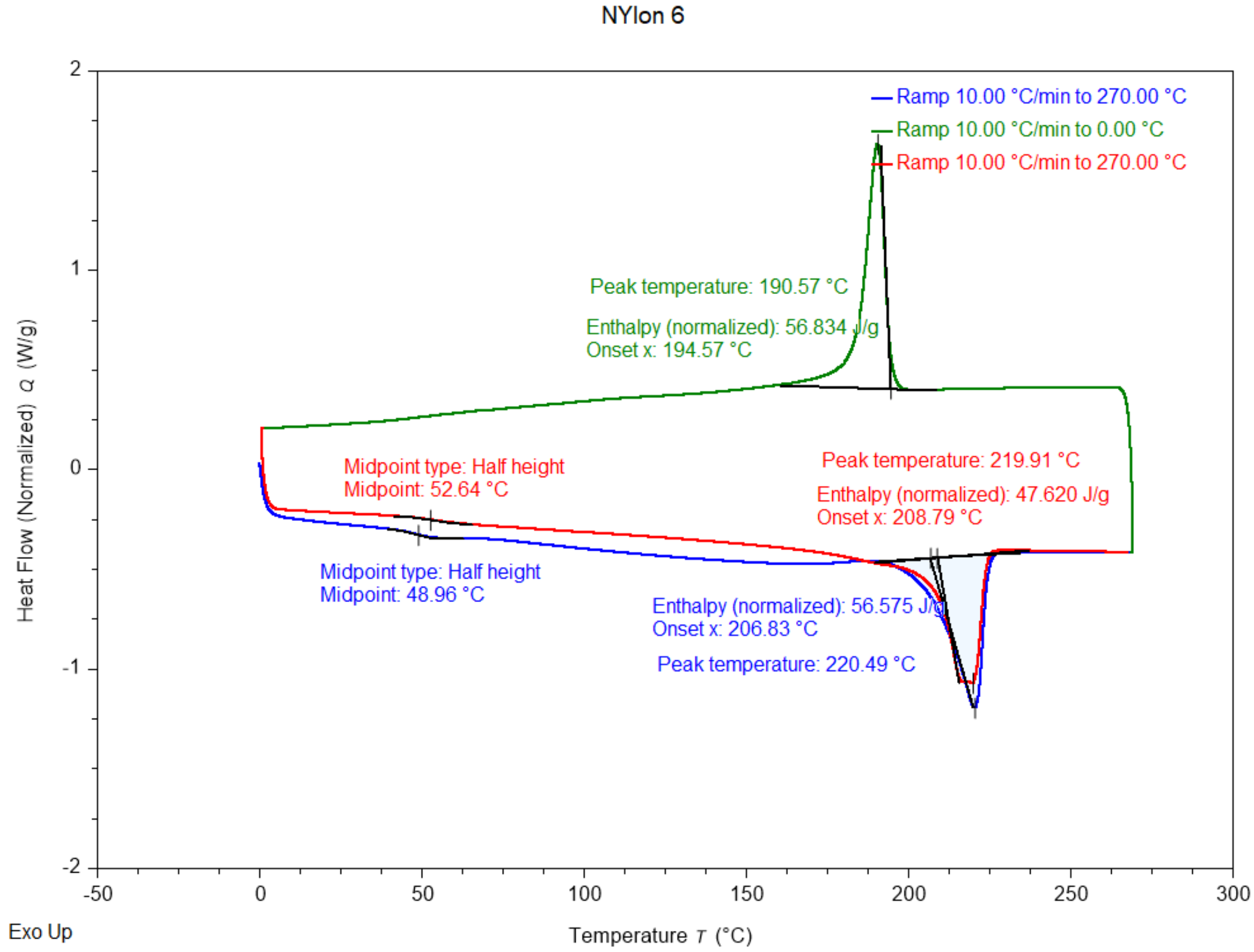

Polychlorotrifluoroethylene (PCTFE):

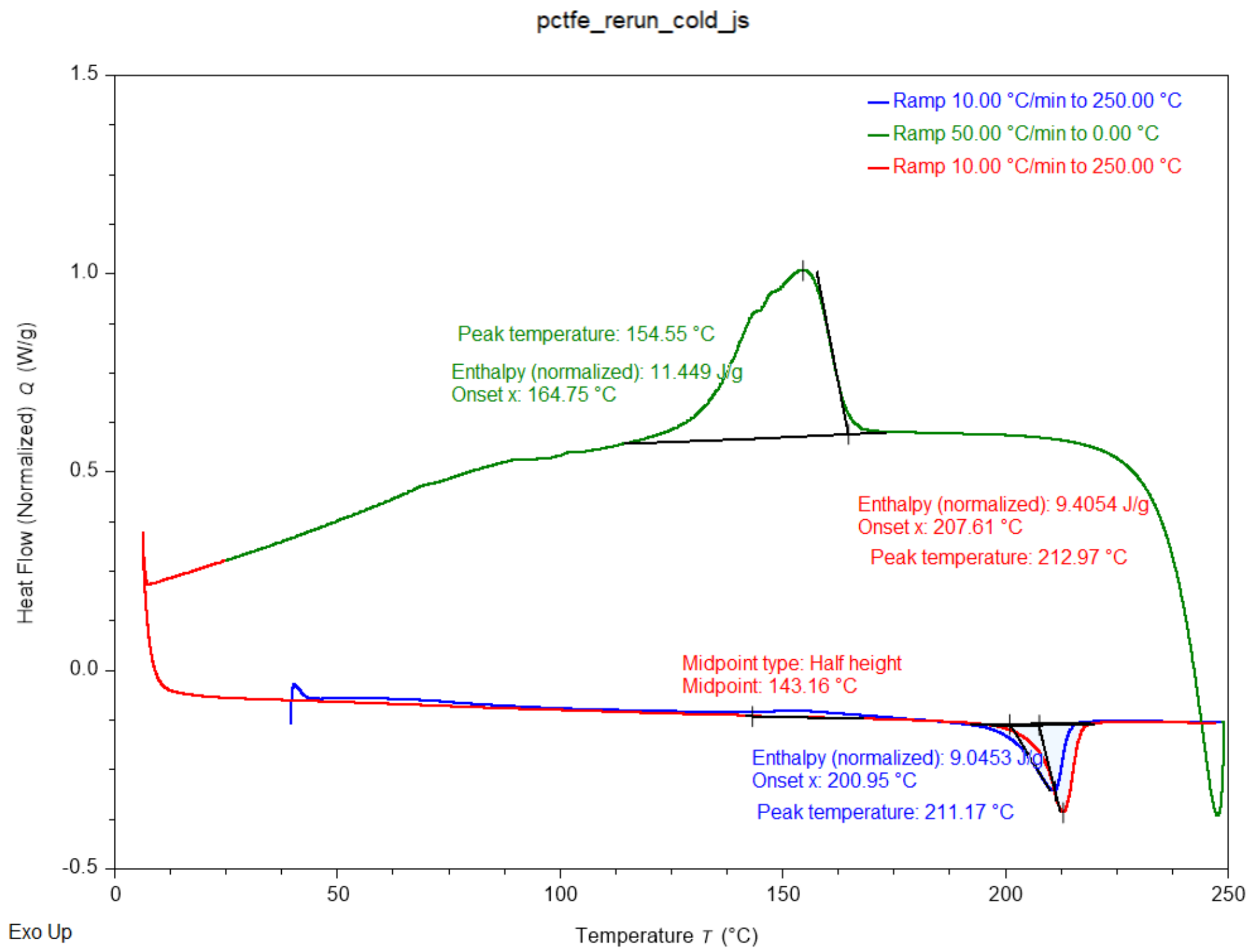

Polystyrene (PS):

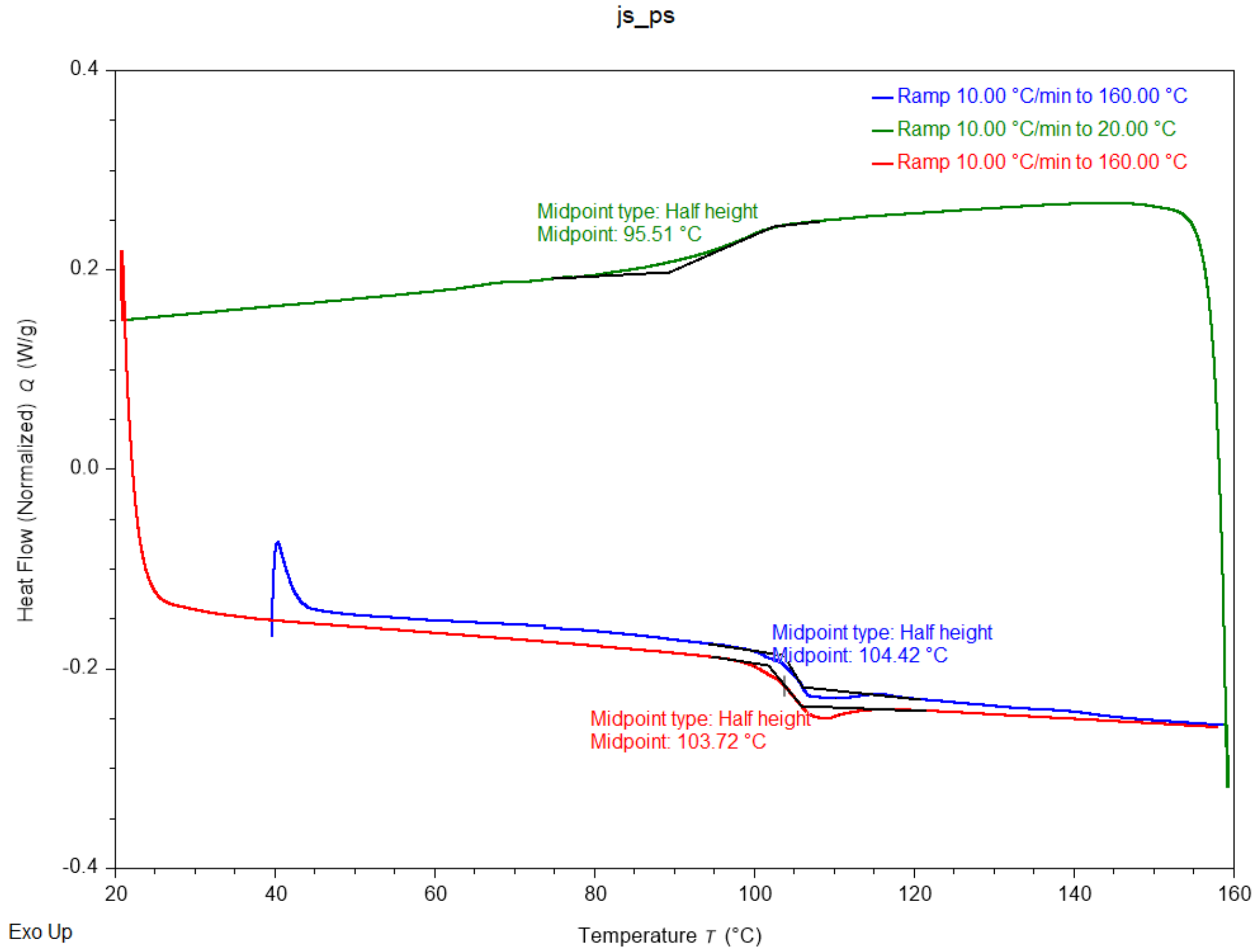

Polytetrafluoroethylene (PTFE):

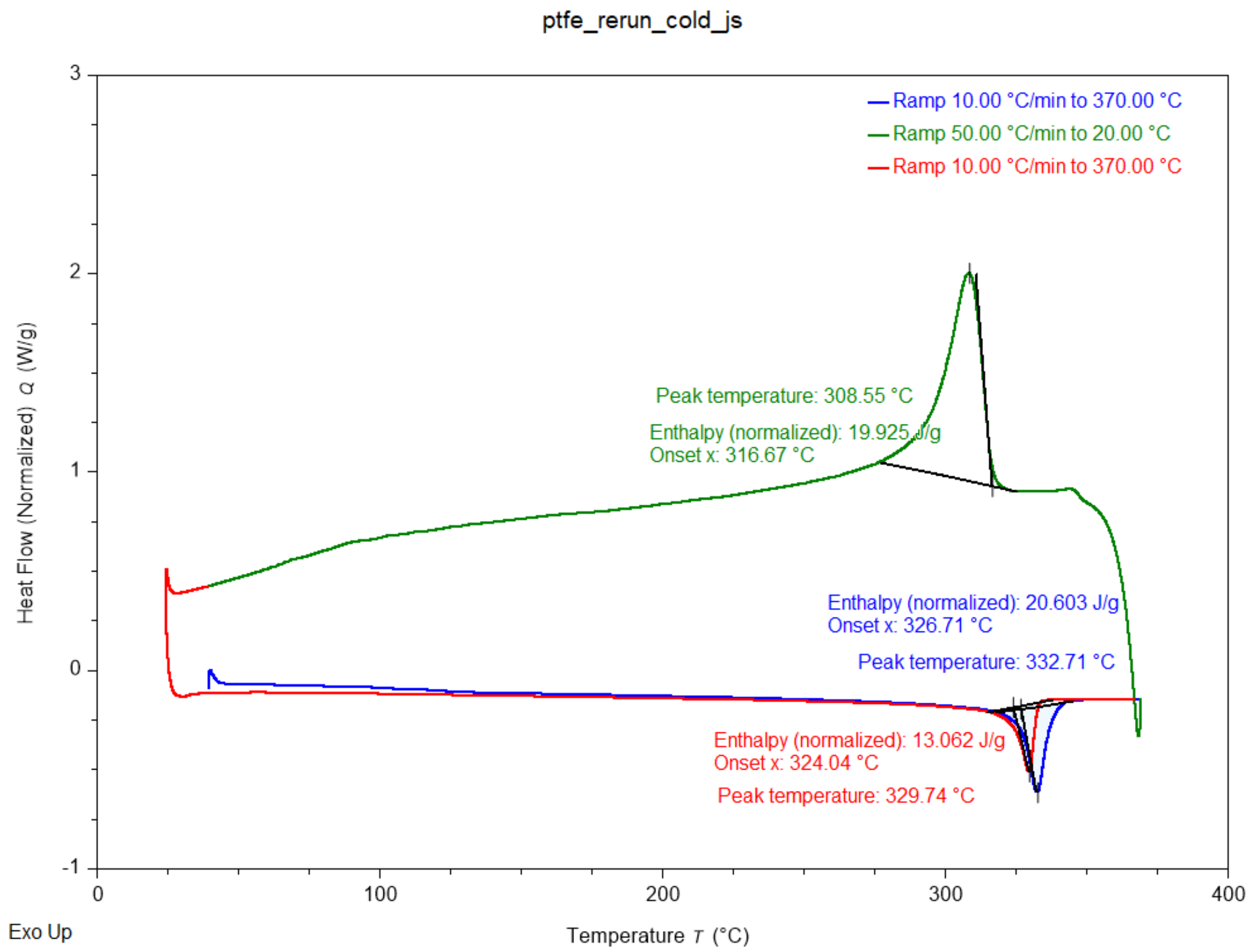

Unplasticized Polyvinylchloride (uPVC):

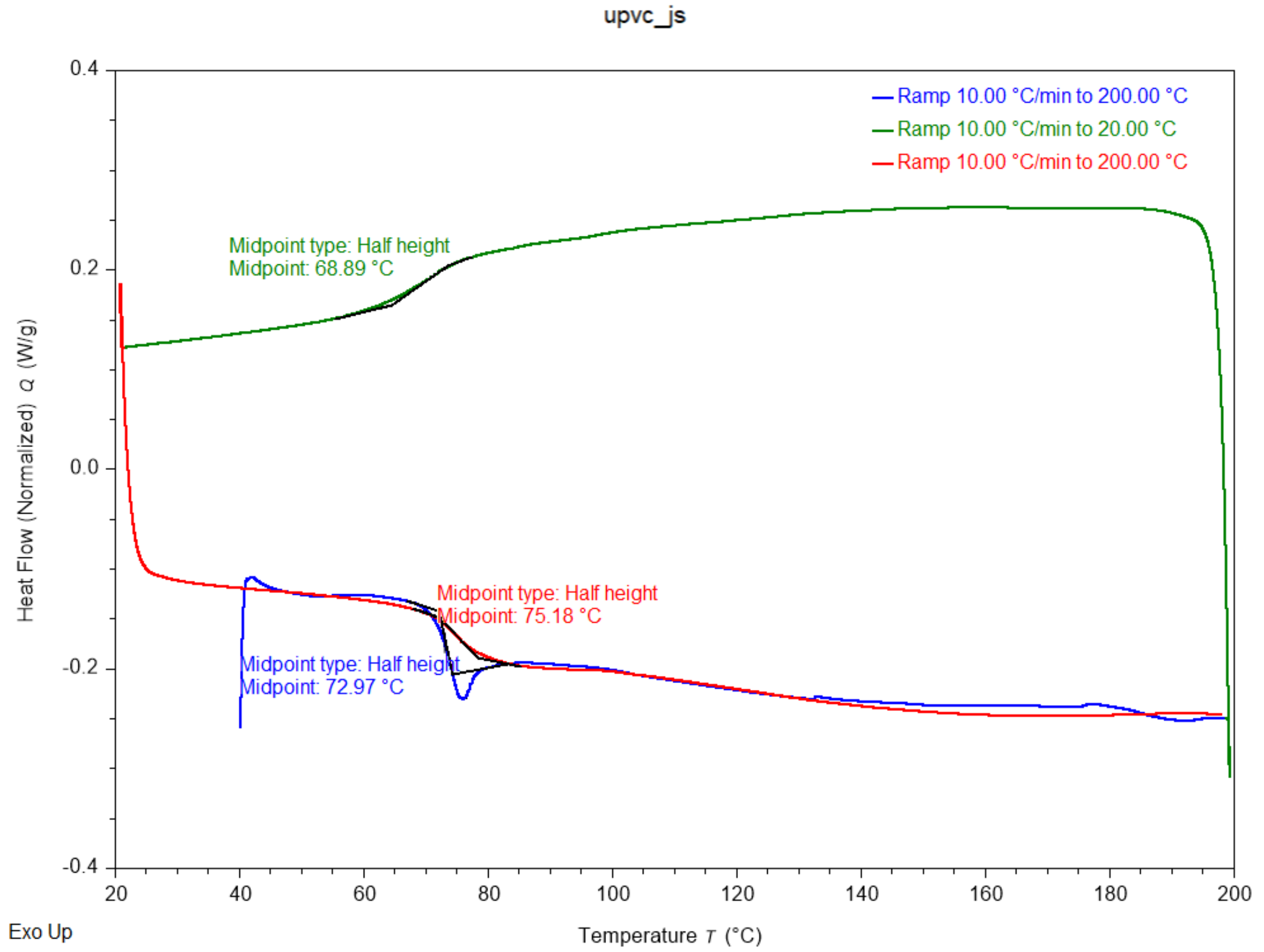

Polyvinylidene fluoride (PVDF):

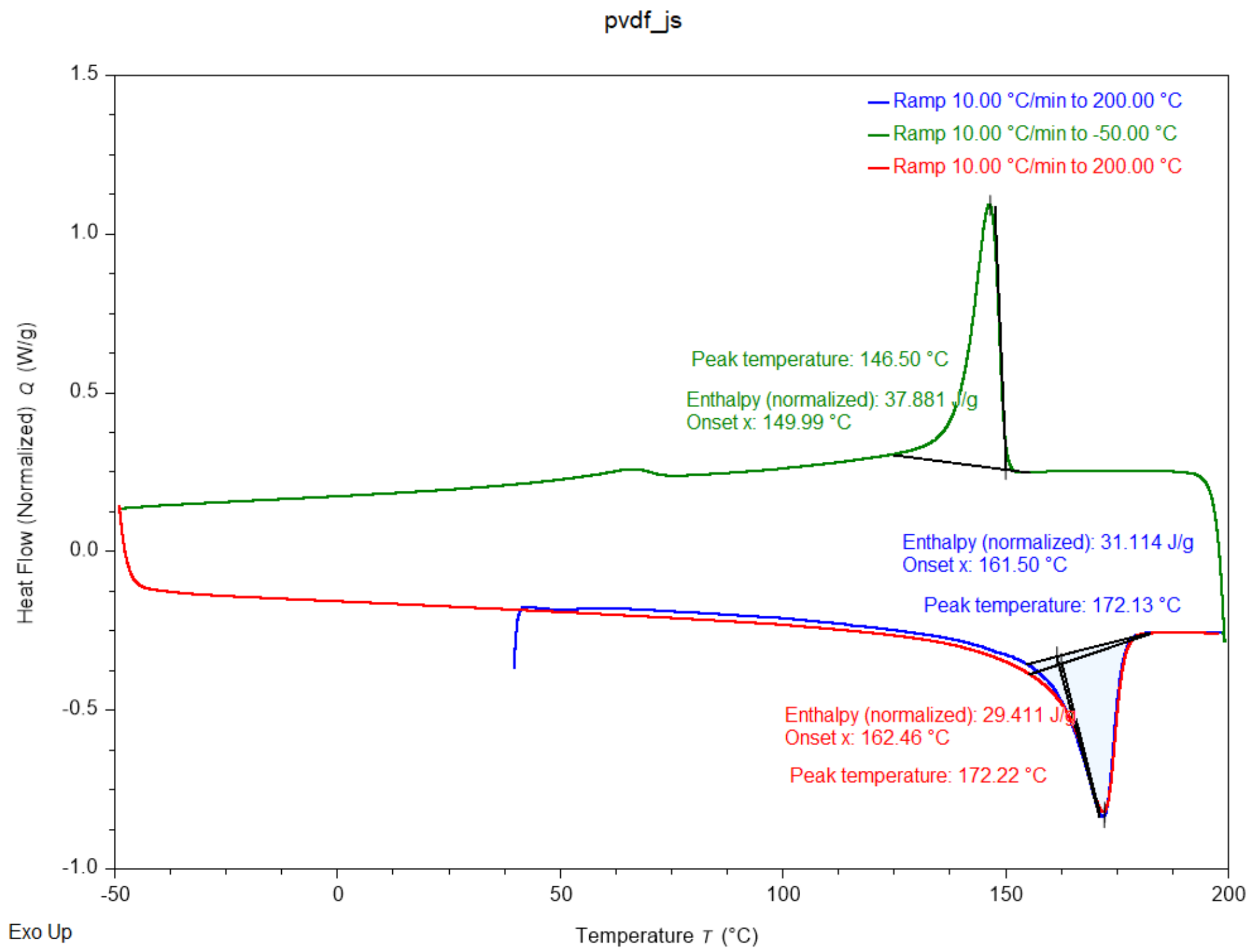

Polyether sulfone (PES):

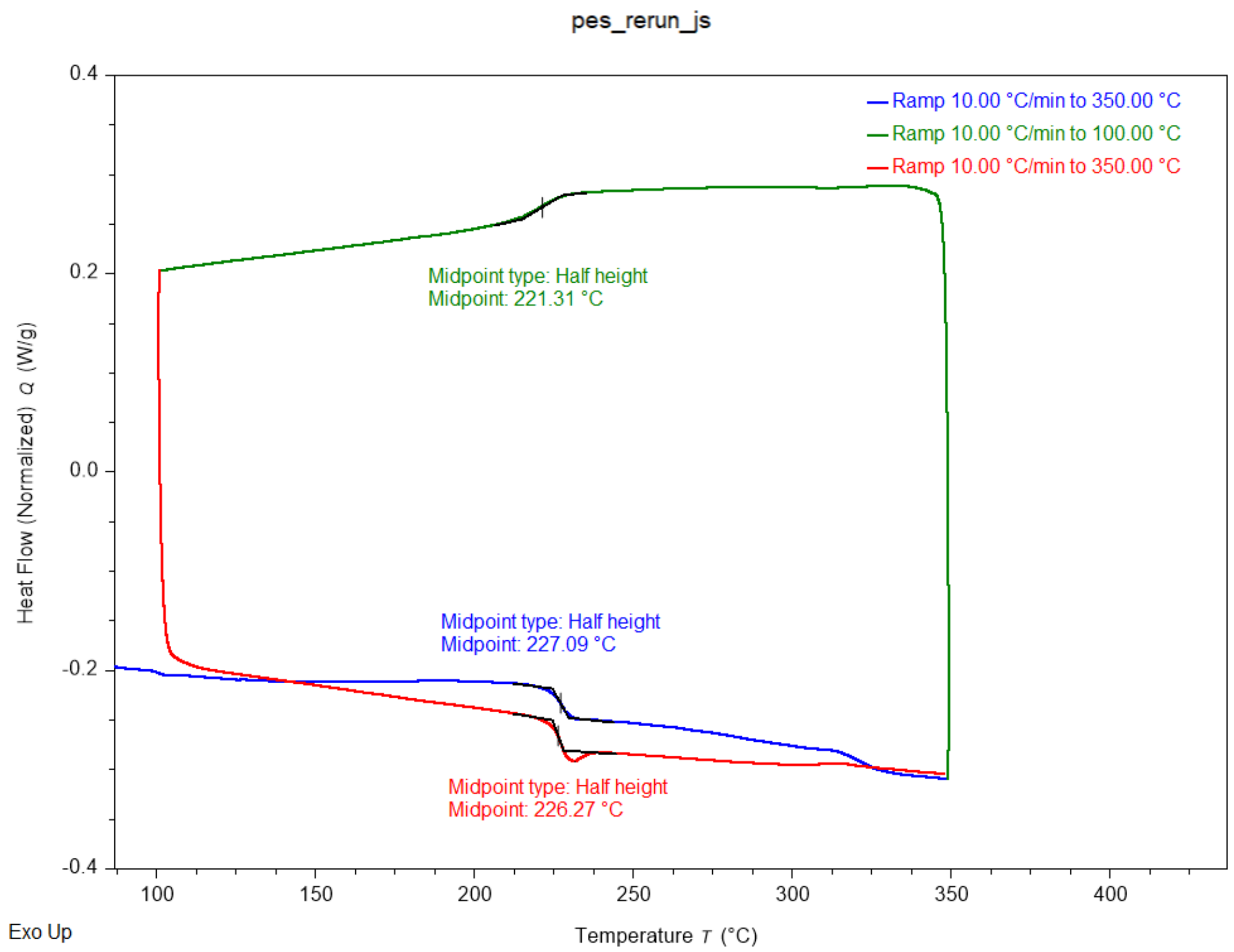

Polyphenylene oxide (PPO):

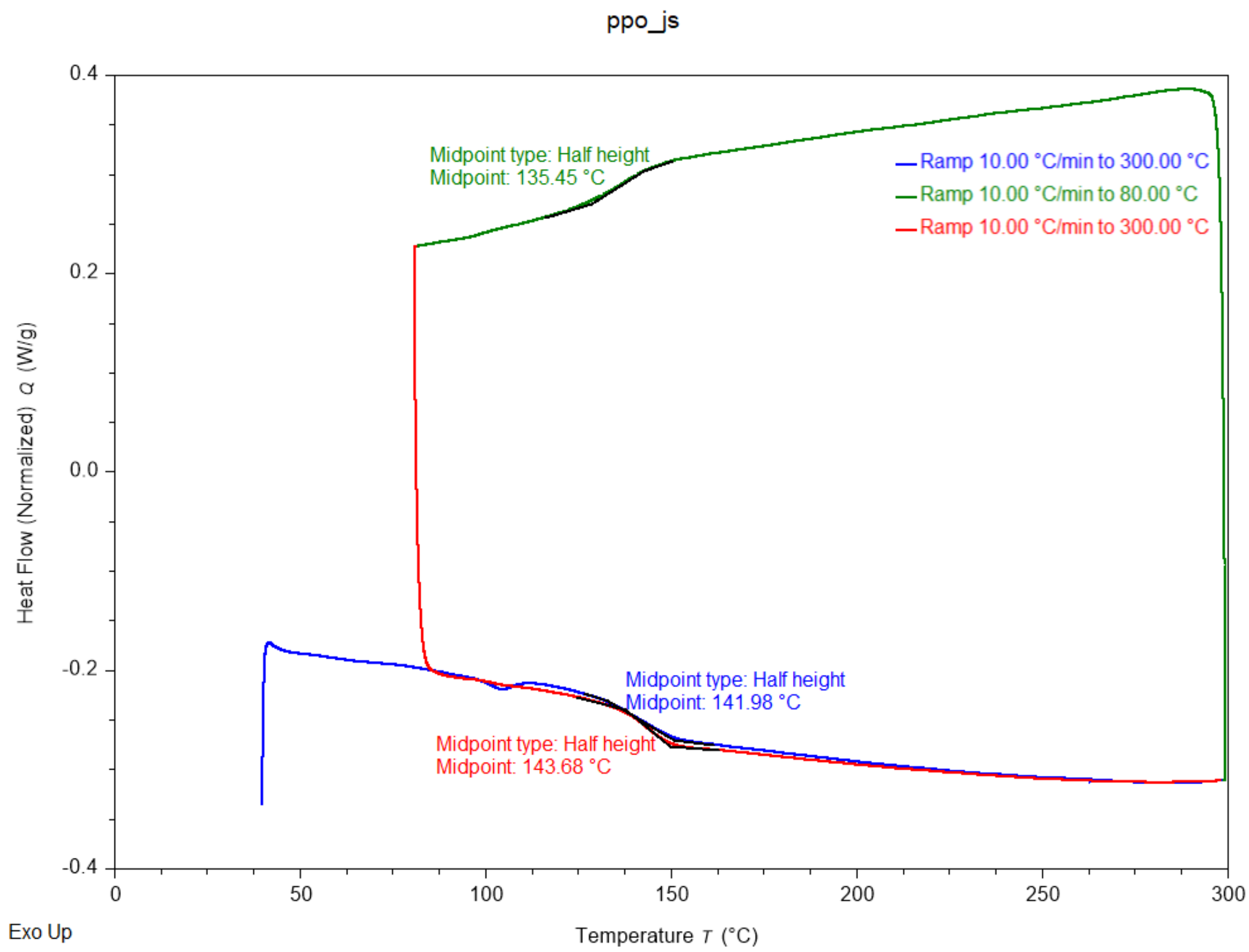

Polybutylene terephthalate (PBT):

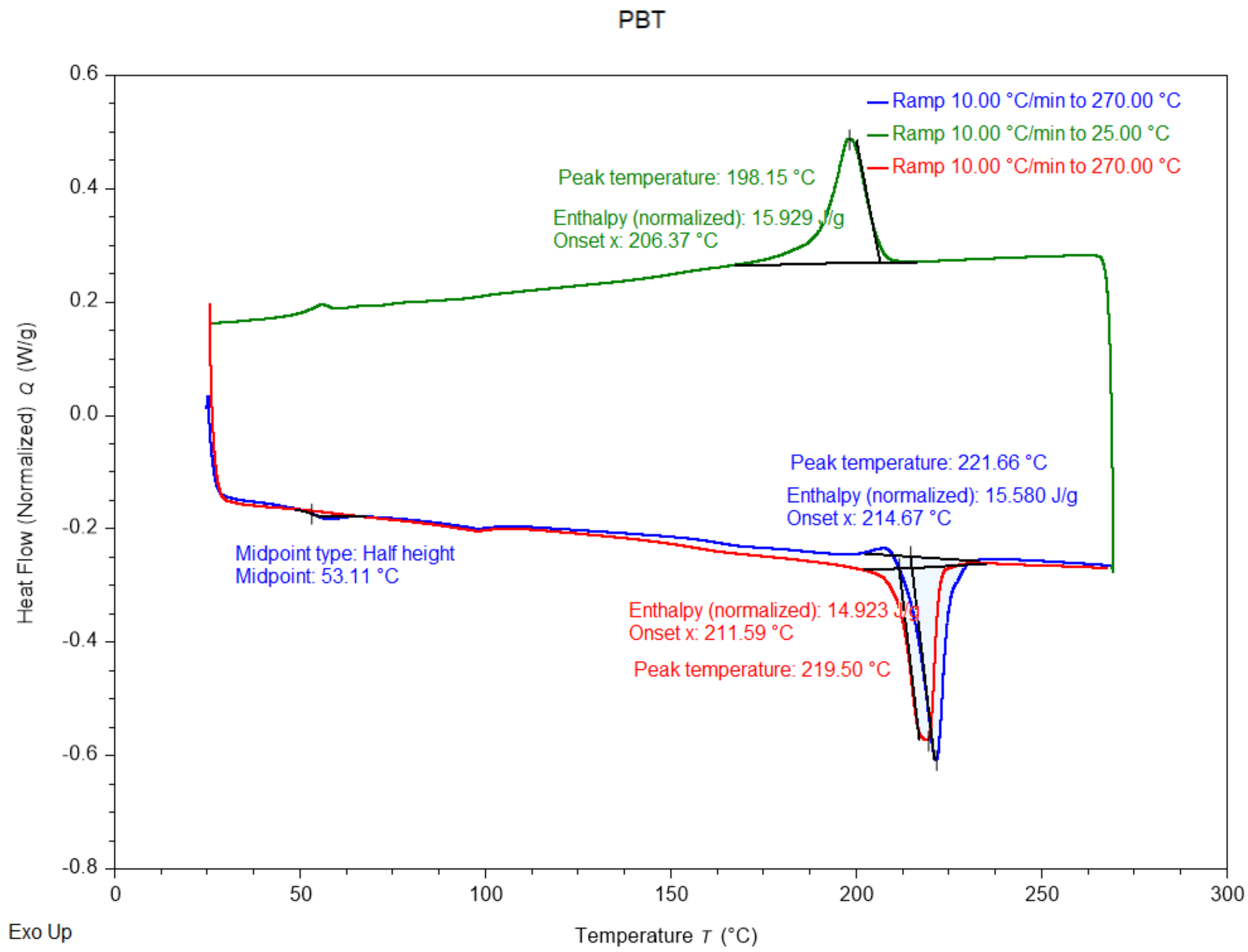

Supplementary material 3

Python code used for Model 1 and subsequent variations:

```
import numpy as np

from sklearn.preprocessing import MinMaxScaler

from xgboost import XGBRegressor


# Reading Data

train_data = "single_polymer_prediction_se/11_nylon6_less_train.txt"

test_data = "single_polymer_prediction_se/11_nylon6_only_test.txt"


# train_data = "model_amorphous_only/23_ppo_less_train.txt"

# test_data = "model_amorphous_only/23_ppo_only_test.txt"


# train_data = "model_semicrystalline_only/25_pbt_less_train.txt"

# test_data = "model_semicrystalline_only/25_pbt_only_test.txt"


# Training data normalization

training_data = np.loadtxt(train_data)

scaler = MinMaxScaler()

norm_data_train = scaler.fit_transform(training_data)

Features = norm_data_train[:, 2:13]  # 0 is index, 1 is young's modulus, 2 is stiffness, 3 is surface energy

X = Features


# Scaler for wca in the training data

scaler_wca = MinMaxScaler()

reshape_wca = np.array(training_data[:, 13]).reshape(-1, 1)

norm_wca_train = scaler_wca.fit_transform(reshape_wca)

y = norm_wca_train
```

```
model_1 = XGBRegressor(n_estimators=100, max_depth=3, eta=0.2)  # hyperparameters goes
here

model_1.fit(X, y)


# Evaluation data normalization

evaluation_data = np.loadtxt(test_data)

eval_data_reshape = evaluation_data.reshape(1, -1)

norm_data_eval = scaler.transform(eval_data_reshape)


Features_Eval = norm_data_eval[:, 2:13]

Labels_Eval = evaluation_data[13]


# Making a prediction

prediction = model_1.predict(Features_Eval)


# Prediction rescaling

prediction_reshape = prediction.reshape(-1, 1)

x_pre = scaler_wca.inverse_transform(prediction_reshape)

flat_pre = list(x_pre.flat)


# computing relative features importance

print()

pipeline_scores = model_1.feature_importances_

reshape_pipeline_scores = pipeline_scores.reshape(-1, 1)

print('Relative features importance:')

print(reshape_pipeline_scores)

print()

print("Experimental Hardness:", Labels_Eval)

print("Predicted Hardness:", x_pre)
```

Python code used for nested cross-validation

```
import numpy as np

import pandas as pd

from pathlib import Path

from sklearn.model_selection import KFold, GridSearchCV, cross_val_score

from sklearn.pipeline import Pipeline

from sklearn.preprocessing import MinMaxScaler

from sklearn.metrics import make_scorer, r2_score, root_mean_squared_error

from xgboost import XGBRegressor



def load_data(data_path: Path = 'numpy_dataset.txt'):

  data = np.loadtxt(data_path)

  X = data[:, 1:12]  # column 0 is young's modulus, column 1 is stiffness, column 2 is surface
energy

  # X = data[:, 2:12]  # column 0 is young's modulus, column 1 is stiffness, column 2 is surface
energy

  y = data[:, 12]  # column 12 is target hardness

  return X, y



def rmse(y_true, y_pred):

  return root_mean_squared_error(y_true, y_pred)



def nested_cross_validation(X, y):

  outer_cv = KFold(n_splits=5, shuffle=True, random_state=42)

  inner_cv = KFold(n_splits=4, shuffle=True, random_state=42)


  pipeline = Pipeline([

    ("scaler", MinMaxScaler()),

    ("model", XGBRegressor(random_state=42))
```

```
])

param_grid = {
    "model__n_estimators": [10, 50, 100],
    "model__max_depth": [1, 2, 3, 4, 5, 6, 7, 8, 9, 10],
    "model__learning_rate": [0.1, 0.2, 0.3, 0.4, 0.5, 0.6, 0.7],
    "model__subsample": [0.6, 0.8, 1.0],
}

grid_search = GridSearchCV(
    estimator=pipeline,
    param_grid=param_grid,
    scoring=make_scorer(rmse, greater_is_better=False),
    cv=inner_cv,
    n_jobs=-1,
    refit=True,
    verbose=1,
)

outer_scores = []
fold_results = []

for fold_index, (train_idx, test_idx) in enumerate(outer_cv.split(X), start=1):
    X_train, X_test = X[train_idx], X[test_idx]
    y_train, y_test = y[train_idx], y[test_idx]

    grid_search.fit(X_train, y_train)

    best_model = grid_search.best_estimator_
    y_pred = best_model.predict(X_test)
```

```
        test_rmse = rmse(y_test, y_pred)
        test_r2 = r2_score(y_test, y_pred)

        outer_scores.append(test_rmse)
        fold_results.append(
            {
                "fold": fold_index,
                "best_params": grid_search.best_params_,
                "test_rmse": test_rmse,
                "test_r2": test_r2,
            }
        )

        print(f"Fold {fold_index}: best params={grid_search.best_params_}, "
              f"RMSE={test_rmse:.4f}, R2={test_r2:.4f}")

    print("\nNested CV results")
    print(f"Mean RMSE: {np.mean(outer_scores):.4f}")
    print(f"Std RMSE: {np.std(outer_scores):.4f}")

    return pd.DataFrame(fold_results), np.mean(outer_scores), np.std(outer_scores)



if __name__ == "__main__":
    X, y = load_data()
    results_df, mean_rmse, std_rmse = nested_cross_validation(X, y)
    print(results_df)
nested_validation_output = pd.DataFrame(results_df)
nested_validation_output.to_excel('nested_validation_output.xlsx', index=False)
# nested_validation_output.to_excel('nested_validation_output_wo_stiffness.xlsx', index=False)
```
